# Developing Methods for Assessing Trophic Magnification of Perfluoroalkyl Substances within an Urban Terrestrial Avian Food Web

**DOI:** 10.1021/acs.est.3c02361

**Published:** 2023-08-17

**Authors:** Katharine M. Fremlin, John E. Elliott, Robert J. Letcher, Tom Harner, Frank A.P.C. Gobas

**Affiliations:** † Department of Biological Sciences, Simon Fraser University, 8888 University Drive, Burnaby, BC V5A 1S6, Canada; ‡ Ecotoxicology and Wildlife Health Division, Environment and Climate Change Canada, 5421 Robertson Road, Delta, BC V4K 3N2, Canada; § Ecotoxicology and Wildlife Health Division, National Wildlife Research Centre, Environment and Climate Change Canada, Carleton University, 1125 Colonel By Drive, Ottawa, ON K1A 0H3, Canada; ∥ Air Quality Research Division, Environment and Climate Change Canada, 4905 Dufferin Street, Toronto, ON M3H 5T4, Canada; ⊥ School of Resource and Environmental Management, Faculty of the Environment, Simon Fraser University, Burnaby, BC V5A 1S6, Canada

**Keywords:** terrestrial food web, PFAS, PFAAs, proteinophilic, trophic magnification, chemical activity

## Abstract

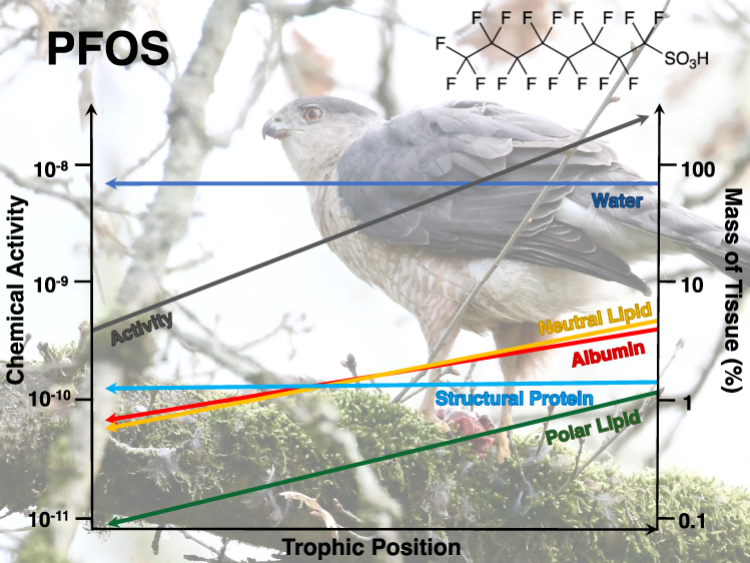

We investigated the trophic magnification potential of perfluoroalkyl substances (PFAS) in a terrestrial food web by using a chemical activity-based approach, which involved normalizing concentrations of PFAS in biota to their relative biochemical composition in order to provide a thermodynamically accurate basis for comparing concentrations of PFAS in biota. Samples of hawk eggs, songbird tissues, and invertebrates were collected and analyzed for concentrations of 18 perfluoroalkyl acids (PFAAs) and for polar lipid, neutral lipid, total protein, albumin, and water content. Estimated mass fractions of PFCA C_8_–C_11_ and PFSA C_4_–C_8_ predominantly occurred in albumin within biota samples from the food web with smaller estimated fractions in polar lipids > structural proteins > neutral lipids and insignificant amounts in water. Estimated mass fractions of longer-chained PFAS (i.e., C_12_–C_16_) mainly occurred in polar lipids with smaller estimated fractions in albumin > structural proteins > neutral lipids > and water. Chemical activity-based TMFs indicated that PFNA, PFDA, PFUdA, PFDoA, PFTrDA, PFTeDA, PFOS, and PFDS biomagnified in the food web; PFOA, PFHxDA, and PFHxS did not appear to biomagnify; and PFBS biodiluted. Chemical activity-based TMFs for PFCA C_8_–C_11_ and PFSA C_4_–C_8_ were in good agreement with corresponding TMFs derived with concentrations normalized to only total protein in biota, suggesting that concentrations normalized to total protein may be appropriate proxies of chemical activity-based TMFs for PFAS, which predominantly partition to albumin. Similarly, TMFs derived with concentrations normalized to albumin may be suitable proxies of chemical activity-based TMFs for longer-chained PFAS, which predominantly partition to polar lipids.

## Abstract 2

Trophic magnification of perfluoroalkyl substances in a terrestrial food web was investigated by measuring concentrations of 18 perfluoroalkyl acids within biota, air, and soil samples and normalizing concentrations in biota to their different tissues (e.g., protein, polar lipid, and neutral lipid).

## 1 Introduction

Poly- and perfluoroalkyl substances (PFAS) are a large group of thousands of manmade fluorinated organic chemicals commonly used in coatings for textiles, paper products, and cookware and in formulations for firefighting foams, pesticides, and hydraulic fluids.^1^ PFAS were first developed in the 1930s and have been used worldwide for 70 years, but they are generally considered to be emergent persistent organic pollutants (POPs) since they were not reported in environmental samples until the early 2000s.^1^ Also, the environmental fate and bioaccumulative behavior of a large majority of PFAS is poorly known or unknown altogether.^1^

At present, the primary endpoints and criteria currently used in Canada and the USA that define a substance as bioaccumulative include log octanol–water partition coefficients (*K*_OW_) greater than 5 and bioconcentration or bioaccumulation factors (BCF or BAF) greater than 5000. However, these metrics are typcially water-based screening thresholds so are limited in their ability to identify substances that are bioaccumulative in air-breathing organisms.^2^ For instance, unlike legacy POPs, many PFAS have low *K*_OW_ values, or their *K*_OW_ values are difficult to measure, and the ionizability or speciation of PFAA and other PFAS should also be considered when determining *K*_OW_ and other properties. Consequently, many PFAS do not biomagnify in water-breathing organisms due to high aqueous solubility and low volatility.^1,3^ However, many PFAS that exhibit low volatility can also cause low respiratory elimination rates in air-breathing organisms and thus contribute to biomagnification.^2,3^

To date, biomagnification and trophic transfer of PFAS have primarily been investigated in aquatic systems with over 30 studies completed in aquatic food webs,^3−36^ while only two studies have evaluated trophic magnification in terrestrial food webs.^37,38^ One of the major challenges faced by all these studies is accurately interpreting whether concentrations of PFAS are exhibiting biomagnification or not, which is caused by a lack of reliable methods for determining biomagnification and trophic magnification factors of PFAS in biota, particularly if organisms exhibit differences in body temperature and tissue composition. For legacy pollutants, it is common practice to calculate biomagnification and trophic magnification factors from lipid-normalized concentrations of the pollutant in the predator and prey and organisms of the food web. However, this practice cannot be applied to many PFAS because they do not preferentially accumulate in neutral or storage lipids; instead, they exhibit high affinities for protein and membrane or polar lipids.^39,40^

Currently, there is no agreed-upon method for determining whether PFAS biomagnify in terrestrial food webs or not. Hence, there is a need to develop robust approaches to accurately assess bioaccumulation of PFAS in terrestrial and air-breathing organisms. In this study, we developed and investigated new methods for determining whether PFAS biomagnify in terrestrial food webs and applied these methods to a suite of perfluoroalkyl acids (PFAAs, such as perfluorooctanesulfonic acid [PFOS] and perfluorooctanoic acid [PFOA]) measured in an urban terrestrial avian food web with the aim of producing efficient and cost-effective methods to accurately evaluate biomagnification of PFAS. Concentrations of 18 PFAAs were measured in air, soil, and tissue samples from Cooper’s hawk eggs (*Accipiter cooperii*), songbirds, and invertebrates collected across urbanized regions of Metro Vancouver, British Columbia, Canada.

## 2 Theory

Biomagnification can be defined as an increase in the chemical potential (μ), fugacity (*f*), or activity (*a*) of a contaminant in the consumer compared to that in its diet and typically occurs during food digestion and absorption.^41,42^ Biomagnification is often measured and expressed as a biomagnification factor (BMF), which is a unitless ratio between the chemical potential, fugacity, or activity in the predator and prey:
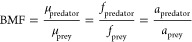
1

A BMF > 1 indicates that the substance biomagnifies in the predator, while a BMF < 1 indicates that the substance biodilutes, which may occur if the rate of biotransformation is faster than absorption of the substance in the body and/or intestinal tract of the predator.^41,42^

Trophic magnification of a substance can be defined as the general increase in the chemical potential, fugacity, or activity of a substance in animals of a food web relative to their increasing trophic positions and is caused by successive biomagnification events in the food web. Trophic magnification is measured and expressed as a unitless trophic magnification factor (TMF), which is essentially a BMF that averages all the trophic interactions across a food web.^43^ We propose that the best approach to derive TMFs is by regressing the natural logarithm of the chemical activities or fugacities in biota relative to their trophic positions (TPs) as
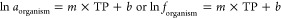
2where *m* is the slope coefficient, *b* is the *y*-intercept, and the TMF is computed as the inverse of the natural logarithm of the slope *m* (i.e., TMF = e^*m*^).^44,45^ A TMF > 1 indicates that the substance biomagnifies in the food web whereas a TMF < 1 indicates that the substance biodilutes.

### 2.1 Fugacity and Chemical Activity

Fugacity (*f*; Pa) can be expressed by the ratio between the concentration (*C*; mol/m^3^) of a substance and its fugacity capacity (*Z*; mol/m^3^ Pa) in the medium as *C*/*Z*.^46^ Chemical activity (*a*; unitless) can be expressed by the ratio between the concentration (*C*; mol/m^3^) of a substance and its solubility (*S*; mol/m^3^) in the medium as *C*/*S* for liquid substances or by *F* × *C*/*S* for substances that are solid at environmental temperatures where *F* is referred to as the fugacity ratio.^46^ As both metrics are abstract quantities, it is necessary to relate them to tangible quantities, such as measured concentrations of chemicals in organisms from field and laboratory studies. Measured concentrations can act as proxies or approximations of fugacity and chemical activity under real-world conditions since both generally have linear relationships with concentration. However, for measured concentrations of a substance in organisms to act as proxies, these concentrations need to be normalized to an organic matrix common to all organisms in the food web. By normalizing, the measured concentrations become equivalent to the fugacity capacity and/or sorptive capacity and thus become appropriate proxies of the fugacity or chemical activity when used in a biomagnification ratio. Additional theoretical details about fugacity and chemical activity, normalizing measured concentrations, and biomagnification of lipid soluble substances are provided in the Supplementary Information (SI).

### 2.2 Biomagnification of PFAS

Unlike most legacy POPs, such as polychlorinated biphenyls, PFAS typically exhibit negligible solubility in storage lipids (i.e., neutral lipids).^39,47,48^ Instead, PFAS predominately partition into phospholipids in cell membranes (i.e., polar lipids) and non-structural proteins (specifically albumin) and to a lesser extent into structural proteins (e.g., collagen, myosin, and actin).^39,40,47−49^ Thus, to account for the differing sorptive capacities of polar and neutral lipids and albumin and structural proteins, the sorptive capacity of organisms for PFAS can be expressed as
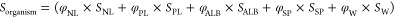
3where φ_NL_ represents the fraction of neutral or storage lipids in the organism or sample; φ_PL_ is the fraction of polar or membrane lipids; φ_ALB_ is the fraction of albumin; φ_SP_ is the fraction of structural protein; φ_W_ is the fraction of water; and *S*_NL_, *S*_PL_, *S*_ALB_, *S*_SP_, and *S*_W_ represent the solubilities of the substance in neutral lipids, polar lipids, albumin, structural proteins, and water, respectively. Since the relative partitioning of PFAS between neutral lipid, polar lipid, albumin, and structural proteins is typically measured as distribution coefficients relative to water, it may be simpler to express the solubilities *S*_NL_, *S*_PL_, *S*_ALB_, and *S*_SP_ relative to that in water (*S*_W_).^39,47,48^ Therefore, eq 3 may be modified as
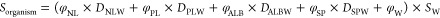
4where *D*_NLW_, *D*_PLW_, *D*_ALBW_, and *D*_SPW_ represent distribution coefficients of the substance between each tissue phase and water. Accordingly, the BMF can be derived as
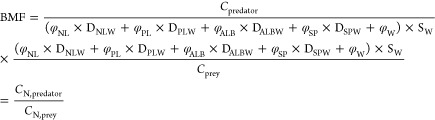
5where *C*_N, predator_ and *C*_N, prey_ represent the tissue-normalized concentrations of the chemical (e.g., mol/g normalized tissue) in the predator and prey, respectively. Similarly, the TMF is derived from a linear regression of tissue-normalized concentrations in organisms (*C*_N, organism_) as
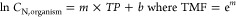
6

Another important consideration in the measurement of BMFs and TMFs for PFAS in terrestrial food webs is the body temperature of organisms. Sorptive capacities or solubilities of substances are a function of temperature;^50−53^ therefore, since body temperatures of endothermic predators (e.g., songbirds) and poikilothermic prey (e.g., earthworms) can vary substantially, the total sorptive capacities of PFAS in these organisms will likewise be different. Hence, when normalizing concentrations of substances to act as proxies for fugacity or chemical activity in organisms, sorptive capacities or distribution coefficients used in eqs 3 or 4 should be determined at the appropriate body temperature of the organism.^54^ Usually, body temperature is not considered in the derivation of BMFs and TMFs.

However, a limitation of using a fugacity- or chemical activity-based approach to characterize biomagnification of substances is the need to define not only the tissue composition of an organism but also the sorptive capacities or distribution coefficients of PFAS in the biological matrices of the organism at the appropriate temperature. This information is often not available or takes considerable effort to measure and collect. Hence, we aimed to find a relatively simple and cost-effective method for determining TMFs of PFAS from measured concentrations by investigating different tissue normalizations of measured concentrations and evaluating which produced equivalent TMFs to those derived with chemical activities. Using measured wet weight concentrations (*C*_ww_) of PFAS, we derived concentrations normalized to total protein, albumin, or polar lipids as
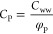

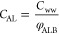

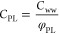
where φ_P_, φ_ALB_, and φ_PL_ represent the relative fractions of total protein, albumin, and polar lipid content, respectively, in the organism on a mass basis (i.e., g of tissue/g of sample). Normalized concentrations of PFAS were expressed as total protein equivalent concentrations (*C*_P_); albumin equivalent concentrations (*C*_AL_); and polar lipid equivalent concentrations (*C*_PL_) and account for the predominant sorption of PFAS to protein content in the organism, and more specifically to albumin, but also recognize the high sorption of PFAS to polar lipids.^39,40^

We also investigated if frequently used total lipid normalized concentrations or wet weight concentrations generated TMFs that were different from TMFs determined with chemical activities. TMFs based on wet weight concentrations do not account for differences in biochemical composition between species or samples. Total lipid-normalized concentrations (*C*_L_) were derived by dividing *C*_ww_ of PFAS by the total lipid fraction in the biota sample.

## 3 Methods and Materials

### 3.1 Study Area

The study area was approximately 336 km^2^ and separated into six sampling regions, which included urban parks and agricultural areas of Metro Vancouver, British Columbia, Canada. Each sampling region had active Cooper’s hawk nests (Figure S1). Details about the study area are included in the SI and Fremlin et al.^55^

### 3.2 Sample Collection

#### 3.2.1 Air and Soil

To evaluate the potential influence of spatial variation in contaminant concentrations, we collected soil and air samples within each sampling region. Soil samples from sampling regions were collected during invertebrate sampling from May to September 2016 and frozen at −20 °C (*n* = 12; further details in the SI). Following passive air sampling methods from Shoeib et al.,^56^ Rauert et al.,^57^ and Rauert et al.,^58^ samples were collected from six locations across Metro Vancouver from September to December 2016 (*n* = 6; Figure S2). Further details of collection, sampling rates, and preparation methods are provided in the SI.

#### 3.2.2 Biota

Our terrestrial food web included detritivores, primary and secondary consumers, and an apex predator (Figure S3, Table S1). Details of the study design, sampling locations, sampling methods, and the total number of samples collected for each trophic level are provided in Fremlin et al.^55^ and Table S1. All biota samples were collected from May to September 2016. Cooper’s hawk egg collection and animal capturing, handling, and euthanasia were approved by the Animal Care Committee of Simon Fraser University (1190-11), authorized by the Ministry of Forests, Lands and Natural Resource Operations (Surrey, BC) under permit SU16-225842, and authorized by Environment and Climate Change Canada (ECCC) under permit BC-16-0010. Further details about biota sample collection and preparation methods are provided in the SI.

### 3.3 Stable Isotope Analysis

Stable isotope (δ^15^N and δ^13^C) analyses of all biota samples were performed at the Ján Veizer Stable Isotope Laboratory at the University of Ottawa (Ottawa, ON, Canada). Details about the analyses are provided in Fremlin et al.^55^

### 3.4 Chemical Analysis

PFAA (*n* = 18) that were included in the chemical analysis are listed in Table S2. Details about biota, air, and soil sample preparation and chemical analysis are provided in the SI. Biota sample preparation and instrument analysis methods for PFAS were previously published in Braune and Letcher.^59^ Details about Standards and Chemicals (Table S3), Quality Control and Assurance (Table S4), detection and reporting limits (Tables S5 and S6), and percent recoveries are provided in the SI.

### 3.5 Tissue Composition

#### 3.5.1 Lipid Composition

Total lipid content in the biota samples was measured using a gravimetric method as reported in Fremlin et al.^55^ and is described in the SI. To determine the fraction of polar lipids in each sample, we used a Phospholipid Assay Kit (MAK122, MilliporeSigma, Sigma-Aldrich Corp., Oakville, ON). Further details about the lipid composition analysis are provided in the SI.

#### 3.5.2 Protein Composition

To determine the fraction of total protein in each biota sample, we measured the amount of total protein with a Bradford assay method.^60^ We estimated that the fraction of total protein was composed of approximately 65% albumin in the hawk eggs;^61−63^ approximately 60% albumin in the whole-body samples of the songbirds;^64−67^ and approximately 35% albumin in the invertebrate samples.^68^ The structural protein fraction in each sample was derived by subtracting the fraction of albumin from total protein. Further details about the protein composition analysis are provided in the SI.

### 3.6 Data Analysis

#### 3.6.1 Contaminant Concentrations in Air, Soil, and Biota

Geometric and/or arithmetic mean concentrations of PFAS in air, soil, and biota samples were calculated using the Nondetects and Data Analysis for Environmental Data (NADA) package^69^ or the EnvStats package^70^ in the R program^71^ if measured concentrations in samples were below the method limit of quantification (MLOQ) or the method detection limit (MDL).^72,73^ If measured concentrations of PFAS in samples were not below MLOQ or MDL (Table S4), we calculated the mean concentrations of PFAS using the EnvStats package^70^ or the dplyr package.^74^

#### 3.6.2 Spatial Analysis

For PFAS with concentrations above the sample specific detection limit (SDL; Table S5), we used a one-way Peto-Peto test (cen1way) in the NADA2 package^75^ to compare mean concentrations of PFAS in soil samples between sampling regions. Null hypotheses assumed that there was no statistical difference in the mean concentrations of PFAS in soil samples between the sampling regions. Statistical significance of *p* values for differences in mean concentrations was assessed at α = 0.05.

#### 3.6.3 Trophic Position

We characterized the TP for the species within this food web using stable nitrogen isotope (δ^15^N) measurements and a literature-based TP model, as described in Fremlin et al.^55^ The two methods provide comparable TP estimates, but we used TPs based on δ^15^N measurements and an isotopic enrichment factor of 2.88‰ for the calculations of TMFs.^55^ The isotopic enrichment factor was determined with a linear mixed effects model comparing the average δ^15^N of each organism to an average δ^15^N of its relevant prey and included sample region as a random effect.^55^

#### 3.6.4 Apparent Chemical Activity

To convert concentrations of PFAS within biota to apparent chemical activities (*a*), which are chemical activities with no specified standard reference phase for PFAS, we used eq 4 to estimate the sorptive capacity of PFAS in biota with distribution coefficients measured by Allendorf et al.^39^ at 37 °C, which is an environmentally relevant body temperature for birds (Table S7).^76−78^ PFAS lacking experimental distribution coefficients were estimated from linear regressions of experimental distribution coefficients from Allendorf et al.,^39^ Droge,^48^ and Bischel et al.^47^ and molar volume of PFAS (Figure S4; Tables S7 and S8). Separate regressions were performed for perfluorinated carboxylic acids (PFCA; Figure S5) and sulfonic acids (PFSA; Figure S6; Table S8). Water solubilities for PFAS were measured at 24–25 °C or predicted from the Open (Quantitative) Structure–Activity/Property Relationship App (OPERA; Table S2). Unfortunately, organism body temperature could not be directly considered because of a lack of predictive temperature-dependent relationships and/or measurements conducted at relevant body temperatures. Apparent chemical activity of PFAS in organisms was derived as
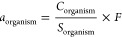
7

Since substances like PFAS are typically solids at environmental temperatures, the activity coefficient must be adjusted by *F* in order to visualize the solid chemical behaving as a semi-cooled liquid.^46,79^ The temperature dependence of *F* has a relatively minor impact on the TMF derivation since *F* does not vary substantially across the temperature range of 291–313 K used in this study. *F* also has a limited effect on the comparison between apparent chemical activities in biota since *F* essentially cancels out in a chemical activity-based biomagnification ratio. Melting points of PFAS used to estimate *F* are provided in Table S9. Organism body temperatures used to estimate *F* were 18 °C (291 K) for invertebrates^80−82^ and 40 °C (313 K) for avian species.^76−78^ Additional details about determining tissue sorptive capacities and apparent chemical activities of PFAS are provided in the SI.

#### 3.6.5 Biochemical Composition of Biota

We used separate linear regressions to examine the relationship between the fraction of neutral lipid, polar lipid, albumin, structural protein, and/or water in the biota with TP (Figure S7). Details about performing the regressions are provided in the SI. We also estimated the chemical mass distribution of PFAS in each tissue phase of the biota using measured and predicted distribution coefficients, and further details are provided in the SI.

#### 3.6.6 Trophic Magnification

We determined TMFs for PFAS using apparent chemical activities (TMF_A_), total protein equivalent concentrations (TMF_P_), albumin equivalent concentrations (TMF_AL_), total lipid equivalent concentrations (TMF_L_), polar lipid equivalent concentrations (TMF_PL_), and wet weight concentrations (TMF_W_). TMFs were determined for PFAS that were detected in more than 60% of all biota samples.^73^ To compute TMFs, we used censored regression functions in the NADA package to account for concentrations below the MLOQ. Statistical significance of *p* values was assessed at α = 0.05.

To evaluate differences between these TMF approaches, we performed a one-way analysis of variance (ANOVA) to compare the mean TMF of each method and blocked by PFAS using the Companion to Applied Regression (car) package.^83^ To identify differences between the mean TMFs and to estimate the differences between the means, we used Tukey’s multiple comparisons test.

## 4 Results and Discussion

### 4.1 Contaminant Concentrations in Air, Soil, and Biota

#### 4.1.1 PFAS in Air

Concentrations of 10 PFAS were detected in air samples from all six sampling locations; however, concentrations of PFOS were detected in air samples from only four sampling locations (Table S10; Figure S8). Geometric mean concentrations of PFAS in air samples showed limited spatial variance across the sampling regions with coefficients of variance (CVs) ranging from 9.4 to 41% (Figure S8; Table S11). Generally, geometric mean concentrations of PFCA in air were one to three orders of magnitude higher than geometric mean concentrations of PFSA (Figure S9; Table S11). PFBA and PFPeA had the highest geometric mean concentrations in air at 496 (118 SD) pg/m^3^ and 56.9 (23.3 SD) pg/m^3^, but there is considerable uncertainty in their reported concentrations since only one ion transition is monitored for PFBA and PFPeA (Figure S9). Whereas the geometric mean concentrations of PFHxA, PFHpA, PFOA, PFNA, and PFDA steadily decreased with increasing carbon chain length from 13.9 to 0.599 pg/m^3^ (Figure S9; Table S11). PFBS, PFHxS, and PFOS had geometric mean concentrations in air ranging from 0.0530 to 0.100 pg/m^3^ (Figure S9; Table S11). Since the geometric mean concentrations of PFAS in air samples exhibited limited spatial variance, the spatial differences in concentrations of PFAS across Metro Vancouver were likely marginal with limited impact on the trophic magnification assessment of PFAS. However, the concentrations of PFAS in air indicate that air is a potential exposure route of PFAS to biota, albeit a minor one compared to direct exposure from diet, water, or soil ingestion.^84^

#### 4.1.2 PFAS in Soil

The concentrations of 15 PFAS were detected in soil samples from Metro Vancouver, BC, in 2016, but only six PFAS (PFOA, PFNA, PFDA, PFUdA, PFDoA, and PFOS) had detection frequencies greater than 60% (Table S10). The Peto-Peto test did not detect any statistical differences in mean concentrations of PFOA (χ^2^ = 6.89; *p* > 0.05) and PFOS (χ^2^ = 15.2; *p* > 0.05) in soil samples between the sampling regions even though the mean concentrations of PFAS in soil samples from Richmond were up to one order of magnitude higher than in the other regions (Figure S10). The small sample size in each region (*n* = 2) reduced the statistical power to detect differences in soil concentrations between the regions.

Across the sampling regions, PFOS had the highest geometric mean concentration at 1.30 (0.51 SD) ng/g dry weight (dw) whereas PFOA, PFNA, PFDA, PFUdA, and PFDoA had geometric mean concentrations ranging from 0.0863 to 0.397 ng/g dw (Table S11; Figure S11). The geometric mean concentrations of PFOS and PFOA exhibited some spatial variance across the sampling regions with CVs of 39 and 55%, respectively. However, these spatial differences in concentrations of PFOS and PFOA across Metro Vancouver are likely small enough to not be a major confounding factor in the trophic magnification assessment. Conversely, the geometric mean concentrations of PFNA, PFDA, PFUdA, and PFDoA in soil samples across the sampling regions each exhibited high spatial variance with CVs all >100%, indicating that spatial differences in these PFAS may affect the trophic magnification assessment. However, even though there may be spatial differences in the exposure concentrations of PFAS in soil samples across regions, we did not detect any statistical differences in the TMFs of PFAS between regions (i.e., 95% CIs overlapped), which suggests that spatial differences in exposure concentrations of PFAS in soil are not confounding factors in the TMF determination in this study (Table S12).

#### 4.1.3 PFAS in Biota

The concentrations of 18 PFAS were detected in biota samples collected across Metro Vancouver, BC, in 2016, but only 12 PFAS had detection frequencies greater than 60% across all biota samples (Table S10). PFOS generally exhibited the highest mean concentrations in organisms across the food web with concentrations ranging from 0.752 ng/g wet weight (ww) in invertebrates at the base of the food web (i.e., Oniscidae) to two orders of magnitude higher in the apex predator at 138 ng/g ww (Table S13). PFCA and PFSA with carbon chains longer than eight (e.g., PFNA, PFDA, PFUdA, PFDoA, PFTrDA, PFTeDA, and PFDS) also exhibited mean concentrations in the apex predator that were one to two orders of magnitude higher than the mean concentrations in organisms at the base of the food web (Table S13). Generally, PFSA and PFCA with carbon chains shorter than eight had mean concentrations in organisms across the food web that were relatively equivalent to each other (e.g., PFOA and PFHxS) or had mean concentrations in organisms at the base of the food web that were one order of magnitude higher than the mean concentrations in the apex predator (e.g., PFBS; Table S13).

The mean concentrations of PFCAs and PFOS in terrestrial biota in this study (Table S13) were generally one to two orders of magnitude higher than the mean concentrations in the lichen–caribou–wolf terrestrial food web in the Canadian arctic, which had mean concentrations of PFOA and PFOS ranging from 0.013 to 0.23 ng/g ww and from 0.0020 to 2.2 ng/g ww, respectively.^38^ Likewise, the mean concentrations of PFOA and PFOS in aquatic organisms from marine food webs in the Canadian arctic were also often considerably lower; Kelly et al.^3^ reported concentrations of PFOA and PFOS ranging from 0.05 to 1.52 ng/g ww and from 0.21 to 37 ng/g ww, respectively, and Tomy et al.^23^ reported concentrations of PFOA and PFOS ranging from 0.59 to 16 ng/g ww and from 0.060 to 71 ng/g ww, respectively. However, the mean concentrations of PFOA and PFOS in aquatic organisms in urban^11^ and arctic^27^ freshwater food webs in Canada were often much higher than the concentrations detected in biota from our terrestrial food web. Martin et al.^11^ reported concentrations of PFOA and PFOS ranging from 1.0 to 91 ng/g ww and from 13 to 450 ng/g ww, respectively, in aquatic organisms from Lake Ontario, and Lescord et al.^27^ reported concentrations of ΣPFCA and PFOS ranging from 0.42 to 470 ng/g ww and from 0.060 to 450 ng/g ww, respectively, in aquatic organisms from arctic lakes. These concentration differences observed between terrestrial food webs and between aquatic and terrestrial food webs are likely due to differences in pollution levels, urbanization, and human population density but may also be due to differences in enzymatic elimination of PFAS between various taxa (e.g., birds versus fish).^85^

### 4.2 Biochemical Composition of Biota

#### 4.2.1 Relationship with Trophic Position

**Figure 1:**
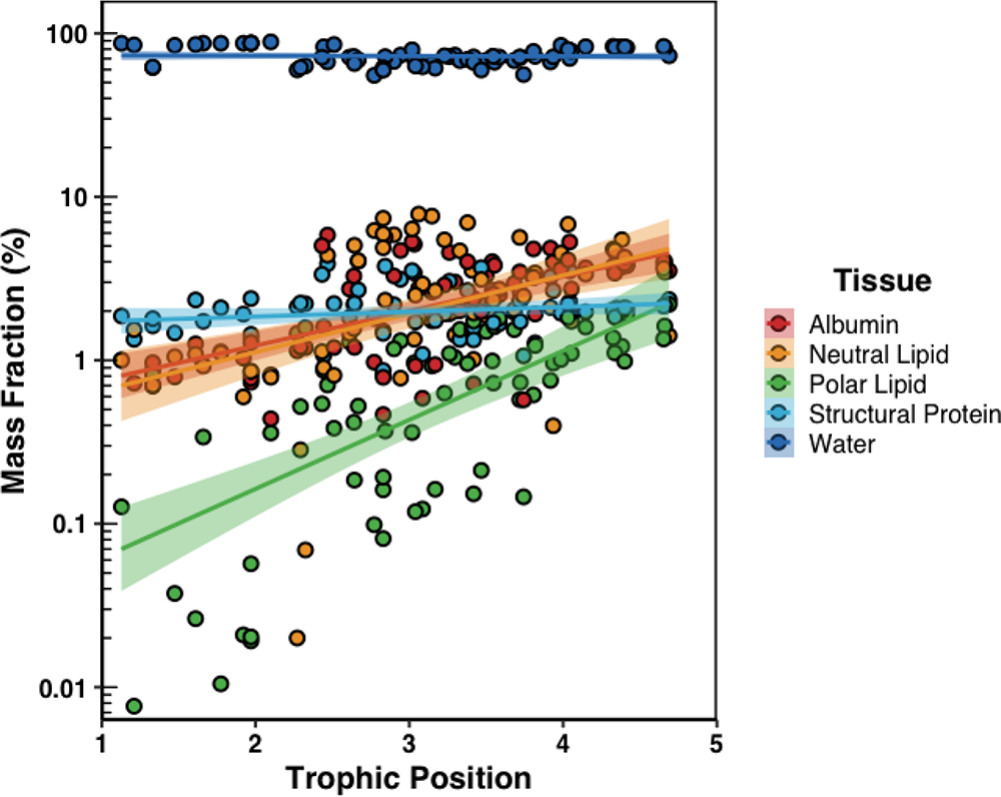
Mass fraction of each tissue (%) in biota samples relative to their trophic positions (TP). Colored lines represent respective linear regressions between TP and the mass fraction (%) of each tissue, and colored shaded areas represent the 95% confidence intervals.

Water was the dominant medium in all the biota samples ranging from 50 to 90% of the mass but did not exhibit a statistically significant linear relationship with TP (Figure 1; *F*_1,72_ = 0.195; *p* = 0.66; Table S14). Total protein comprised approximately 1–10% of the biota sample mass and exhibited a statistically significant relationship with TP (*F*_1,72_ = 19.5; *p* < 0.05; Table S14; Figure S12), which was somewhat expected since some invertebrate or insect species are limited in certain essential amino acids compared to many vertebrate species.^86^ Albumin and structural protein comprised 0.4–6 and 1–4%, respectively, of the biota mass and both increased with TP, but only albumin exhibited a statistically significant relationship (Figure 1; *F*_1,72_ = 33.9; *p* < 0.05; Table S14). Like total protein, total lipid content also comprised approximately 1–10% of the biota sample mass and exhibited a statistically significant relationship with TP (*F*_1,72_ = 30.8; *p* < 0.05; Table S14; Figure S12), which was expected since lipid content is generally known to increase with TP in other food webs.^87^ Neutral lipids comprised 0.02–8% of the biota mass and was roughly equal to the fraction of total lipids, while polar lipids comprised a much smaller mass fraction ranging from 0.001 to 2% (Figure 1). However, both neutral (*F*_1,72_ = 14.4; *p* < 0.05) and polar lipids (*F*_1,72_ = 33.9; *p* < 0.05) exhibited statistically significant relationships with TP (Table S14).

The slope coefficients for albumin (0.96 SE 0.17) and neutral lipid (0.93 SE 0.24) were equivalent indicating that the masses of these tissues both increased by approximately 1% for every integer increase in TP (Figure 1). The mass of total protein (1.1 SE 0.24) and total lipid (1.3 SE 0.24) also appeared to increase by roughly 1% for every integer increase in TP (Figure S12), whereas the mass of polar lipid (0.41 SE 0.063) roughly increased by 0.5% for every integer increase in TP (Figure 1). However, relationships with TP were strongest for polar lipids and albumin with Pearson correlations of 0.61 and 0.57, respectively, followed closely by total lipid (0.55) and total protein (0.46; Table S14). These strong relationships between the content of certain tissues in biota and TP illustrate the importance of normalizing measured concentrations of chemicals to relevant tissue components in samples to detect the occurrence of biomagnification.

#### 4.2.2 Mass Distribution of PFAS

**Figure 2:**
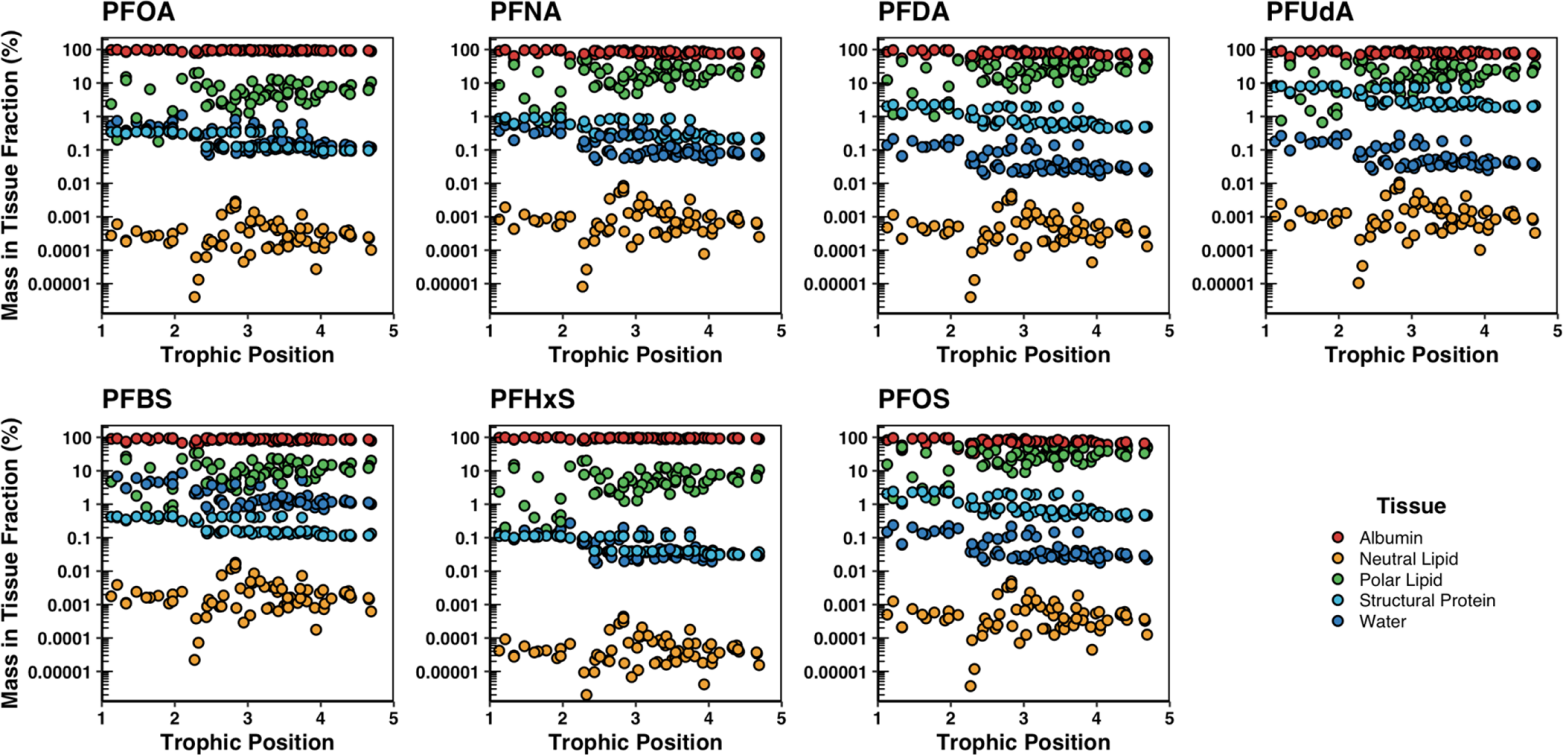
Distribution of estimated chemical mass of PFAS (%) in each tissue within the biota samples relative to their trophic position. PFAS depicted had measured experimental distribution coefficients used to model the distribution of chemical mass (Table S8).

Generally, most of the estimated mass of PFCA C_8_–C_11_ and PFSA C_4_–C_8_, which had measured distribution coefficients from Allendorf et al.,^39^ was associated with albumin (ca. 90%) in the biota samples with smaller mass fractions estimated in polar lipids (ca. 10–25%) and structural proteins (ca. 0.5–3%; Figure 2). The estimated mass of these PFAS in water and neutral lipid content was very minimal with fractions less than 0.1 and 0.001%, respectively (Figure 2). However, nearly 10% of the estimated mass of PFBS was distributed to water suggesting that this short-chained PFAS may have a greater potential for renal elimination in organisms than PFAS with larger molar volumes (Figure 2).

**Figure 3:**
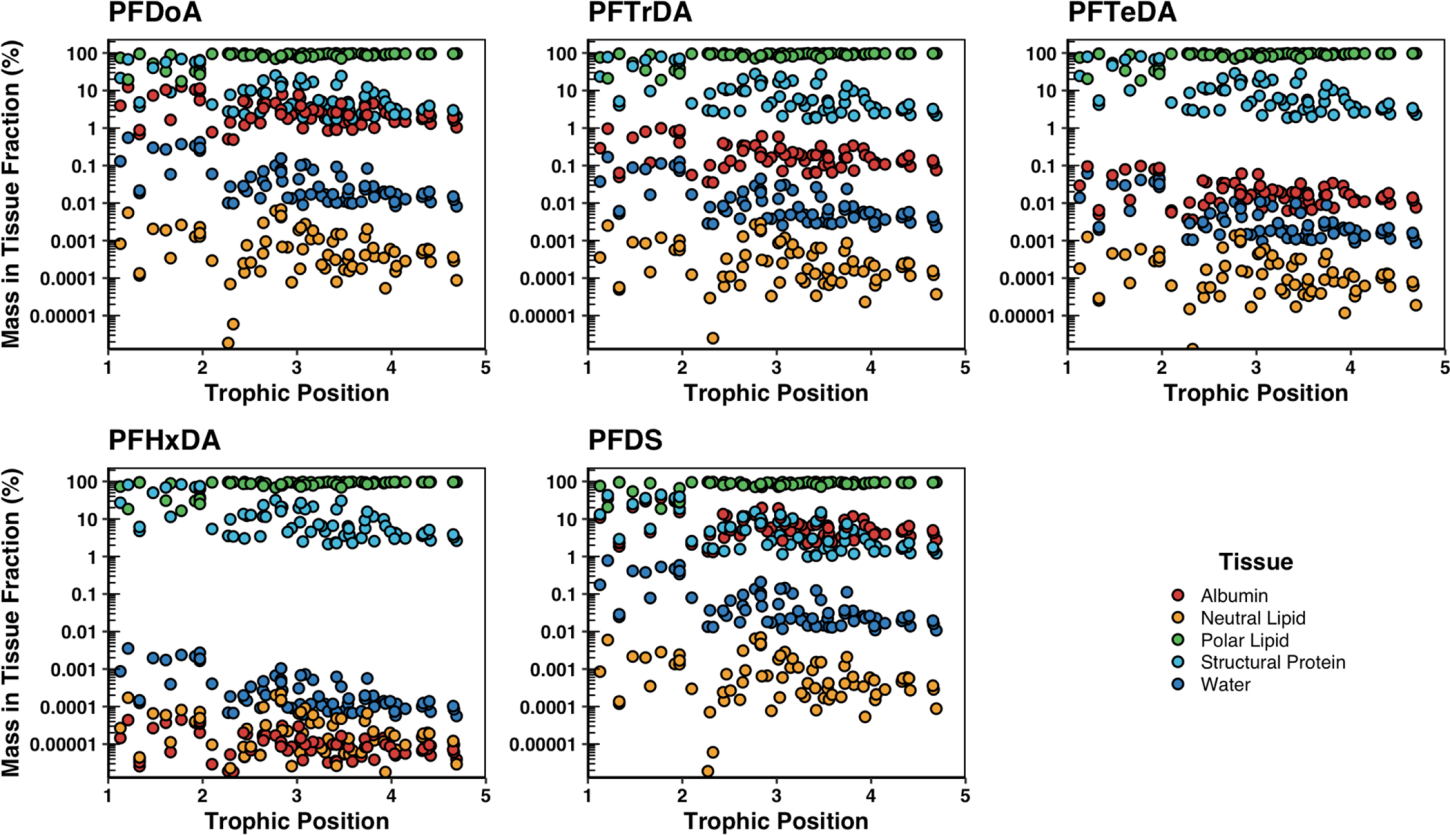
Distribution of estimated chemical mass of PFAS (%) in each tissue within the biota samples relative to their trophic position. PFAS depicted had predicted distribution coefficients used to model the distribution of chemical mass (Table S8).

In contrast, most of the estimated mass of PFCA C_12_–C_16_ and PFSA C_10_, which had predicted distribution coefficients, was distributed to polar lipids (ca. 90%) with smaller amounts to structural proteins (ca. 10–30%) and albumin (ca. 1–10%; Figure 3). The estimated mass of longer-chained PFAS in water and neutral lipids was negligible with amounts less than 0.1 and 0.001%, respectively (Figure 3). Yet, the estimated mass of PFTeDA and PFHxDA that distributed to albumin was distinctively less than 0.1 and 0.0001%, respectively (Figure 3). Hence, normalizing concentrations of PFAS to total protein, albumin, and/or polar lipids should be effective for detecting the occurrence of biomagnification of PFAS in food webs, whereas using standard lipid normalization, as commonly done for lipid-soluble POPs, will be less effective for determining biomagnification of PFAS since the mass of PFAS that distributed to neutral lipids was negligible.

However, as some measured log *D*_ALBW_ values from Allendorf et al.^39^ differ by almost an order of magnitude from values from Bischel et al.,^47^ particularly for PFDA and PFUdA (Table S7), we performed a sensitivity analysis using a range of values between the measured log *D*_ALBW_ values from both studies to see if the estimated mass distributions for PFDA, PFUdA, and PFOS would drastically change. When using log *D*_ALBW_ values from Bischel et al.,^47^ the estimated mass of PFDA, PFUdA, and PFOS in the biota generally distributed to polar lipids (ca. 80%) with smaller fractions to albumin (ca. 20%), which is roughly the opposite of what we observed with the values from Allendorf et al.^39^ However, the percent change in the TMF if using the measured log *D*_ALBW_ from Bischel et al.^47^ was very small (i.e., less than 11%), and TMFs determined for each PFAS with varying log *D*_ALW_ values did not statistically differ from each other (i.e., 95% CIs overlapped; Table S15). Thus, since using the measured log *D*_ALBW_ from either study does not statistically impact the overall biomagnification outcome, and the log *D*_ALBW_ values from Allendorf et al.^39^ were measured at 37 °C, which is an environmentally relevant body temperature for birds, the log *D*_ALBW_ values from Allendorf et al.^39^ are the more appropriate values to use for our modeling and trophic magnification assessment.

### 4.3 Trophic Magnification

**Figure 4:**
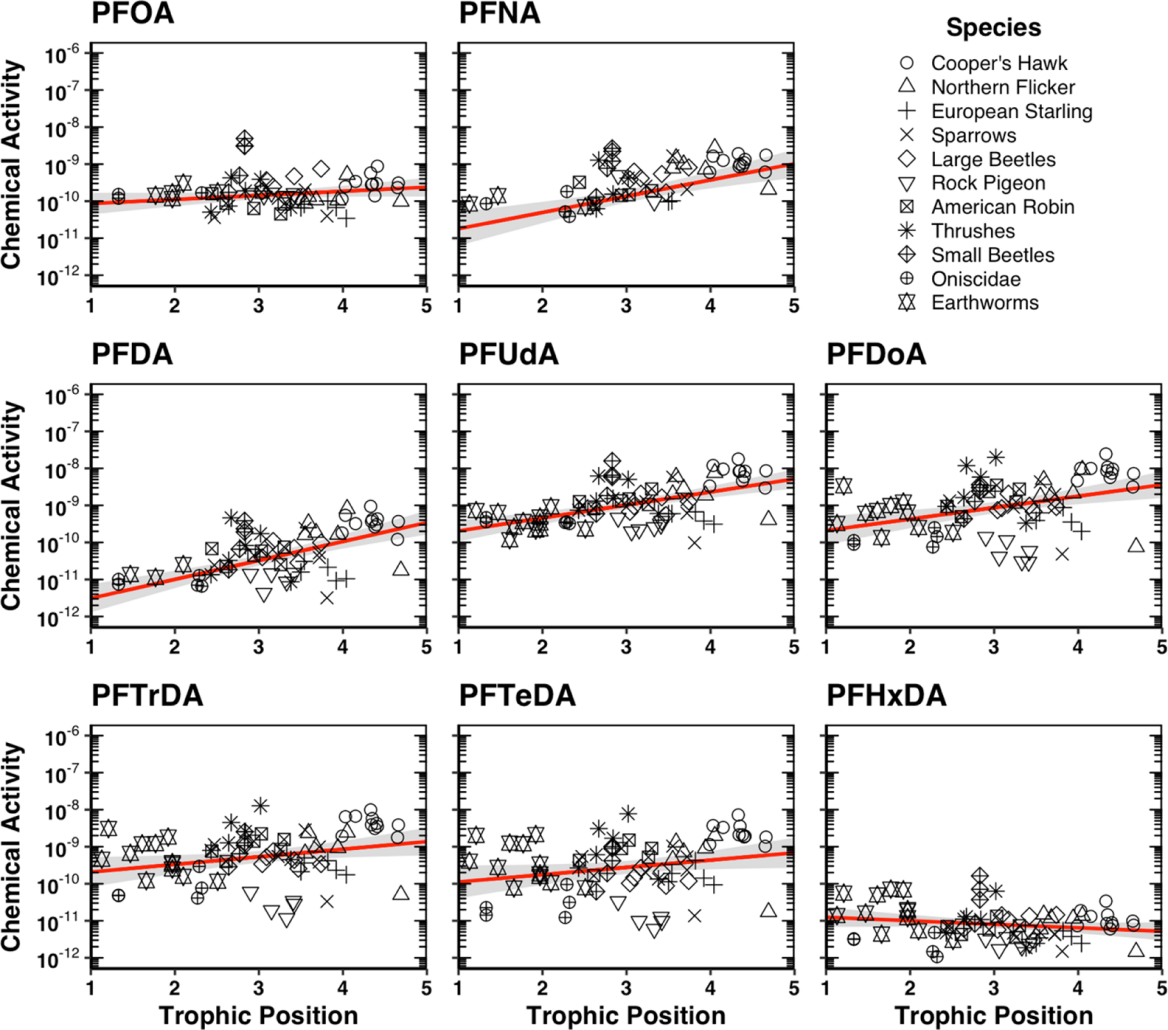
Apparent chemical activities of perfluorinated carboxylic acids in biota within an urban terrestrial food web versus relative trophic positions (TPs). Red lines represent the linear regression between the natural logarithm of the apparent chemical activities and TPs. Shaded gray areas represent the 95% confidence intervals of the slope coefficient. Data points represent concentrations above the method limit of quantification (MLOQ); concentrations below the MLOQ are not shown on plots.

**Figure 5:**
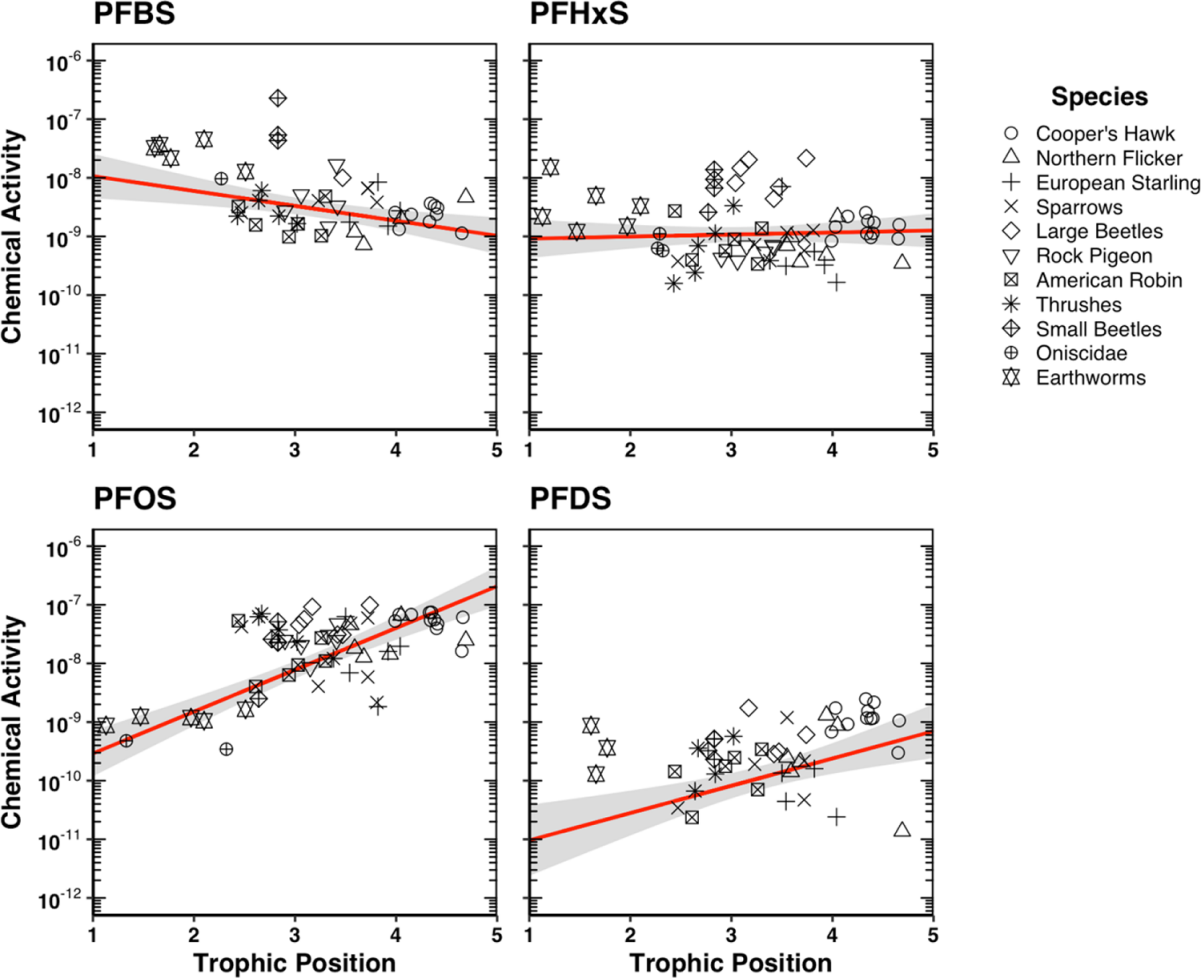
Apparent chemical activities of perfluorinated sulfonic acids in biota within an urban terrestrial food web versus relative trophic positions (TPs). Red lines represent the linear regression between the natural logarithm of the apparent chemical activities and TPs. Shaded gray areas represent the 95% confidence intervals of the slope coefficient. Data points represent concentrations above the method limit of quantification (MLOQ); concentrations below the MLOQ are not shown on plots.

TMFs determined with apparent chemical activities (TMF_A_) in the biota relative to their TPs were statistically greater than 1 (*p* < 0.05) for eight PFAS (i.e., PFNA, PFDA, PFUdA, PFDoA, PFTrDA, PFTeDA, PFOS, and PFDS), indicating that these PFAS biomagnified in this terrestrial food web (Figures 4 and 5; Table S16). PFOS exhibited the highest TMF_A_ at 5.2 (1.0 SE), while TMF_A_s of PFNA, PFDA, PFUdA, PFDoA, PFTrDA, PFTeDA, and PFDS ranged from 1.1 to 3.3 (Table S16). Apparent chemical activities of PFBS in the biota statistically decreased with TP (*p* < 0.05) and produced a TMF_A_ of 0.56 (0.10 SE), indicating that PFBS biodiluted in this terrestrial food web (Figure 5). Apparent chemical activities of PFOA, PFHxDA, and PFHxS in the biota did not statistically increase or decrease with TP (*p* > 0.05) and produced TMF_A_s approximately equal to 1, suggesting these PFAS were not biomagnifying in this food web (Figures 4 and 5; Table S16).

**Figure 6:**
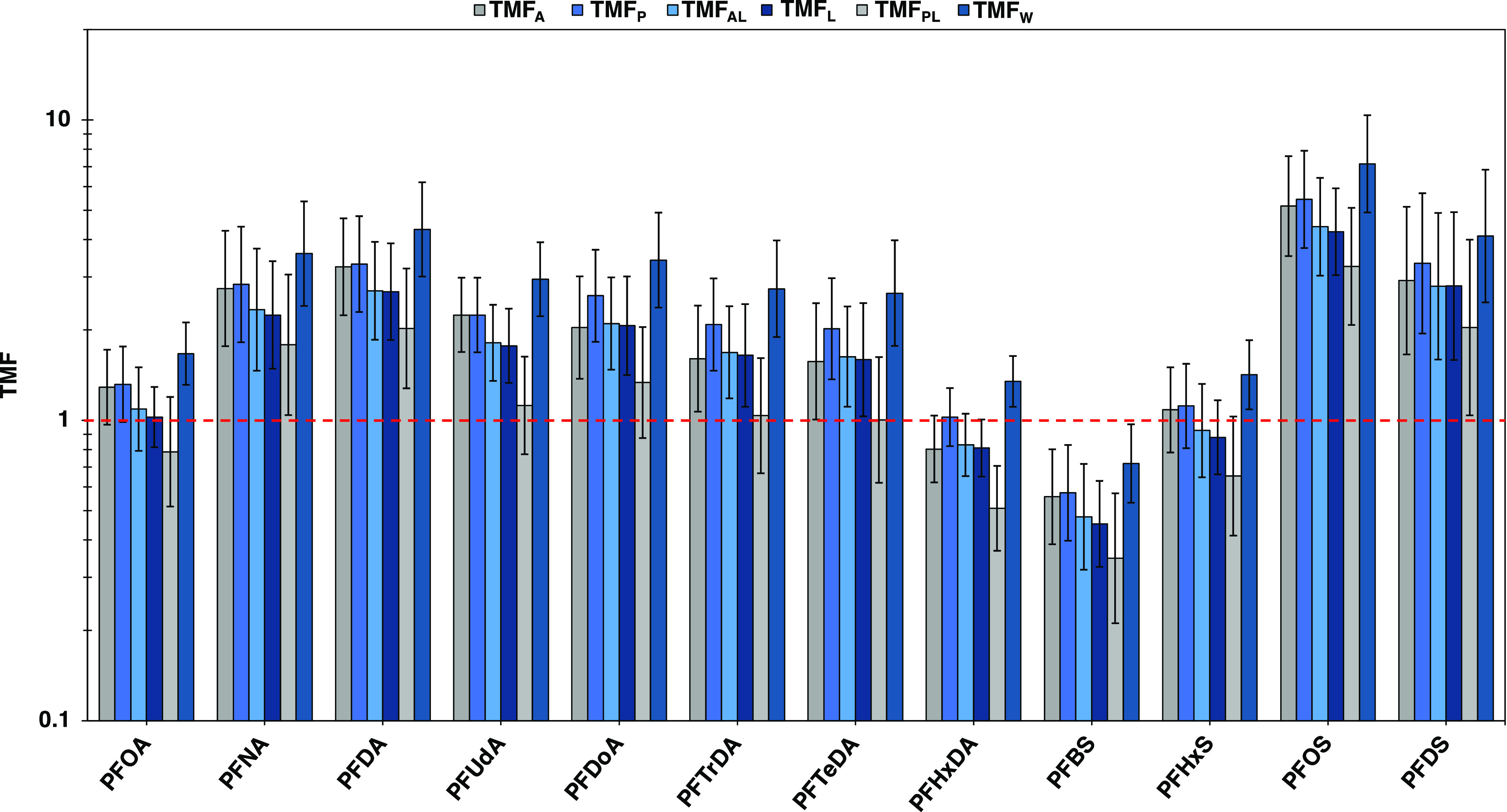
Trophic magnification factors (TMFs) of PFAS determined with apparent chemical activities (TMF_A_); concentrations normalized to total protein (TMF_P_); concentrations normalized to albumin (TMF_AL_); concentrations normalized to total lipids (TMF_L_); concentrations normalized to polar lipids (TMF_PL_); and wet weight concentrations (TMF_W_). Error bars represent the upper and lower 95% confidence interval. Red dashed line represents TMF = 1.

Generally, TMFs determined with PFAS concentrations normalized to total protein (TMF_P_) were in good agreement with TMF_A_s of PFCA C_8_–C_11_ and PFSA C_4_–C_8_ suggesting that PFAS, which associate primarily with albumin, are approximated best by total protein-normalized concentrations when deriving TMFs for food-web biomagnification assessments (Figure 6; Table S16). Conversely, TMF_P_s for longer-chained PFAS were greater than their corresponding TMF_A_s suggesting that total protein-normalized concentrations may not be reliable proxies of TMF_A_s for these PFAS (Figure 6; Table S16). However, TMFs based on concentrations normalized to albumin (TMF_AL_) and total lipid (TMF_L_) for longer-chained PFAS were both in good agreement with their corresponding TMF_A_s suggesting that concentrations normalized to albumin or total lipid may be reasonable proxies of chemical activities for longer-chained PFAS (Figure 6; Table S16). Whereas TMF_AL_s and TMF_L_s for PFCA C_8_–C_11_ and PFSA C_4_–C_8_ were generally lower than their corresponding TMF_A_s indicating that concentrations normalized to these matrices may not be appropriate proxies of chemical activities for these PFAS (Figure 6; Table S16). Similarly, TMFs based on concentrations normalized to polar lipids (TMF_PL_) for all PFAS were lower than their corresponding TMF_A_s and other TMFs likely because the fraction of polar lipids in biota was generally much smaller than the other tissue fractions and increased considerably less per TP than the other tissues (Figure 6; Table S16). Plus, most PFAS generally had higher log *D*_ALBW_ than log *D*_MLW_ values, and even though longer-chained PFAS (C_12_–C_16_) had higher log *D*_MLW_ than log *D*_ALBW_ values, this was offset by also having higher log *D*_SPW_ than log *D*_ALBW_ values (Tables S7 and S8). However, since the distribution coefficients of longer-chained PFAS were estimated rather than measured, there is greater uncertainty in their sorptive capacities and which tissue matrices are suitable proxies. Nonetheless, concentrations normalized to albumin are likely more suitable proxies for longer-chained PFAS than concentrations normalized to total lipid because neutral lipids, which comprised the majority of total lipid content in the organisms, generally have a negligible contribution to the overall sorptive capacity of PFAS.^39^

In contrast, TMFs based on wet weight concentrations of PFAS (TMF_W_s) were much higher than their corresponding TMF_A_s and other TMFs indicating that TMF_W_s generally overestimate the biomagnification potential of PFAS (Figure 6; Table S16). TMF_W_s overestimate because they ignore differences in sorptive capacities between tissues within organisms as well as differences in tissue composition between organisms. In addition, TMF_W_s had larger confidence intervals and standard errors than all corresponding TMFs indicating that TMF_W_s had less precision and greater uncertainty in estimating biomagnification of PFAS than the other TMFs (Table S16). Consequently, wet weight concentrations are not appropriate proxies of chemical activities of PFAS and are generally less accurate and precise than the other TMFs.

#### 4.3.1 Literature Comparisons

To our knowledge, 33 studies determined TMFs for PFAS in aquatic food webs using wet and/or dry weight concentrations and two studies determined TMFs for PFAS in terrestrial food webs using wet and/or dry weight concentrations (Figure S13). Most of the aquatic food webs (*n* = 24) were comprised exclusively of poikilothermic species while only nine aquatic studies included endothermic species. Including this study, only three studies have determined TMFs for terrestrial food webs with endothermic species, highlighting a need for further research of terrestrial systems with air-breathing organisms.

Generally, TMF_W_s (or TMFs based on dry weight concentrations [TMF_D_s]) of PFAS in our terrestrial food web were greater than or equivalent to corresponding TMF_W_s reported in the lichen–caribou–wolf food web in the Canadian arctic^38^ and the terrestrial avian food web on the Tibetan plateau^37^ (Table S17). TMF_W_s and/or TMF_D_s in terrestrial avian food webs were often two to three times greater than corresponding TMF_W_s in the terrestrial mammalian food web, and terrestrial avian food webs typically had a greater number of PFAS exhibiting biomagnification (Table S17). Greater biomagnification observed in food webs with avian apex predators than in food webs with mammalian predators is consistent with greater energy demands and body temperatures observed in birds.^77,78,87^

With the exception of Kelly et al.^3^ and Munoz et al.,^34^ most food-web biomagnification studies did not normalize concentrations of PFAS to tissue phases in biota. Consequently, only three studies, including this study, have determined TMFs using PFAS concentrations normalized to total protein. TMF_P_s for PFAS reported by Kelly et al.^3^ in an aquatic food web with avian and marine mammal species were within a factor of 2 to corresponding TMF_P_s in our terrestrial food web, demonstrating good agreement between food webs with endothermic species (Table S17). Whereas TMF_P_s for PFAS reported by Munoz et al.^34^ in an aquatic food web with only poikilothermic species were almost an order of magnitude lower than the corresponding TMF_P_s in the food webs with endothermic organisms, indicating that PFAS have less tendency to biomagnify in food webs without endothermic organisms (Table S17).

TMF_P_s from the terrestrial and aquatic food webs were generally lower than their corresponding TMF_W_s (Table S17). However, some TMF_P_s for PFAS reported by Munoz et al.^34^ were often equivalent to their corresponding TMF_W_s likely because all the vegetation samples with low protein content were included in the regressions for TMF_W_s but excluded in the TMF_P_s. But if all the samples had been included in the TMF_P_s, all the reported TMF_P_s would likely have been lower than their corresponding TMF_W_s. This disparity illustrates how TMFs based on wet weight concentrations disregard differences in protein content between samples or organisms and subsequently overestimate the biomagnification potential of PFAS.

### 4.4 Methodological Implications of PFAS Biomagnification in Food Webs

It is evident that using appropriate normalizations of PFAS concentrations in biota is a key component to consider when developing methods for determining whether a PFAS biomagnifies or not. Following a chemical activity-based approach is the preferred method for determining the biomagnification potential of PFAS in terrestrial or aquatic food webs since it can account for differences in sorptive capacities between tissues within organisms and for differences in body temperature between organisms. However, the chemical activity-based approach generally requires considerable effort so development of simple, alternative methods that produce equivalent biomagnification results is needed.

For method development, we demonstrated that the fractions of total protein, albumin, neutral lipids, and polar lipids in the biota samples of this food web each had strong linear relationships with TP, indicating that normalization of chemical concentrations in biota with these matrices would be effective for detecting the occurrence of biomagnification of substances that readily partition into them. Additionally, by estimating the mass of PFAS within each tissue phase of the biota samples with measured and predicted distribution coefficients, we illustrated that many PFAS appear to primarily partition to albumin with lesser amounts to polar lipids and structural proteins but that these mass distributions can also vary among different PFAS depending on their albumin–water distribution coefficients. Consequently, PFAS that primarily partition to albumin were approximated best by TMFs based on concentrations normalized to total protein, whereas PFAS that primarily partition to polar lipids were approximated best by TMFs based on concentrations normalized to albumin. Conversely, TMFs based on commonly used wet weight concentrations of PFAS generally overestimated the biomagnification potential of PFAS compared to corresponding TMFs based on apparent chemical activities or normalized concentrations, which was also observed in aquatic food webs.

Additionally, a one-way ANOVA revealed that there was evidence that the mean TMFs determined for each method were not all equal (*F*_16,55_ = 68.03, *p* < 0.001, *r*^2^ = 0.94; Figure S14). The mean TMF_W_ was statistically greater than all the other mean TMFs, the mean TMF_PL_ was statistically less than all the other mean TMFs, and the mean TMF_L_ was statistically greater than the mean TMF_P_ and the mean TMF_AL_ (Figure S14). In contrast, the mean TMF_A_ was not statistically different from the mean TMF_P_, mean TMF_AL_, or mean TMF_L_ indicating that concentrations of PFAS normalized to total protein or albumin are equivalent proxies to corresponding chemical activities (Figure S14).

To evaluate which PFAS these two normalization methods will be more applicable for, we examined the relationships between the natural logarithm of the observed TMFs and the physicochemical properties of PFAS, specifically molar volume, log *D*_ALW_, and log *D*_MLW_ (Figure S15). Based on the overlap between the fitted relationship for TMF_P_s or TMF_AL_s with the fitted relationship for TMF_A_s, PFAS with a molar volume greater than 320 cm^3^/mol, a log *D*_ALW_ less than 4, or a log *D*_MLW_ greater than 5 will be approximated best by concentrations normalized to albumin while PFAS with a molar volume less than 320 cm^3^/mol, a log *D*_ALW_ greater than 4, or a log *D*_MLW_ less than 5 will be approximated best by concentrations normalized to total protein.

Hence, as a practical alternative to the chemical activity-based approach, we recommend normalizing concentrations of PFAS in biological samples to total protein and/or albumin content to assess the food-web bioaccumulation potential of PFAS, particularly since the assays to measure total protein and albumin are relatively simple and inexpensive. Our results indicate that normalization to total protein and albumin content are both effective methods for accurately detecting biomagnification of PFAS in food webs but application can depend on the physicochemical properties of PFAS like molar volume. Also, if different organs/tissues, such as liver, blood, or egg, are sampled from organisms of the food web, normalization by total protein or albumin is essential for the TMF analysis since samples from different organs/tissues will often have a higher protein or albumin content than samples from the whole body.^88^ Whereas TMFs based on wet weight concentrations will ignore these differences in protein content between samples or organisms, and thus can misidentify bioaccumulation potential for substances that predominately partition into proteins.

## Supporting Information Available

Additional details of materials, methods, and data analysis results including figures and tables (PDF)
All data collected and used (XLSX)
