# Supplementary material for: Developing Methods for Assessing Trophic Magnification of Perfluoroalkyl Substances within an Urban Terrestrial Avian Food Web
Source: Environ Sci Technol. 2023 Aug 17;57(34):12806–18. doi: 10.1021/acs.est.3c02361 (PMC10469464; doi:10.1021/acs.est.3c02361)
Supplement: Supplementary file 1 — es3c02361_si_001.pdf [file es3c02361_si_001.pdf]

Developing methods for assessing trophic magnification of perfluoroalkyl substances within an urban terrestrial avian food-web

Kate M. Fremlin[a,b*], John E. Elliott[b,a], Robert J. Letcher[c], Tom Harner[d], and Frank A.P.C. Gobas[e,a]

[a]Simon Fraser University, Dept. of Biological Sciences, 8888 University Drive, Burnaby, British Columbia, V5A 1S6, Canada: kfremlin@sfu.ca
[b]Environment and Climate Change Canada, Ecotoxicology and Wildlife Health Division, 5421 Robertson Road, Delta, British Columbia, V4K 3N2, Canada: john.elliott@ec.gc.ca
[c]Environment and Climate Change Canada, Ecotoxicology and Wildlife Health Division, National Wildlife Research Centre, Carleton University, Ottawa, ON: robert.letcher@ec.gc.ca
[d]Environment and Climate Change Canada, Air Quality Research Division, 4905 Dufferin Street, Toronto, Ontario, M3H 5T4, Canada: tom.harner@ec.gc.ca
[e]School of Resource and Environmental Management, Faculty of the Environment, Simon Fraser University, Burnaby, British Columbia, Canada V5A 1S6: gobas@sfu.ca

Supplementary Information

Pages: 52; Figures: 15; Tables 17

List of Figures

Figure S1. Study area separated into six sampling regions with Cooper's hawk nests and nesting territories in Metro Vancouver, British Columbia, 2016. Map created using the Free and Open Source QGIS. 7

Figure S2. Locations of passive air samplers in relation to Cooper's hawk nest sites across Metro Vancouver, British Columbia, 2016. Map created using the Free and Open Source QGIS. 8

Figure S3. Urban terrestrial avian food-web with generalised trophic linkages among organisms collected in Metro Vancouver, BC in 2016. 10

Figure S4. Scatterplot illustrating relationships between the physical properties and measured distribution coefficients of PFAS. Chain = carbon chain length, Vm = molar volume (cm^3/mol); F_no = number of attached fluorine; MW = molar weight (g/mol); Log DMLW = Membrane or polar lipid-water (L/L); Log DALBW = Albumin-water (L/L); Log DSPW = Structural protein-water (L/L); and Log DSLW = storage or neutral lipid-water (L/L). 24

Figure S5. Relationships between measured distribution coefficients and molar volume of PFCA used to predict distribution coefficients for PFCA. Blue lines represent the linear regressions, and grey shaded areas represent the 95% confidence interval of the slope. 25

Figure S6. Relationships between measured distribution coefficients and molar volume of PFSA used to predict distribution coefficients for PFSA. Blue lines represent the linear regressions, and grey shaded areas represent the 95% confidence interval of the slope. 26

Figure S7. Pairplot of all variables - trophic position (TP) and the respective tissue components in each sample. Lower panels contain scatterplots with a LOESS smoother (red line) added to aid visual interpretation. Upper panels contain estimated pair-wise correlations with size proportional to the absolute correlation coefficient. Diagonal panels contain histograms. 28

Figure S8. Concentrations (pg/m^3) of PFAS detected in air samples from six locations (n = 6) across Metro Vancouver, BC from September – December 2016. One sample was collected in each sampling region. NVAN = North Vancouver; NBBY = North Burnaby – East Vancouver; VANW = Vancouver – West; VANS = Vancouver – South; RICH = Richmond; DELT = Delta. ★ represent sample concentrations that were below the limit of detection. 30

Figure S9. Geometric mean concentrations (pg/m^3) of PFAS in air samples from six locations (n = 6) across Metro Vancouver, 2016. Error bars represent the upper and lower 95% confidence intervals. 31

Figure S10. Mean concentrations (ng/g dry weight) of PFAS in soil samples (n = 2 per sampling region) collected from Metro Vancouver, BC, 2016. NVAN = North Vancouver; NBBY = North Burnaby – East Vancouver; VANW = Vancouver – West; VANS = Vancouver – South; RICH = Richmond; DELT = Delta. Peto-Peto test did not detect any differences in the mean concentrations of PFOA and PFOS in the soil samples between the sampling regions. PFOA (χ^2 = 6.89; p = 0.229) and PFOS (χ^2 = 15.2; p = 0.01; pairwise comparisons $p > 0.05$). 33

Figure S11. Geometric mean concentrations (ng/g dw) of PFAS in soil samples (n = 12) from Metro Vancouver, BC, 2016. Error bars represent the upper and lower 95% confidence limits. PFAS displayed had ≥ 60% detection frequency across all soil samples. 34

Figure S12. Mass fraction (%) of each tissue in biota samples relative to their trophic positions (TP). Coloured lines represent linear regressions between TP and the mass fraction (%) of each respective tissue. 37

Figure S13. Number of studies that have determined TMFs based on wet and/or dry weight concentrations of PFAS in aquatic and terrestrial food-webs with either exclusively poikilothermic organisms or a combination of endothermic and poikilothermic organisms. Aquatic studies which included poikilothermic species: Bergman [56], Chen, et al. [57], Chu, et al. [58], Fang, et al. [59], Li, et al. [60], Loi, et al. [61], Martin, et al. [62], Mazzoni, et al. [63], Miranda, et al. [64], Munoz, et al. [65], Munoz, et al. [66], Pan, et al. [67], Penland, et al. [68], Ren, et al. [69], Ren, et al. [70], Simonet-Laprade, et al. [71], Simonnet-Laprade, et al. [72], Teunen, et al. [73], Zhang, et al. [74], Houde, et al. [75], Langberg, et al. [76], Du, et al. [77], and Wang, et al. [78]; and aquatic studies which included endothermic and poikilothermic species: Chen, et al. [79], Kelly, et al. [80], Tomy, et al. [81], Xu, et al. [82], Zhang, et al. [74], Houde, et al. [83], Tomy, et al. [84], Gao, et al. [85], and Xiong [86]. Terrestrial studies included Huang, et al. [87], Müller, et al. [88], and this study. 42
Figure S14. (Left) Comparison of mean TMFs averaged over all PFAS with compact letter display. Bars represent least square (LS) mean value for each TMF method following a one-way analysis of variance (ANOVA). Error bars indicate 95% confidence intervals of the LS means. Means sharing a letter are not statistically different ($\alpha = 0.05$, Tukey-adjusted). (Right) Estimated pairwise differences in mean TMFs averaged over all PFAS.. 45
Figure S15. Relationships between observed TMFs and physicochemical properties of PFAS. Coloured solid lines represent separate regressions for all PFAS analysed with each TMF method (TMF_A = TMF based on apparent chemical activities, TMF_P =TMF based on concentrations normalised to total protein, TMF_{AL} =TMF based on concentrations normalised to albumin, TMF_{PL} =TMF based on concentrations normalised to polar lipids, TMF_W =TMF based on wet weight concentrations). Red dashed line represents TMF = 1. D_{ALBW} = distribution coefficient of albumin-water and D_{MLW} = distribution coefficient of membrane or polar lipid-water. 46

List of Tables

Table S1. Species selected from the urban terrestrial food web in Fremlin, et al. [11] with the number of eggs (hawk) or composite samples analysed for PFAS in 2020. 11
Table S2. Perfluoroalkyl acids (PFAA) and corresponding internal standards that were included in the chemical analysis. Physicochemical properties were obtained from the CompTox Chemicals Dashboard for neutral molecules.[37, 38] Values are experimental averages (in black) or predicted averages (in red) from the Open (Quantitative) Structure-Activity/Property Relationship App (OPERA)*. MW = Molecular weight; VP = Vapour pressure; H = Henry's Law Constant; S_W = Water solubility; K_{OW} = Octanol-water partition coefficient. 13
Table S3. PFAA chemicals with names, abbreviations, associated surrogate/injection standard, and quantifier transition used in processing and analysis of PUF disks. 14
Table S4. Mean, Min, and Max concentrations of PFAS in field and/or procedural blanks for air and biota samples. 18
Table S5. Limit of detection (LOD) and method detection limit (MDL) of PFAS within air samples at HAPs Lab determined with average air volume (m^3) of six samples. Method limit of detection (MLOD) and method limit of quantification (MLOQ) of PFAS within respective biota samples at NWRC. NA = Not analysed. 19
Table S6. Reporting limits (RL) of PFAS based on the lowest calibration standard analysed in a 5 g dw soil sampl); % recovery in spiked matrix sample; and sample specific detection limits (SDL) of

PFAS in the two soil samples from each sampling region corrected for sample specific weight. NVAN = North Vancouver; NBBY = North Burnaby – East Vancouver; VANW = Vancouver – West; VANS = Vancouver – South; DELT = Delta; RICH = Richmond. RL also equal to concentration in lab blank (*n*=1). 20

Table S7. Measured tissue-water distribution coefficients of PFAS obtained from Allendorf, et al. [16], Bischel, et al. [17], and Droge [18] used to predict distribution coefficients of PFAS that were not measured or analysed (i.e., NA). Chain = carbon chain length; F_N = number of attached fluorine; V_M = molar volume (cm^3/mol); MW = molar weight (g/mol); D_{MLW} = Membrane or polar lipid-water; D_{ALBW} = Albumin-water; D_{SPW} = Structural protein-water; D_{SLW} = storage or neutral lipid-water. Values for V_M and MW were obtained from the CompTox Chemicals Dashboard for neutral molecules. [37, 38] 23

Table S8. Measured tissue-water distribution coefficients of PFAS from Allendorf, et al. [16] and predicted values used to determine apparent chemical activities. V_M = molar volume; D_{MLW} = Membrane or polar lipid-water; D_{ALBW} = Albumin-water; D_{SPW} = Structural protein-water; D_{SLW} = storage or neutral lipid-water. 24

Table S9. Melting points (T_M), water solubilities (S_W), and tissue solubilities (ALB = Albumin; PL = Polar Lipids; NL = Neutral Lipids; and SP = Structural Protein) used to convert concentrations of PFAS in biota to apparent chemical activities. 27

Table S10. Detection frequencies (%) of PFAS concentrations analysed in air, soil, and biota samples collected across Metro Vancouver, BC, in 2016. NA = Not analysed. 29

Table S11. Geometric mean concentrations of PFAS in air (n = 6) and soil samples (n = 12) from six sampling regions of Metro Vancouver, 2016. SD = Standard Deviation; LCL = Lower 95% Confidence Limit; UCL = Upper 95% Confidence Limit. 32

Table S12. Trophic magnification factors (TMFs; [95% LCL, UCL]) based on wet weight concentrations of PFAS determined for each sampling region. Overlap between the 95% CIs indicates that there were no statistical differences in the TMFs for PFAS between regions. 35

Table S13. Mean concentrations (ng/g ww) of PFAS within organisms from a terrestrial food web in Metro Vancouver, BC, 2016. Mean concentrations (SE) were estimated with the Kaplan-Meier method in NADA. WB = whole body; TP = mean trophic position (± SD); ND = non-detection. 36

Table S14. Linear relationships between trophic position and the mass fraction (%) of each respective tissue in the biota samples. Values in bold indicate statistical significance of p-value with $\alpha < 0.05$. 37

Table S15. Trophic magnification factors (TMFs) for PFAS based on apparent chemical activities (TMF_A) determined using a range of log D_{ALBW} values between the measured value from Bischel, et al. [17] and the measured value from Allendorf, et al. [16]. 38

Table S16. Trophic magnification factors (TMFs) for PFAS based on apparent chemical activities (TMF_A); concentrations normalised to total protein (TMF_P); concentrations normalised to albumin (TMF_{AL}); concentrations normalised to total lipid (TMF_L); concentrations normalised to polar lipids (TMF_{PL}); wet weight concentrations (TMF_W); and dry weight concentrations (TMF_D). SE = standard error, LCL = Lower 95% confidence limit, UCL = Upper 95% confidence limit. Values in bold indicate statistical significance of p-value with $\alpha < 0.05$. 39

Table S17. Trophic magnification factors of PFAS from terrestrial and aquatic food-webs determined with wet weight concentrations (TMF_W), dry weight concentrations (TMF_D), and concentrations normalised to total protein content (TMF_P). 43

THEORY

Fugacity and Chemical Activity

Fugacity and chemical activity are complementary concepts developed by G. N. Lewis in 1901 and used in the field of thermodynamics to characterize a chemical's capacity for transport and transformation and predict its environmental fate.[1, 2] Defining the BMF and TMF in both fugacity and chemical activity formats is advantageous as it enables some substances, such as those with a significant presence in the gas phase, to be treated with the fugacity approach while other substances, which do not readily partition to the gas phase but have a considerable presence in water, are more easily treated with the chemical activity approach.[3, 4]

Normalising measured concentrations to act as proxies of fugacity or chemical activity does not require the selection of a specific standard reference state, which is difficult to determine for PFAS as PFAS generally occur in ionic form, since it cancels out in a fugacity or chemical activity ratio. Thus, when referring to chemical activity we are referring to apparent chemical activity rather than chemical activity to avoid specifying a standard reference phase for PFAS.

Biomagnification of Lipid Soluble Substances

Since lipid-soluble substances, such as polychlorinated biphenyls and other legacy POPs, preferentially partition into lipids in organisms, BMFs of lipid-soluble substances are usually derived from lipid normalised concentrations.[5-11] Thus, equation 1 is modified as:

(1) $$BMF = \frac{a_{predator}}{a_{prey}} = \frac{C_{predator}}{S_{predator}} \times \frac{S_{prey}}{C_{prey}} = \frac{C_{predator}}{S_L \times \varphi_{L,predator}} \times \frac{S_L \times \varphi_{L,prey}}{C_{prey}} = \frac{C_{L,predator}}{C_{L,prey}}$$

where $S_{predator}$ and S_{prey} represent the sorptive capacity of the chemical in the organism; S_L represents the sorptive capacity of lipids; φ_L the fraction of total lipid content; and $C_{L,predator}$ and $C_{L,prey}$ are the lipid normalised concentrations of the chemical (e.g., mol/g lipid) in the predator and prey. Lipid normalisation is usually a standard practice when investigating biomagnification (e.g., OECD 305 guidelines [12]) and trophic magnification of lipid-soluble substances in organisms and is generally based on results from aquatic organisms and food-webs.[13-15] Accordingly, the TMF of lipid-soluble substances is derived from a linear regression of lipid normalised concentrations in organisms ($C_{L,organism}$) as:

(2) $$ln\ C_{L,organism} = m \cdot TP + b \quad \text{where} \quad TMF = e^m$$

This normalization approach inherently assumes that (i) the sorptive capacity of the organism is chiefly represented by lipids and directly proportional to the fraction of lipid content in the organism and (ii) that the sorptive capacity of the substance in lipids for all organisms is equivalent. However, these assumptions are not always applicable. For instance, if lipid content within organisms is low, then non-lipid organic matrices, such as proteins, will become the main site of chemical bioaccumulation.[8] In such cases, lipid normalisation can overestimate the fugacity and concentration in organisms with low lipid content and subsequently underestimate trophic magnification since many lower trophic level organisms have low lipid content.

Moreover, lipid normalisation will also generally fail to accurately characterize BMFs and TMFs for substances that are poorly soluble in storage lipids (i.e., neutral lipids), since storage lipids often comprise most of the lipid content in organisms. PFAS are prime examples of substances that are poorly soluble in neutral lipids.[16-18] Instead, PFAS predominantly partition into phospholipids in cell membranes (i.e., polar lipids) and non-structural proteins (specifically albumin) and to a lesser extent into structural proteins (e.g., collagen, myosin, and actin).[16-20]

METHODS & MATERIALS

Study Area

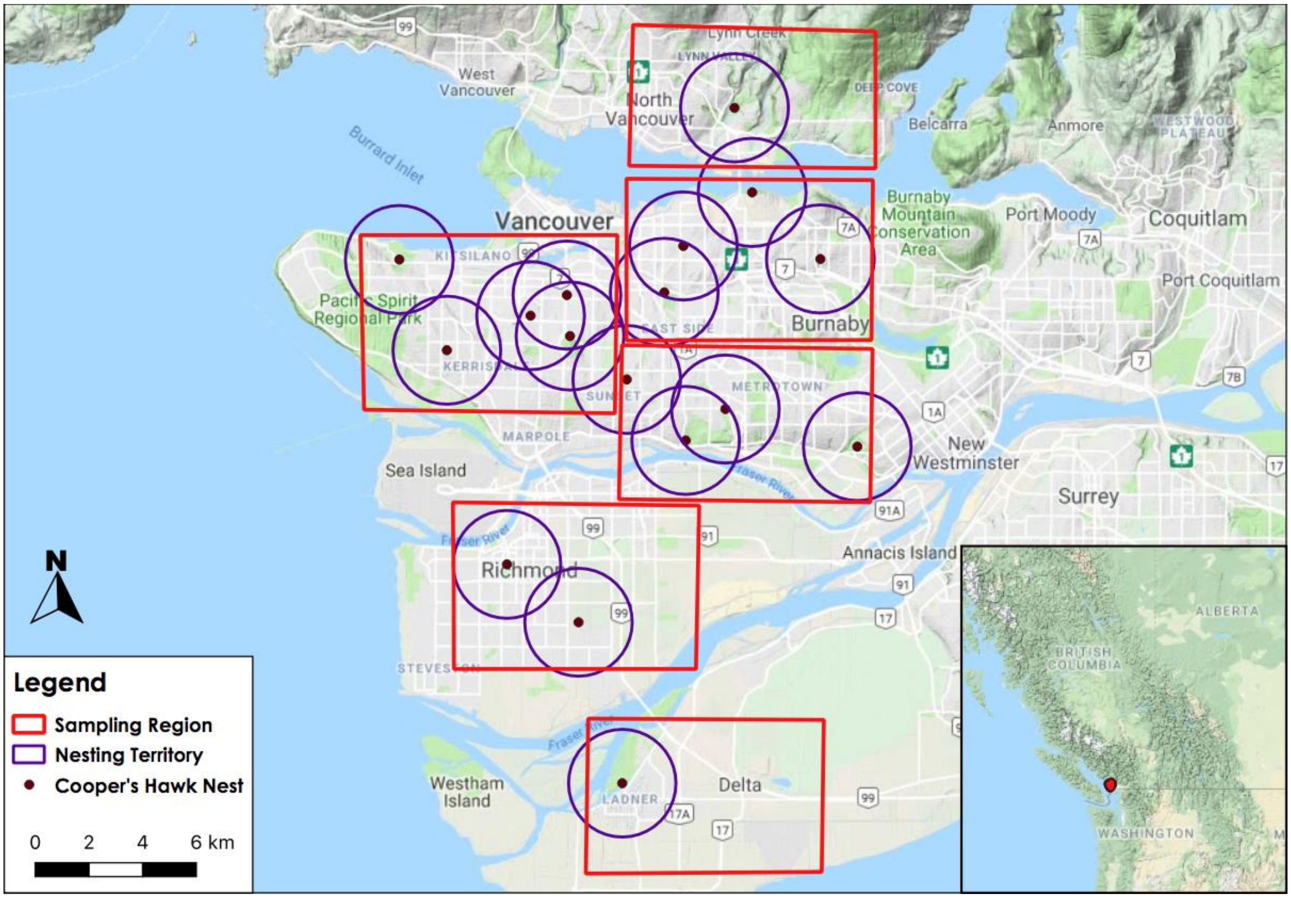

Figure S1. Study area separated into six sampling regions with Cooper's hawk nests and nesting territories in Metro Vancouver, British Columbia, 2016. Map created using the Free and Open Source QGIS.

Sample Collection

Air

Metal housings (TE-200-PAS, Tisch Environmental Inc., Cleves, OH, USA) with a 14 cm diameter x 1.3 cm thick polyurethane foam (PUF) disk (TE-104, Tisch Environmental Inc., Cleves, OH, USA) were installed at six locations (Figure S1). A bucket truck technician installed each sampler (n = 6) approximately 10 m off the ground onto the exposed trunk of a tree or onto a light standard within 125 – 900 m of a Cooper's hawk nest. A 10 m installation height roughly equates to a 1 km representative sampling radius or "footprint" (refer to the Supplementary Information of Fremlin, et al. [11] for further

sampling details). Air samplers were deployed for 90 days from September 8 to December 7, 2016, with an average daily air temperature of 11 ºC in Metro Vancouver.

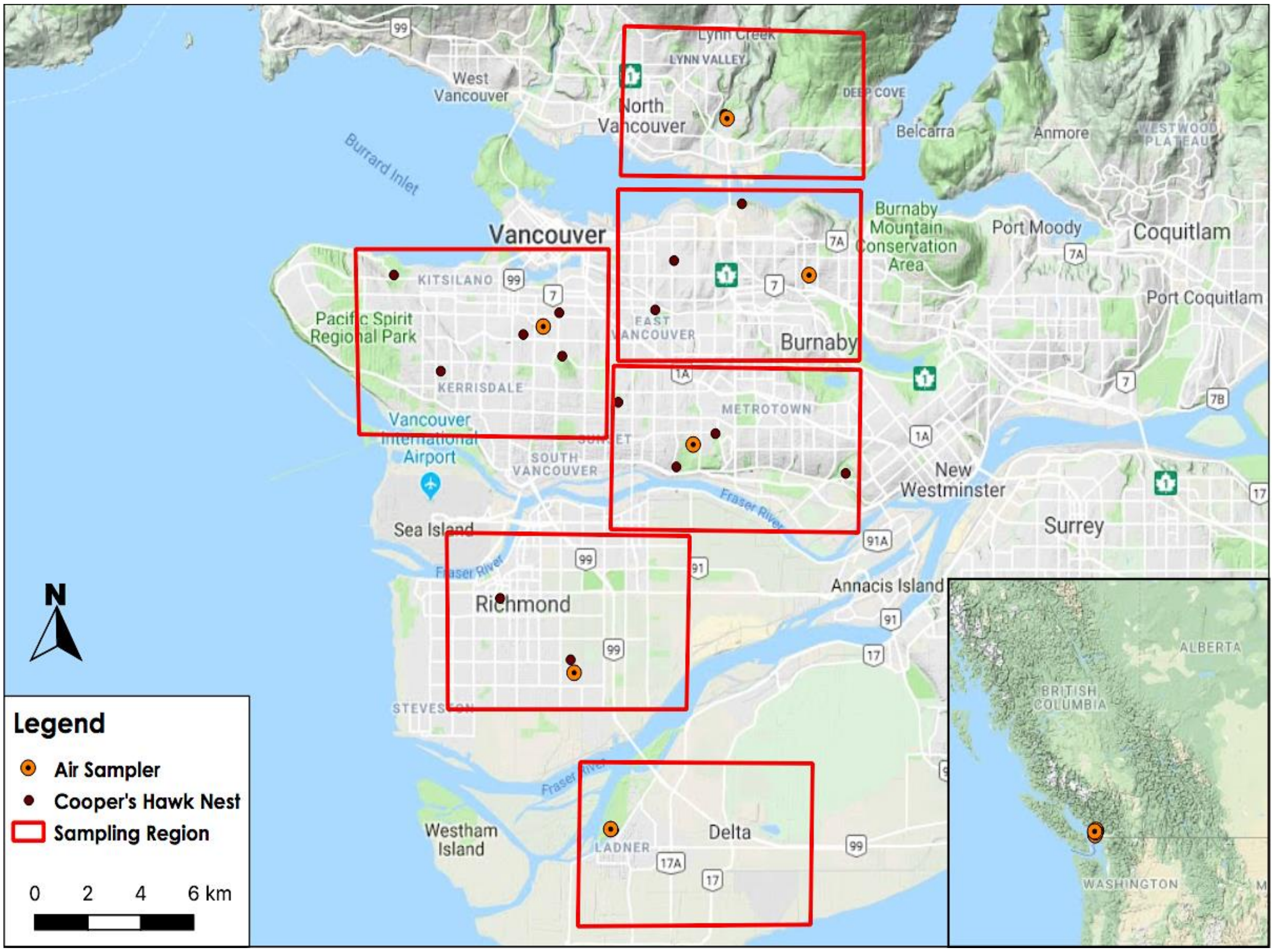

Figure S2. Locations of passive air samplers in relation to Cooper's hawk nest sites across Metro Vancouver, British Columbia, 2016. Map created using the Free and Open Source QGIS.

Soil

Within 5 m of an invertebrate or earthworm collection site, a soil sample (diameter approximately 10 cm with a depth of approximately 5 cm) was dug up with a metal trowel or a bulb planter and placed into a 250 ml chemically rinsed jar then frozen at –20°C. The trowel or bulb planter were cleaned with a 10% ethanol solution prior to digging at each subsampling station.

Biota

Cooper's hawk eggs were chosen to represent the apex predator because eggs represent a maternal concentration of contaminants at a given point in time and are frequently used as a matrix for environmental contamination monitoring (Figure S3).[21-23] The hawks' main target prey species were American Robins (*Turdus migratorius*), European Starlings (*Sturnus vulgaris*), and House Sparrows (*Passer domesticus*).[24] We supplemented our targeted songbird prey species with samples of 12 other known Cooper's Hawk prey species, [24-29] including Varied Thrush (*Ixoreus naevius*), Hermit Thrush (*Catharus guttatus*), Swainson's Thrush (*Catharus ustulatus*), Spotted Towhee (*Pipilo maculatus*), Song Sparrow (*Melospiza melodia*), White-crowned Sparrow (*Zonotrichia leucophrys*), Golden-crowned Sparrow (*Zonotrichia atricapilla*), Dark-eyed Junco (*Junco hyemalis*), Fox Sparrow (*Passerella iliaca*), Rock Pigeon (*Columba livia*), Eurasian-collared Dove (*Streptopelia decaocto*), and Northern Flicker (*Colaptes auratus*) that had been euthanized by a wildlife rehabilitation facility, Wildlife Rescue Association, Burnaby, BC. The lower levels of the food web were represented by terrestrial invertebrates (such as beetles, earthworms, and sowbugs) commonly eaten by birds (Figure S3).[30-35] Sample sizes of each species monitored in the current study are listed in Table S1.

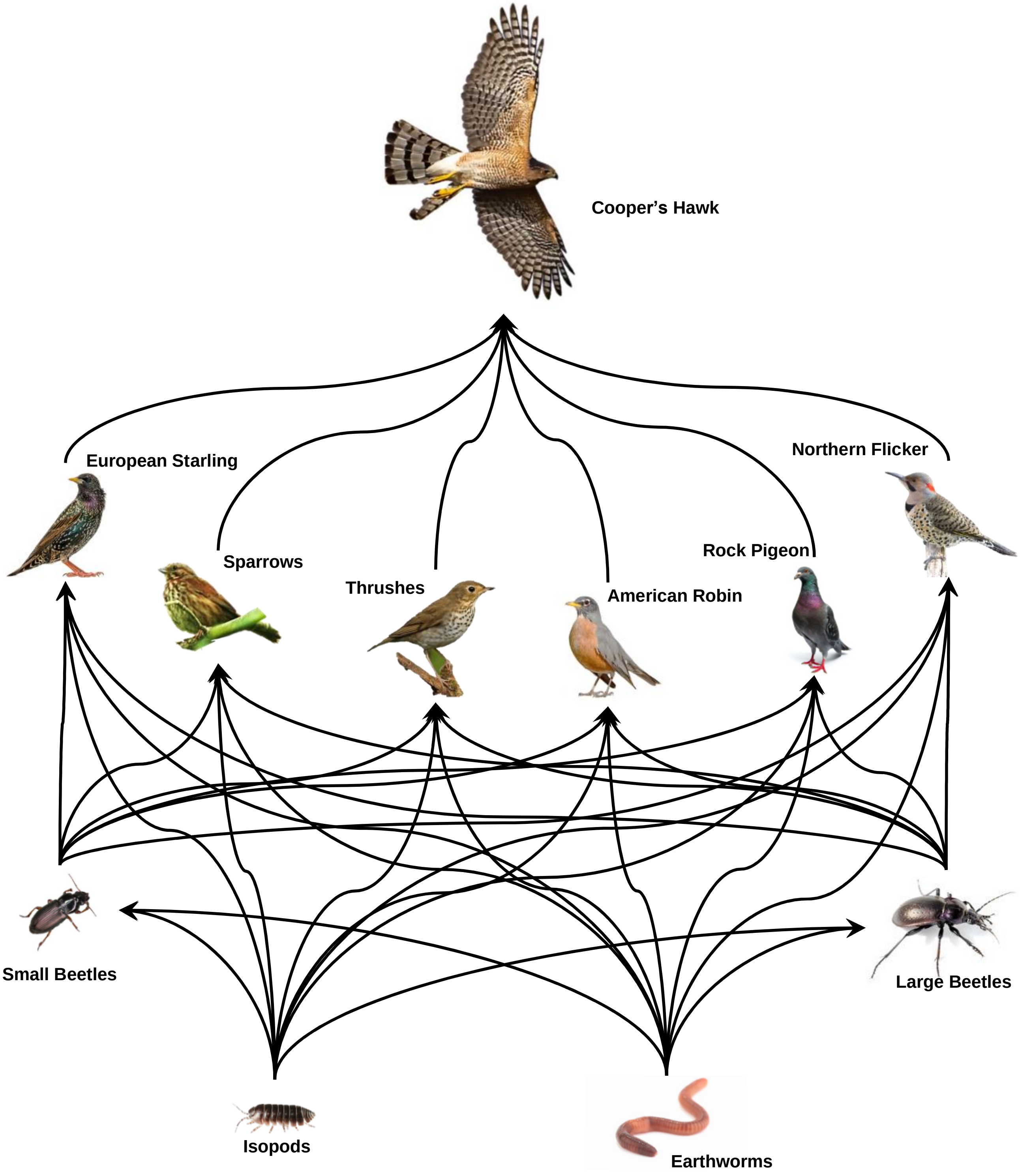

Figure S3. Urban terrestrial avian food-web with generalised trophic linkages among organisms collected in Metro Vancouver, BC in 2016.

Table S1. Species selected from the urban terrestrial food web in Fremlin, et al. [11] with the number of eggs (hawk) or composite samples analysed for PFAS in 2020.

Trophic Guild	Species	Scientific name	*n*
Apex Predator	Cooper's Hawk	*Accipiter cooperii*	12
Secondary Consumers	American Robin	*Turdus migratorius*	6
	European Starling	*Sturnus vulgaris*	5
	Northern Flicker	*Colaptes auratus*	6
	Rock Pigeon, Eurasian Collared Dove	*Columba livia, Streptopelia decaocto*	6
	**Sparrows:** House Sparrow, Dark-eyed Junco, White-crowned Sparrow, Fox Sparrow, Song Sparrow, Golden-crowned Sparrow, Spotted Towhee	*Passer domesticus, Junco hyemalis, Zonotrichia leucophrys, Passerella iliaca, Melospiza melodia, Zonotrichia atricapilla, Pipilo maculatus*	6
	**Thrushes:** Varied Thrush, Swainson's Thrush, Hermit Thrush	*Ixoreus naevius, Catharus ustulatus, Catharus guttatus*	6
Primary Consumers	Large Beetles	*Pterostichus melanrius, Carabus nemoralis, Carabus granulatus, Pterostichus sp.*	6
	Small Beetles	*Harpalus affinis, Calathus fuscipes, Anisodactylus binotatus, Agonum mülleri, Philonthus politus, Anatrichis minuta, Amara sp., Staphynlidae, Harpalitae*	5
Detritivores	Earthworms	*Lumbricidae*	12
	Oniscidea: Sowbugs and Pillbugs	*Oniscus asellus, Porcellio scaber, Armadillidium vulgare*	5

Sample Preparation

Air

PUF disks used for air sampling were precleaned and shipped as previously reported in Schuster, et al. [36]. PUF disks were precleaned using an Accelerated Solvent Extractor (ASE; ASE 350, Dionex Corporation, Sunnyvale, CA, USA) with one cycle of 200 mL of acetone and two cycles of 200 mL of hexane and dried under ultra-high-purity (UHP) grade nitrogen in a vacuum oven at 30 − 40 °C. PUF disks were stored in 1 L amber jars for shipping.

Biota

All frozen biota samples were shipped on dry ice to the National Wildlife Research Centre (NWRC) of Environment and Climate Change Canada (ECCC) in Ottawa, ON. Sample sizes of each species monitored in the current study are listed in Table S1. Cooper's hawk eggs were homogenized by whisking the yolk and albumen together. Songbirds were defeathered (i.e., plucked) and large keratinized or boney tissues (e.g., beaks, wings, legs, and feet) were removed prior to further processing. All frozen or semi-thawed biota samples (songbirds and invertebrates) were processed by cutting tissues into small pieces and homogenizing them with a ball-mill (Retsch^{TM} MM400 Mixer Mill, Fisher Scientific). Prior to and after homogenization, samples were stored at –40 °C.

Table S2. Perfluoroalkyl acids (PFAA) and corresponding internal standards that were included in the chemical analysis. Physicochemical properties were obtained from the CompTox Chemicals Dashboard for neutral molecules.[37, 38] Values are experimental averages (in black) or predicted averages (in red) from the Open (Quantitative) Structure-Activity/Property Relationship App (OPERA)*. MW = Molecular weight; VP = Vapour pressure; H = Henry's Law Constant; S_W = Water solubility; K_{OW} = Octanol-water partition coefficient.

Analyte	Acronym	Formula	Internal Standard	Carbon Chain Length	MW (g/mol)	VP* (Pa)	*H* (Pa m³/mol)	S_W (mol/L)	Log K_{OW}
			**Perfluorinated Carboxylic Acids (PFCA)**						
Perfluoro-n-butanoic acid	PFBA	$C_4HF_7O_2$	PFBA-$^{13}C_4$	4	214.0	21,864	5.08	2.09E-03	1.43
Perfluoro-n-pentanoic acid	PFPeA	$C_5HF_9O_2$	PFHxA-$^{13}C_2$	5	264.0	687.9	3.01E-05	4.54E-04	1.35
Perfluoro-n-hexanoic acid	PFHxA	$C_6HF_{11}O_2$	PFHxA-$^{13}C_2$	6	314.1	121.06	2.38E-05	9.34E-05	2.85
Perfluoro-n-heptanoic acid	PFHpA	$C_7HF_{13}O_2$	PFHxA-$^{13}C_2$	7	364.1	30.53	2.12E-05	0.324	2.05
Perfluoro-n-octanoic acid	PFOA	$C_8HF_{15}O_2$	PFOA-$^{13}C_4$	8	414.1	126.9	1.95E-05	1.00E-02	3.10
Perfluoro-n-nonanoic acid	PFNA	$C_9HF_{17}O_2$	PFNA-$^{13}C_5$	9	464.1	1.16	1.20E-04	2.80E-03	3.54
Perfluoro-n-decanoic acid	PFDA	$C_{10}HF_{19}O_2$	PFDA-$^{13}C_2$	10	514.1	0.20	1.52E-05	5.25E-03	4.15
Perfluoro-n-undecanoic acid	PFUdA	$C_{11}HF_{21}O_2$	PFUdA-$^{13}C_2$	11	564.1	0.09	3.38E-05	1.63E-04	4.00
Perfluoro-n-dodecanoic acid	PFDoA	$C_{12}HF_{23}O_2$	PFDoA-$^{13}C_2$	12	614.1	0.01	3.45E-05	1.35E-04	8.76
Perfluoro-n-tridecanoic acid	PFTrDA	$C_{13}HF_{25}O_2$	PFDoA-$^{13}C_2$	13	664.1	0.89	3.53E-05	4.29E-05	8.76
Perfluoro-n-tetradecanoic acid	PFTeDA	$C_{14}HF_{27}O_2$	PFDoA-$^{13}C_2$	14	714.1	0.28	3.60E-05	3.25E-05	5.10
Perfluoro-n-hexadecanoic acid	PFHxDA	$C_{16}HF_{31}O_2$	PFDoA-$^{13}C_2$	16	814.1	0.14	3.76E-05	2.63E-05	11.3
Perfluoro-n-octadecanoic acid	PFODA	$C_{18}HF_{35}O_2$	PFDoA-$^{13}C_2$	18	914.1	0.23	1.41E-05	1.25E-05	12.9
			**Perfluorinated Sulfonic Acids (PFSA)**						
Perfluorobutanesulfonic acid	PFBS	$C_4HF_9O_3S$	PFHxS-$^{18}O_2$	4	300.1	13.87	2.99E-05	1.70E-03	2.79
Perfluorohexanesulfonic acid	PFHxS	$C_6HF_{13}O_3S$	PFHxS-$^{18}O_2$	6	400.1	1.08E-06	1.97E-05	6.08E-04	2.20
Perfluorooctanesulfonic acid	PFOS	$C_8HF_{17}O_3S$	PFOS-$^{13}C_4$	8	500.1	3.31E-04	1.82E-06	1.36E-03	5.61
Perfluorodecanesulfonic acid	PFDS	$C_{10}HF_{21}O_3S$	PFHxS-$^{18}O_2$	10	600.1	1.09E-03	3.35E-05	3.23E-04	7.06
Perfluoro-4-ethylcyclohexane sulfonic acid	PFEtCHxS	$C_8HF_{15}O_3S$	PFHxS-$^{18}O_2$	8	462.1	1.45E-04	2.79E-03	1.10E-03	5.89

*VPs predicted from Open structure-activity/property Relationship App (OPERA) were considered the most accurate compared with other models, such as EPI Suite, COSMOtherm, and Linear Solvation Energy Relationships (LSERs). [39]

Air

Air samples were shipped to the Air Quality Processes Research Section of Environment and Climate Change Canada (ECCC), Toronto, ON, Canada and processed and analysed for poly-and perfluoroalkyl substances (PFAS) in the Hazardous Air Pollutants Laboratory (HAPs Lab) in 2017. Passive air sampling chemical analysis has been previously published in Shoeib, et al. [40] and Rauert, et al. [41]. Briefly, the extraction method consisted of spiking the PUF disks with surrogates of 0.5 ng of labelled PFAA (Table S2). PUFs were then extracted using ASE (ASE 350, Dionex Corporation, Sunnyvale, CA, USA) with acetonitrile (3 cycles) to extract the PFAAs. Extracted fractions were volume reduced to 0.5 mL using TurboVap (Biotage, Charlotte, NC, USA) and nitrogen blowdown. Each sample fraction was further purified with activated carbon columns containing 100 mg of ENVI-Carb (100–400 mesh, Supelco, St. Louis, MO) and eluted using 4 mL of acetonitrile before final solvent exchange to methanol and volume reduction to 0.5 mL. Prior to instrumental analysis, each sample was spiked with 0.5 ng of $^{13}C_8$ PFOA and $^{13}C_8$ PFOS for use as injection standards. Injection standards were used to quantify the surrogates and calculate surrogate recoveries. The separation and detection of PFAS was performed using ultra performance liquid chromatography tandem mass spectrometry (UPLC-MS/MS), following previously published methods in Shoeib, et al. [40].

Air concentrations (pg/m3 or ng/m3) of target analytes were derived from the mass of the chemical collected on the PUF divided by an effective air sampling volume. This air volume was determined with the GAPS template [42], using the average temperature during the deployment period and an estimated PUF-air partition coefficient from Ahrens, et al. [43]. The effective air volume was calculated as the number of days the PUF was deployed (i.e., 90) multiplied by a sampling rate of 4 m^3/day.[44]

Only one ion transition is monitored for PFBA and PFPeA, so there is a greater level of uncertainty in their reported concentrations compared to the other PFAS.[41]

Table S3. PFAA chemicals with names, abbreviations, associated surrogate/injection standard, and quantifier transition used in processing and analysis of PUF disks.

Compound	Acronym	Allocation of surrogate/ injection standard	Quantifier transition (m/z)
^{13}C Perfluorobutanoic acid	^{13}C PFBA	$^{13}C_8$ PFOA	217 > 172
$^{18}O_6$ Perfluorohexanoic acid	$^{13}C_2$ PFHxA	$^{13}C_8$ PFOA	315 > 270
$^{13}C_4$ Perfluorooctanoic acid	$^{13}C_4$ PFOA	$^{13}C_8$ PFOA	417 > 372
$^{13}C_5$ Perfluorononanoic acid	$^{13}C_5$ PFNA	$^{13}C_8$ PFOA	468 > 423
$^{13}C_2$ Perfluorodecanoic acid	$^{13}C_2$ PFDA	$^{13}C_8$ PFOA	515 > 470
$^{13}C_2$ Perfluoroundecanoic acid	$^{13}C_2$ PFUnDA	$^{13}C_8$ PFOA	565 > 520
$^{13}C_2$ Perfluorododecanoic acid	$^{13}C_2$ PFDoDA	$^{13}C_8$ PFOA	615 > 570

Compound	Acronym	Allocation of surrogate/ injection standard	Quantifier transition (m/z)
$^{18}O_2$ Perfluorohexane sulfonate	$^{18}O_2$ PFHxS	$^{13}C_8$ PFOS	403 > 103
$^{13}C_4$ Perfluorooctane sulfonate	$^{13}C_4$ PFOS	$^{13}C_8$ PFOS	503 > 80
$^{13}C_8$ Perfluorooctanoic acid	$^{13}C_8$ PFOA	-	421 > 376
$^{13}C_8$ Perfluorooctane sulfonate	$^{13}C_8$ PFOS	-	507 > 80

Soil

Processing and analysis of soil samples was completed at SGS AXYS Analytical Services Ltd. in Sidney, BC in 2020. Approximately 5 g dry weight of each soil sample was spiked with isotopically labelled surrogate standards and extracted by shaking three times with methanolic ammonium hydroxide solution and collecting the supernatants each time. The supernatants are combined, treated with ultra-pure carbon powder, and evaporated to remove methanol. The resulting solution is diluted with water and cleaned up by solid phase extraction (SPE) using disposable cartridges containing a weak anion exchange sorbent. The eluate is spiked with recovery standards and analysed by LC-MS/MS.

Biota

After homogenization, hawk eggs, avian prey, and invertebrates were analysed for PFAS at NWRC in 2020. Sample extraction methods used at NWRC have been described in detail in comparative studies on PFAS within eggs of seabirds in Braune and Letcher [45]. Briefly, approximately 0.01 – 0.3 g of sample homogenate was spiked with 100 µL of the internal standard solution mixture with a concentration of 100 ng/mL (with exception to PFOS which was 1000 ng/mL) of each labelled compound and extracted with 3 mL of formic acid acetonitrile/water (0.2%) solution. After extraction, 0.04 to 0.05 g of active carbon was added to the extract and diluted with water, and the target compounds were enriched and fractioned on a Waters Oasis weak anion exchange (WAX) solid phase extraction (SPE) cartridge (60 mg × 3 mL). The SPE WAX cartridges were washed with 1 mL of 2 % formic acid in aqueous solution, 2 × 1 mL of water, and 1 mL of methanol. PFAAs (including PFCAs and PFSAs) were eluted with 2 × 1 mL 1 % ammonium hydroxide solution/methanol (1v/99v). The separation of the target compounds in each sample was determined on a Xevos TQ-S Ultra performance liquid chromatograph–mass spectrometer system (UPLC-MS/MS; Waters, Mississauga, ON, Canada). The data was processed using MassLynx software (version 4.1). The UPLC-MS/MS was equipped with a Kinetex™ UPLC® C18 analytical column (1.7 µm particle size, 2.1 × 50 mm; Waters, ON, Canada) and injected with 10 µL of extracted sample. The column temperature was held at 50°C while the sample was maintained at 20 °C. The PFAAs were

detected by negative electrospray ionization (ESI-) in multiple reaction monitoring scanning mode (MRM). The relative response of a given analyte to its mass-labelled internal standard is used to calculate the concentration of the analyte, which corrects for any experimental losses of both the analyte and its internal standard.

Standards and Chemicals

All the PFSA/PFCA standards (including isotopically enriched internal standards in Table S3) were purchased from Wellington Laboratories (Guelph, ON, Canada), except for perfluoro-4-ethylcyclohexane sulfonic acid potassium salt (PFEtCHxS), which was purchased from Campro Scientific GmbH (Berlin, Germany). Their molecular structures can be found in the *Chemical Analysis* section. Methanol used for UPLC-MS/MS is Ultra CHROMASOLV grade (UPLC grade) from Canadian Life Science. All other solvents and reagents used in the process are HPLC grade or better.

Quality Control and Assurance

Air

During the installation of the air samples, three field blanks were deployed and collected (i.e., opened, placed in the metal housing, removed, and sealed as a negative control) at three sites: Inter River Park, North Vancouver; Heather St. and W. 20th Ave, Vancouver – West.; and Ladner Harbour Park, Delta. During the air sample processing, two procedural sample blanks were run with separate sets of 4 – 5 samples each (Table S4). Each sample was spiked with surrogate standards. The limit of detection (LOD) for each contaminant in the air was determined as 2xSD of the mean concentration of the field and procedural blanks (Table S5). The method detection limit (MDL) for each contaminant in the air was determined as the mean concentration of the field and procedural blanks + 2×SD (Table S5). Data below the MDL were reported as non-detect (ND). Air samples were recovery and blank corrected using the MDL of the field and procedural blank concentrations.

Soil

To check for contamination of analytes from solvents or the extraction process, one procedural blank, one spiked matrix, and one duplicate was prepared and analysed for the batch of 12 soil samples. Initial calibration of the LC-MS/MS instrument is performed by the analysis of five or more calibration

solutions and the lowest calibration standard is used as the reporting limit (RL; Table S6). A mid-level calibration standard is analysed to verify the initial calibration and injected after every 12 hours or after every 10 client samples (whichever comes first). Sample specific detection limits (SDL) are determined by converting the area equivalent to 3 times the estimated chromatographic noise height to a concentration in the same manner that target peak responses are converted to final concentrations (Table S6). The SDL accounts for any effect of matrix on the detection system and for recovery achieved through the analytical work-up. For some samples, the percent recoveries for some surrogate compounds did not meet the method criteria limits. But as the isotope dilution method of quantification produces data that is recovery corrected, these variances from method criteria were deemed to not affect the quantification of the target analytes. Percent surrogate recoveries are used as general method performance indicator only; percent recoveries for PFAS in the spiked matrix ranged from 73 – 98 % (Table S6). Soil samples were blank corrected with the procedural blank.

Biota

To check for contamination of analytes from solvents or the extraction process, a blank sample was prepared and analysed for each batch of 10 – 11 samples (n = 8; Table S4). In-house reference materials, a double-crested cormorant (*Phalacrocorax auratus*) egg (DCCO 03L86S01, Environment Canada, National Wildlife Specimen Bank; n = 1) was analysed concurrently with the Cooper's hawk egg samples and Lake Michigan Fish Tissue (NIST 1947 Lake Michigan Fish Tissue; n = 7), were analysed concurrently with the songbird and invertebrate samples. Average internal standard recoveries (%) for PFBA-$^{13}C_4$, PFHxA-$^{13}C_2$, PFOA-$^{13}C_4$, PFNA-$^{13}C_5$, PFDA-$^{13}C_2$, PFUdA-$^{13}C_2$, PFDoA-$^{13}C_2$, PFHxS-$^{18}O_2$, and PFOS-$^{13}C_4$ in the songbirds, invertebrates, blanks, and Lake Michigan Fish Tissue samples were 117 (34 SD), 67 (24 SD), 65 21 SD), 66 (26 SD), 71 (17 SD), 66 (17 SD), 56 (15 SD), 78 (15 SD), and 78 (16 SD), respectively. Average internal standard recoveries (%) for PFBA-$^{13}C_4$, PFHxA-$^{13}C_2$, PFOA-$^{13}C_4$, PFNA-$^{13}C_5$, PFDA-$^{13}C_2$, PFUdA-$^{13}C_2$, PFDoA-$^{13}C_2$, PFHxS-$^{18}O_2$, and PFOS-$^{13}C_4$ in the Cooper's hawk eggs, blank, and DCCO egg sample were 79 (32 SD), 62 (36 SD), 61 (34 SD), 59 (34 SD), 61 (35 SD), 60 (33 SD), 58 (29 SD), 75 (13 SD), and 76 (11 SD), respectively.

The method limit of quantification (MLOQ) was measured by performing replicate analyses of blank matrix samples (n=8) spiked with analytes at a concentration of 3 – 5 times the estimated detection limit and calculating the standard deviation: MLOQ = t × SD (Table S5). Where t = Student's value for a 99 % confidence interval and a standard deviation with n-1 degree of freedom, and SD = standard deviation of

the replicate control. In this experiment $t_{(n-1, 0.99)} = 2.998$. MLOQ were determined for samples of hawk eggs, songbirds, and invertebrates (Table S5).

The method limit of detection (MLOD) was determined separately for samples of hawk eggs, birds, and invertebrates (Table S5). MLODs were defined as the concentration that would give a signal to noise ratio (peak to peak) of 3, which means there is no detected peak (or a signal to noise ratio [peak to peak] lower than 3) of the target compound found in the respective ion extracted chromatogram within its retention time window. PFAS concentrations reported are corrected for background contamination by subtracting their respective method blank concentration values.

Table S4. Mean, Min, and Max concentrations of PFAS in field and/or procedural blanks for air and biota samples.

	Air Blanks (pg/m^3)			Biota Blanks (ng/g ww)		
**PFAS**	**Mean**	**Min**	**Max**	**Mean**	**Min**	**Max**
PFBA	7.772	0.301	127	5.1301	0.8541	17.6347
PFPeA	2.922	0.706	48.7	0.0427	0.0108	0.0745
PFHxA	0.746	0.093	19.3	0.0275	0.0114	0.0475
PFHpA	0.511	0.160	8.553	0.0161	0.0048	0.0347
PFOA	0.368	0.113	5.536	0.0670	0.0434	0.1074
PFNA	0.129	0.018	2.051	0.0138	0.0045	0.0305
PFDA	0.075	0.014	0.754	0.0133	0.0077	0.0297
PFUdA	0.184	0.023	0.464	0.0286	0.0186	0.0451
PFDoA	0.048	0.006	0.141	0.0095	0.0038	0.0172
PFTrDA	0.015	0.001	0.032	0.0089	0.0031	0.0148
PFTeDA	0.014	0.010	0.019	0.0098	0.0058	0.0166
PFHxDA	0.011	0.004	0.016	0.0258	0.0101	0.0381
PFODA	0.013	0.002	0.044	0.0108	0.0015	0.0227
PFBS	0.029	0.006	0.071	0.0327	0.0077	0.0492
PFHxS	0.007	0.001	0.013	0.0367	0.0208	0.0483
PFOS	0.052	0.001	0.107	0.0125	0.0026	0.0243
PFDS	0.014	0.004	0.025	0.0027	0.0013	0.0040
PFEtCHxS	NA	NA	NA	0.0012	0.0001	0.0043

Table S5. Limit of detection (LOD) and method detection limit (MDL) of PFAS within air samples at HAPs Lab determined with average air volume (m^3) of six samples. Method limit of detection (MLOD) and method limit of quantification (MLOQ) of PFAS within respective biota samples at NWRC. NA = Not analysed.

	Air (pg/m^3)		Hawk Egg (ng/g ww)		Songbird (ng/g ww)		Invertebrate (ng/g ww)	
PFAS	LOD	MDL	MLOD	MLOQ	MLOD	MLOQ	MLOD	MLOQ
PFBA	29.0	36.8	0.063	0.075	0.8	2.6	0.075	0.099
PFPeA	6.77	9.69	0.007	0.03	0.03	0.1	0.06	0.18
PFHxA	2.32	3.07	0.01	0.03	0.05	0.17	0.3	1
PFHpA	1.19	1.70	0.03	0.1	0.1	0.3	0.03	0.1
PFOA	0.652	1.02	0.02	0.07	0.03	0.1	0.06	0.2
PFNA	0.370	0.499	0.03	0.1	0.03	0.09	0.02	0.07
PFDA	0.139	0.214	0.1	0.3	0.009	0.03	0.02	0.07
PFUnDA	0.375	0.559	0.1	0.3	0.02	0.06	0.008	0.02
PFDoDA	0.107	0.155	0.03	0.1	0.01	0.03	0.02	0.07
PFTrDA	0.0313	0.0461	0.02	0.06	0.009	0.03	0.01	0.03
PFTeDA	0.00734	0.0211	0.008	0.03	0.01	0.04	0.02	0.07
PFHxDA	0.0106	0.0219	0.1	0.4	0.01	0.04	0.02	0.06
PFODA	0.0355	0.0481	0.02	0.07	0.1	0.3	0.02	0.07
PFBS	0.0519	0.0805	0.01	0.03	0.006	0.02	0.07	0.22
PFHxS	0.00977	0.0165	0.1	0.4	0.02	0.06	0.06	0.2
PFOS	0.0819	0.133	0.2	0.7	0.1	0.3	0.2	0.7
PFDS	0.0294	0.0434	0.07	0.2	0.03	0.1	0.09	0.3
PFEtCHxS	NA	NA	0.03	0.1	0.03	0.1	0.002	0.006

Table S6. Reporting limits (RL) of PFAS based on the lowest calibration standard analysed in a 5 g dw soil sampl); % recovery in spiked matrix sample; and sample specific detection limits (SDL) of PFAS in the two soil samples from each sampling region corrected for sample specific weight. NVAN = North Vancouver; NBBY = North Burnaby – East Vancouver; VANW = Vancouver – West; VANS = Vancouver – South; DELT = Delta; RICH = Richmond. RL also equal to concentration in lab blank (*n*=1).

	RL		NVAN (ng/g dw)		NBBY (ng/g dw)		VANW (ng/g dw)		VANS (ng/g dw)		DELT (ng/g dw)		RICH (ng/g dw)	
PFAS	(ng/g dw)	% Recovery	SDL 1	SDL 2	SDL 1	SDL 2	SDL 1	SDL 2	SDL 1	SDL 2	SDL 1	SDL 2	SDL 1	SDL 2
PFBA	0.320	94	0.2840	0.3250	0.2910	0.3033	0.2985	0.2879	0.3124	0.2873	0.2877	0.3149	0.2927	0.5461
PFPeA	0.160	93	0.1420	0.1625	0.1455	0.1517	0.1492	0.1439	0.1562	0.1437	0.1438	0.1574	0.1464	0.2730
PFHxA	0.080	92	0.0710	0.0813	0.0728	0.0758	0.0746	0.0720	0.0781	0.0718	0.0719	0.0787	0.0732	0.1365
PFHpA	0.080	92	0.0710	0.0813	0.0728	0.0758	0.0746	0.0720	0.0781	0.0718	0.0719	0.0787	0.0732	0.1365
PFOA	0.080	85	0.0710	0.0813	0.0728	0.0758	0.0746	0.0720	0.0781	0.0718	0.0719	0.0787	0.0732	0.1365
PFNA	0.080	97	0.0710	0.0813	0.0728	0.0758	0.0746	0.0720	0.0781	0.0718	0.0719	0.0787	0.0732	0.1365
PFDA	0.080	73	0.0710	0.0813	0.0728	0.0758	0.0746	0.0720	0.0781	0.0718	0.0719	0.0787	0.0732	0.1365
PFUdA	0.080	98	0.0710	0.0813	0.0728	0.0758	0.0746	0.0720	0.0781	0.0718	0.0719	0.0787	0.0732	0.1365
PFDoA	0.080	91	0.0710	0.0813	0.0728	0.0758	0.0746	0.0720	0.0781	0.0718	0.0719	0.0787	0.0732	0.1365
PFTrDA	0.080	92	0.0710	0.0813	0.0728	0.0758	0.0746	0.0720	0.0781	0.0718	0.0719	0.0787	0.0732	0.1365
PFTeDA	0.080	94	0.0710	0.0813	0.0728	0.0758	0.0746	0.0720	0.0781	0.0718	0.0719	0.0787	0.0732	0.1365
PFBS	0.080	98	0.0710	0.0813	0.0728	0.0758	0.0746	0.0720	0.0781	0.0718	0.0719	0.0787	0.0732	0.1365
PFPeS	0.080	92	0.0710	0.0813	0.0728	0.0758	0.0746	0.0720	0.0781	0.0718	0.0719	0.0787	0.0732	0.1365
PFHxS	0.080	91	0.0710	0.0813	0.0728	0.0758	0.0746	0.0720	0.0781	0.0718	0.0719	0.0787	0.0732	0.1365
PFHpS	0.080	91	0.0710	0.0813	0.0728	0.0758	0.0746	0.0720	0.0781	0.0718	0.0719	0.0787	0.0732	0.1365
PFOS	0.080	83	0.0710	0.0813	0.0728	0.0758	0.0746	0.0720	0.0781	0.0718	0.0719	0.0787	0.0732	0.1365
PFNS	0.080	80	0.0710	0.0813	0.0728	0.0758	0.0746	0.0720	0.0781	0.0718	0.0719	0.0787	0.0732	0.1365
PFDS	0.080	88	0.0710	0.0813	0.0728	0.0758	0.0746	0.0720	0.0781	0.0718	0.0719	0.0787	0.0732	0.1365
PFDOS	0.080	86	0.0710	0.0813	0.0728	0.0758	0.0746	0.0720	0.0781	0.0718	0.0719	0.0787	0.0732	0.1365

Tissue Composition

Lipid Composition

During the chemical analysis for legacy hydrophobic contaminants in Fremlin, et al. [11], approximately 0.25 – 3.0 g of biota sample homogenate was ground with diatomaceous earth (J.T. Baker, NJ, U.S.A.), spiked with 25 μL of a standard solution, and then extracted with a 50:50 dichloromethane:hexane (DCM:HEX) solvent mixture using an accelerated solvent extraction system (ASE, Dionex ASE 350, CA, USA). Approximately 10% of this extract was subsequently used in the gravimetric determination of total lipid content.

To determine the fraction of polar lipids in each sample, we used a Phospholipid Assay Kit (MAK122, MilliporeSigma, Sigma-Aldrich Corp., Oakville, ONT), which measures the amount of choline released by phospholipids when enzymatically hydrolyzed with choline oxidase. Approximately 10 – 20 μL of tissue homogenate was diluted with 90 – 490 μL of 0.5% Triton™ X-100 (X100-5ML, MilliporeSigma, Sigma-Aldrich Corp., Oakville, ONT), then 20 μL of each sample was transferred in duplicate into a 96 well plate. Standards for colorimetric detection were prepared from a 200 μM working solution by first adding 24 μL of the 2mM Phosphatidylcholine Standard to 216 μL of deionized water; then five standards with concentrations of 0, 30, 60, 120, and 200 μM were prepared by adding 0, 15, 30, 60, and 100 μL of the 200 μM working solution to deionized water for a total volume of 100 μL each. Next, 20 μL of each standard was transferred in duplicate into a 96 well plate. Then, 80 μL of a prepared reaction mix (85 μL assay buffer, 1 μL enzyme mix, 1 μL PLD enzyme, and 1 μL dye reagent) was added to each well; and the well plate was wrapped in aluminum foil and incubated at room temperature for 30 mins. Finally, the absorbance of the samples and standards was measured at 570 nm in a microplate reader (SpectraMax® M2e, Molecular Devices, LLC, San Jose, CA). The fraction of non-polar lipids in each sample was then derived by subtracting the fraction of polar lipids from the fraction of total lipids.

Protein Composition

To determine the fractions of total protein, albumin, and structural protein in the samples, we measured the amount of total protein in each sample with a Bradford assay method,[46] and subsequently derived the fractions of albumin and structural protein from the total protein.

For the Bradford assay, approximately 0.1 – 0.3 mL of sample homogenate was diluted with 0.1 M KPO_4 buffer (pH 7.4) with dilution factors ranging from 80 – 375. Six standards for colorimetric detection were prepared from a 0.2 mg/mL Bovine Serum Albumin (BSA A2153, MilliporeSigma, Sigma-Aldrich

Corp., Oakville, ONT) working solution with concentrations of 0, 10, 20, 40, 60, 80 μg/mL of BSA in 0.1 M KPO_4 buffer. Then, 50 μL of each standard and each sample was transferred in triplicate and quintuplicate, respectively, to a 96 well plate. Next, 200 μL of 1/5 diluted Bio-Rad Protein Assay Dye Reagent (500-0006, Bio-Rad Laboratories Ltd., Montreal, QC) in deionized water was transferred to each standard and sample well; and the well plate was wrapped in aluminum foil and incubated at room temperature for 5-10 mins. Finally, the absorbance of the samples and standards was measured at 595 nm in a microplate reader (SpectraMax® M2e, Molecular Devices, LLC, San Jose, CA). Organic matter (e.g., fiber and carbohydrates) in each sample was estimated as the dry weight (i.e., 100 – water content %) minus the total protein and total lipid content.

Apparent Chemical Activity

We obtained measured distribution coefficients for PFAS from Allendorf, et al. [16], Droge [18], and Bischel, et al. [17] (Table S7). We used the distribution coefficients measured by Allendorf, et al. [16] for our chemical activity calculations and modelling instead of those from Bischel, et al. [17] and Droge [18] because (i) the distribution coefficients reported in these two studies were determined at 21 – 25°C, which are not environmentally relevant body temperatures for birds [47-49] or preferred optimal temperatures for terrestrial invertebrates like earthworms,[50-52] and (ii) since Allendorf, et al. [16] reported distribution coefficients for different tissue types this maintains consistency across experimental conditions.

For PFAS that did not have measured tissue-water distribution coefficients, we predicted the respective distribution coefficients from linear regressions using all the measured values from Allendorf, et al. [16], Droge [18], and Bischel, et al. [17] and the molar volume of PFAS (Table S8). Molar volume was chosen rather than total surface area or carbon chain length to predict distribution coefficients since several quantitative structure property relationships based on molar volume of PFAS were often statistically better than those based on total surface area and can help distinguish between PFAS with the same number of attached fluorines and/or carbon chain length [53]. Also, carbon chain length, molar volume, molar weight, and the number of attached fluorine all appeared to have similar linear relationships with the measured distribution coefficients (Figure S4). We derived separate linear regressions for PFCA and PFSA, which were all statistically significant ($p < 0.01$) and demonstrated reasonably good fits (Figure S5 and S6) apart from the storage lipid-water distribution coefficient (D_{SLW}) for PFSA ($p > 0.05$). The relationship for D_{SLW} of PFSA had a very large confidence interval likely due to the small sample size in the model, which adds greater uncertainty to subsequent analyses relying on these predictive values.

Table S7. Measured tissue-water distribution coefficients of PFAS obtained from Allendorf, et al. [16], Bischel, et al. [17], and Droge [18] used to predict distribution coefficients of PFAS that were not measured or analysed (i.e., NA). Chain = carbon chain length; F_N = number of attached fluorine; V_M = molar volume (cm^3/mol); MW = molar weight (g/mol); D_{MLW} = Membrane or polar lipid-water; D_{ALBW} = Albumin-water; D_{SPW} = Structural protein-water; D_{SLW} = storage or neutral lipid-water. Values for V_M and MW were obtained from the CompTox Chemicals Dashboard for neutral molecules. [37, 38]

					Allendorf, et al. [16]	Allendorf, et al. [16]	Allendorf, et al. [16]	Allendorf, et al. [16]	Bischel, et al. [17]	Droge [18]
PFAS	Chain	F_N	V_M	MW	Log D_{MLW} (L/L)	Log D_{ALBW} (L/L)	Log D_{SPW} (L/L)	Log D_{SLW} (L/L)	Log D_{ALBW} (L/L)	Log D_{MLW} (L/L)
PFBA	3	7	128	214	1.70	2.65	NA	-1.40	NA	1.00
PFPeA	4	9	155	264	NA	NA	NA	NA	3.4	1.73
PFHxA	5	11	182	314	2.32	3.56	0.64	-1.50	4.05	2.31
PFHpA	6	13	210	364	2.91	4.16	1.3	-1.10	4.23	2.87
PFOA	8	15	237	414	3.52	4.33	1.61	-1.37	4.14	3.51
PFNA	9	17	265	464	4.25	4.46	2.17	-0.73	4.05	4.04
PFDA	10	19	292	514	4.82	4.86	2.96	-0.55	3.86	4.63
PFUdA	11	21	315	564	4.54	4.74	3.42	-0.32	3.70	NA
PFDoA	12	23	347	614	NA	NA	NA	NA	3.30	NA
PFTrDA	13	25	375	664	NA	NA	NA	NA	NA	NA
PFTeDA	14	27	397	714	NA	NA	NA	NA	NA	NA
PFHxDA	16	31	457	814	NA	NA	NA	NA	NA	NA
PFBS	4	9	162	300.0	2.86	3.34	0.74	NA	3.86	2.63
PFHxS	6	13	217	400.1	4.13	4.94	1.73	-1.58	4.30	3.82
PFOS	8	17	272	500.1	4.89	4.81	2.94	-0.56	4.10	4.88
PFDS	10	21	327	600.1	NA	NA	NA	NA	NA	NA
PFECHS	8	15	239	500	4.53	4.68	2.57	-1.09	NA	NA
9Cl-PF3ONS	8	16	284	570.7	5.14	5.14	3.49	-0.86	NA	NA

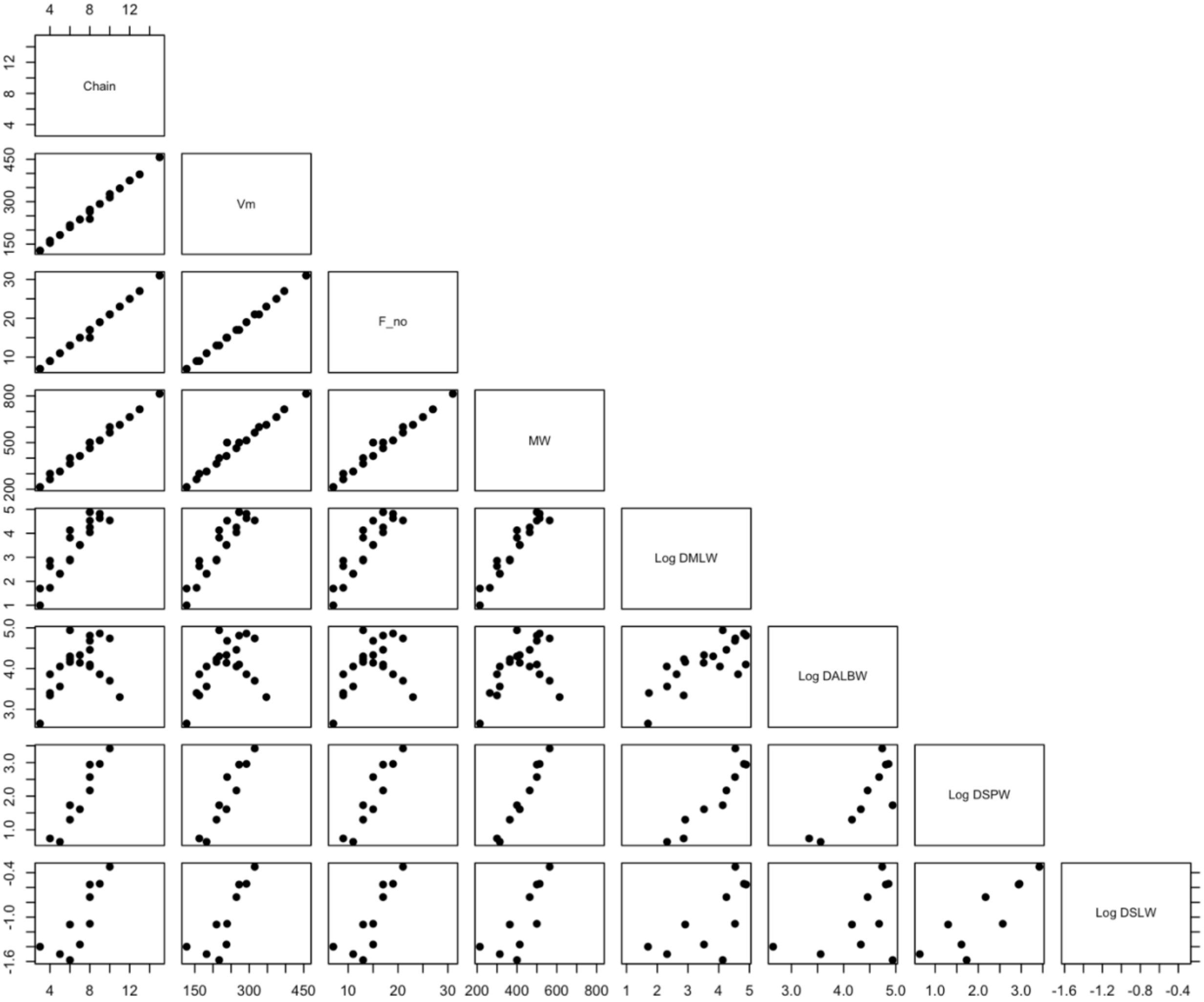

Figure S4. Scatterplot illustrating relationships between the physical properties and measured distribution coefficients of PFAS. Chain = carbon chain length, Vm = molar volume (cm^3/mol); F_no = number of attached fluorine; MW = molar weight (g/mol); Log DMLW = Membrane or polar lipid-water (L/L); Log DALBW = Albumin-water (L/L); Log DSPW = Structural protein-water (L/L); and Log DSLW = storage or neutral lipid-water (L/L).

Table S8. Measured tissue-water distribution coefficients of PFAS from Allendorf, et al. [16] and predicted values used to determine apparent chemical activities. V_M = molar volume; D_{MLW} = Membrane or polar lipid-water; D_{ALBW} = Albumin-water; D_{SPW} = Structural protein-water; D_{SLW} = storage or neutral lipid-water.

PFAS	V_M (cm^3/mol)	Log D_{MLW} (L/L)	Log D_{ALBW} (L/L)	Log D_{SPW} (L/L)	Log D_{SLW} (L/L)
PFOA	237	3.52	4.33	1.61	-1.37
PFNA	265	4.25	4.46	2.17	-0.73
PFDA	292	4.82	4.86	2.96	-0.55
PFUdA	315	4.54	4.74	3.42	-0.32
PFDoA	347	5.63[a]	3.55[b]	4.02[c]	-0.28[d]

PFAS	V_M (cm³/mol)	Log D_{MLW} (L/L)	Log D_{ALBW} (L/L)	Log D_{SPW} (L/L)	Log D_{SLW} (L/L)
PFTrDA	375	6.18[a]	2.96[b]	4.60[c]	-0.11[d]
PFTeDA	397	6.61[a]	2.39[b]	5.05[c]	0.03[d]
PFHxDA	457	7.80[a]	0.29[b]	6.30[c]	0.41[d]
PFBS	162	2.86	3.34	0.74	-2.15[d]
PFHxS	217	4.13	4.94	1.73	-1.58
PFOS	272	4.89	4.81	2.94	-0.56
PFDS	327	6.04[a]	5.32[b]	4.32[c]	-0.11[d]

[a]Predicted with experimental values from Allendorf, et al.[16] and Droge[18] using equations: PFCA Log $D_{MLW} = 0.0197 \times V_M - 1.21$, $r^2 = 0.96$, $p < 0.001$; PFSA Log $D_{MLW} = 0.0195 \times V_M - 0.351$, $r^2 = 0.97$, $p < 0.001$.
[b]Predicted with experimental values from Allendorf, et al.[16] and Bischel, et al.[17] using equations: PFCA Log $D_{ALBW} = 0.0525 \times V_M - 0.000102 \times V_M^2 - 2.37$, $r^2 = 0.63$, $p < 0.001$; PFSA Log $D_{ALBW} = 0.00935 \times V_M + 2.26$, $r^2 = 0.46$, $p = 0.038$.
[c]Predicted with experimental values from Allendorf, et al.[16] using equations: PFCA Log $D_{SPW} = 0.0207 \times V_M - 3.61$, $r^2 = 0.99$, $p < 0.001$; PFSA Log $D_{SPW} = 0.0220 \times V_M - 2.87$, $r^2 = 0.97$, $p = 0.0016$.
[d]Predicted with experimental values from Allendorf, et al.[16] using equations: PFCA Log $D_{SLW} = 0.00625 \times V_M - 2.45$, $r^2 = 0.72$, $p = 0.0096$; PFSA Log $D_{SLW} = 0.0124 \times V_M - 4.16$, $r^2 = 0.66$, $p = 0.12$.

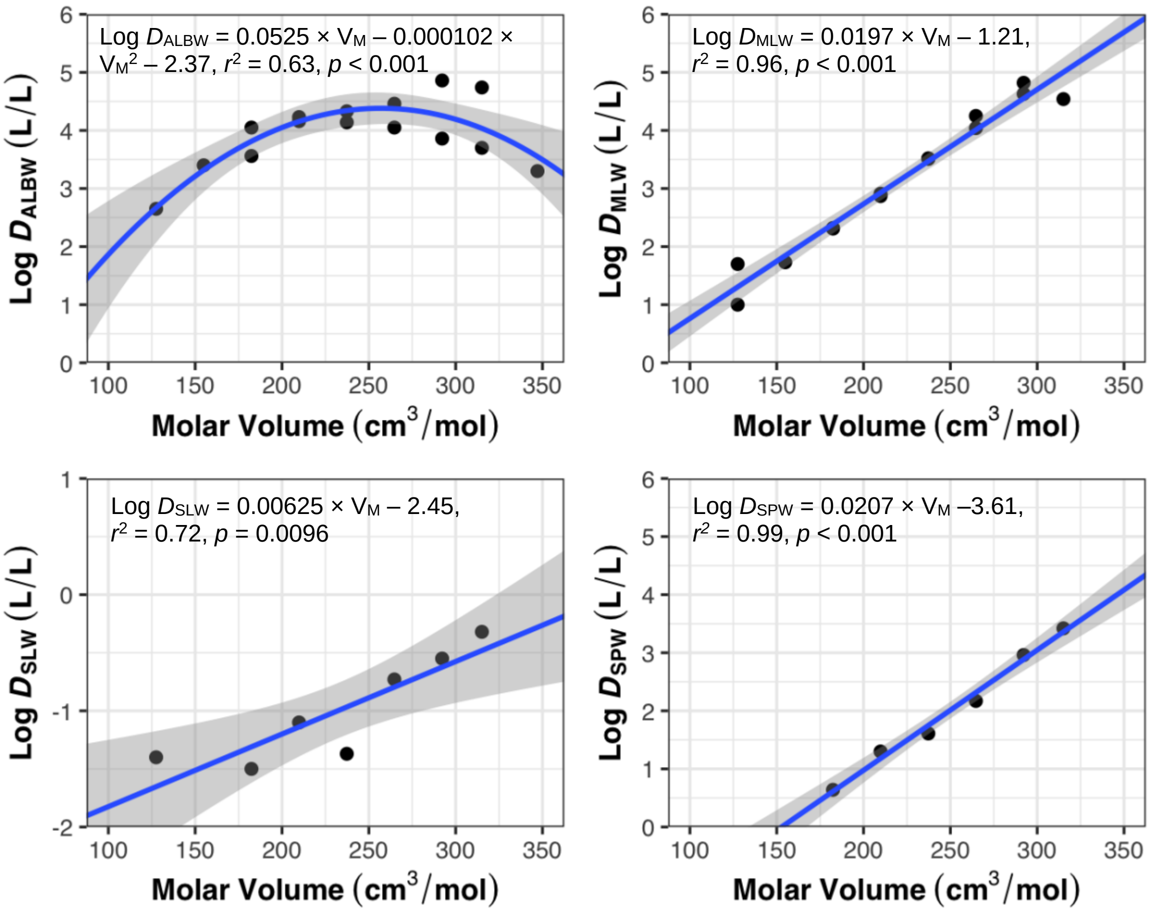

Figure S5. Relationships between measured distribution coefficients and molar volume of PFCA used to predict distribution coefficients for PFCA. Blue lines represent the linear regressions, and grey shaded areas represent the 95% confidence interval of the slope.

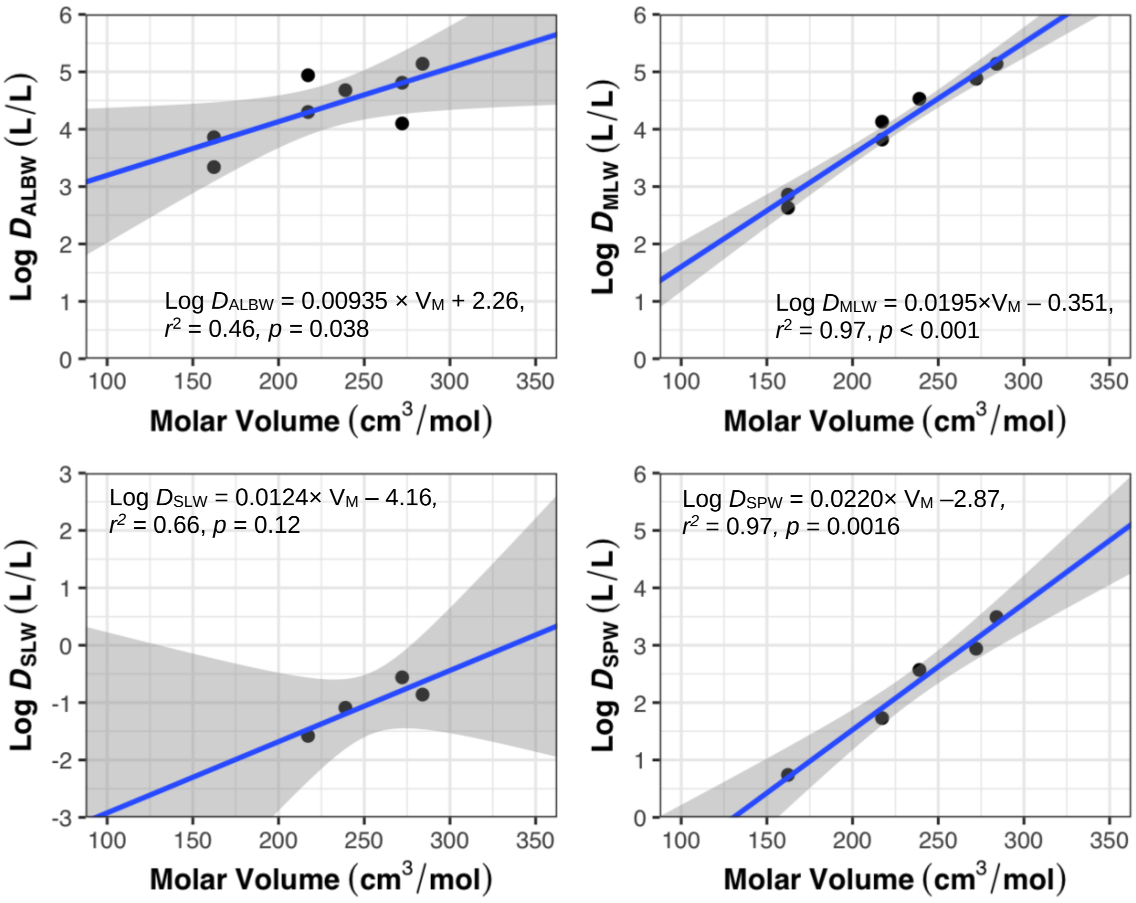

Figure S6. Relationships between measured distribution coefficients and molar volume of PFSA used to predict distribution coefficients for PFSA. Blue lines represent the linear regressions, and grey shaded areas represent the 95% confidence interval of the slope.

As substances like PFAS are typically solids at environmental temperatures, the activity coefficient must be adjusted by a fugacity ratio (*F*) in order to visualise the solid chemical behaving as a semi-cooled liquid.[1, 3] *F* (unitless) is a function of the enthalpy of fusion and the melting point of the chemical. By applying Walden's rule, which states that the enthalpy of fusion at the melting point is roughly 56.5 J/mol K, *F* can be estimated with the melting point (T_M; K) and the environmental or organism body temperature (T; K) as [1, 3]:

$$\ln F \cong -6.79\left(\frac{T_M}{T} - 1\right)$$

Melting points and water solubilities in Table S9 were obtained from the CompTox Chemicals Dashboard [37] and represent the median value if the range of predicted or experimental values was large (i.e., SD greater than 2) or the average value if the range of values was small. Experimental values were prioritised. Tissue solubilities (mol/L) for PFAS in Table S8 were calculated as the product of the water

solubility (mol/L) and the respective tissue-water distribution coefficient (L/L) in Table S9. Tissue solubilities (mol/L) for PFAS were converted to a mass basis assuming a density of 1.0 kg/L for water, 1.36 kg/L for albumin, 1.097 kg/L for polar lipids, 0.95 kg/L for neutral lipids, and 1.36 kg/L for structural protein. Solubility of PFAS within organisms was subsequently determined as:

$$S_{organism} = (\varphi_{ALB} \cdot S_{ALB} + \varphi_{NL} \cdot S_{NL} + \varphi_{PL} \cdot S_{PL} + \varphi_{SP} \cdot S_{SP} + \varphi_{W} \cdot S_{W})$$

where φ_{ALB} represents the fraction of albumin in the sample; φ_{NL} the fraction of neutral or storage lipids; φ_{PL} the fraction of polar or membrane lipids; φ_{SP} the fraction of structural protein; φ_{W} the fraction of water content; and S_{ALB}, S_{NL}, S_{PL}, S_{SP}, and S_{W} represent the solubilities of albumin, neutral lipids, polar lipids, structural proteins, and water, respectively.

Table S9. Melting points (T_M), water solubilities (S_W), and tissue solubilities (ALB = Albumin; PL = Polar Lipids; NL = Neutral Lipids; and SP = Structural Protein) used to convert concentrations of PFAS in biota to apparent chemical activities.

PFAS	T_M (K)	Log S_W (ng/g)	Log S_{ALB} (ng/g)	Log S_{PL} (ng/g)	Log S_{NL} (ng/g)	Log S_{SP} (ng/g)
PFOA	329.1	6.62	10.8	10.1	5.27	8.09
PFNA	339.5	6.11	10.4	10.3	5.41	8.15
PFDA	352.5	6.43	11.2	11.2	5.90	9.26
PFUdA	371.5	4.96	9.57	9.46	4.67	8.25
PFDoA	382.0	4.92	8.26	10.8	4.68	8.84
PFTrDA	392.0	4.45	7.11	10.9	4.41	8.91
PFTeDA	405.5	4.37	6.37	11.2	4.46	9.23
PFHxDA	384.0	4.33	3.94	12.4	4.84	10.3
PFBS	309.9	5.71	8.91	8.53	4.21	6.31
PFHxS	314.3	5.39	10.2	9.48	3.83	6.98
PFOS	324.9	5.83	10.5	10.7	5.29	8.64
PFDS	335.5	5.29	9.01	10.8	4.91	8.83

Biochemical Composition of Biota

Based on the scatterplots in the pairplot of the variables (Figure S7), we constructed individual linear regressions between TP and the mass fraction (%) of each tissue phase with particular interest in albumin and polar lipid as they appeared to have stronger correlations with TP. Absolute correlations between neutral lipid and total lipid, albumin and total protein, and structural protein and total protein were likely very high due to collinearity and from being constituents of total lipid or total protein (Figure S4).

Chemical mass of PFAS (ng) in 1 g of each tissue phase was determined by assuming that the chemical activities in each tissue phase were equivalent and represented by the activity in the organism as:

$$M_{tissue} = S_{tissue} \times \frac{a_{organism}}{F}$$

Relative fractions of chemical mass of PFAS (%) in each tissue phase were subsequently estimated from the total mass of PFAS measured in the sample as:

$$M_{tissue} = \frac{M_{tissue}}{M_W + M_{ALB} + M_{PL} + M_{NL} + M_{SP}} \times 100$$

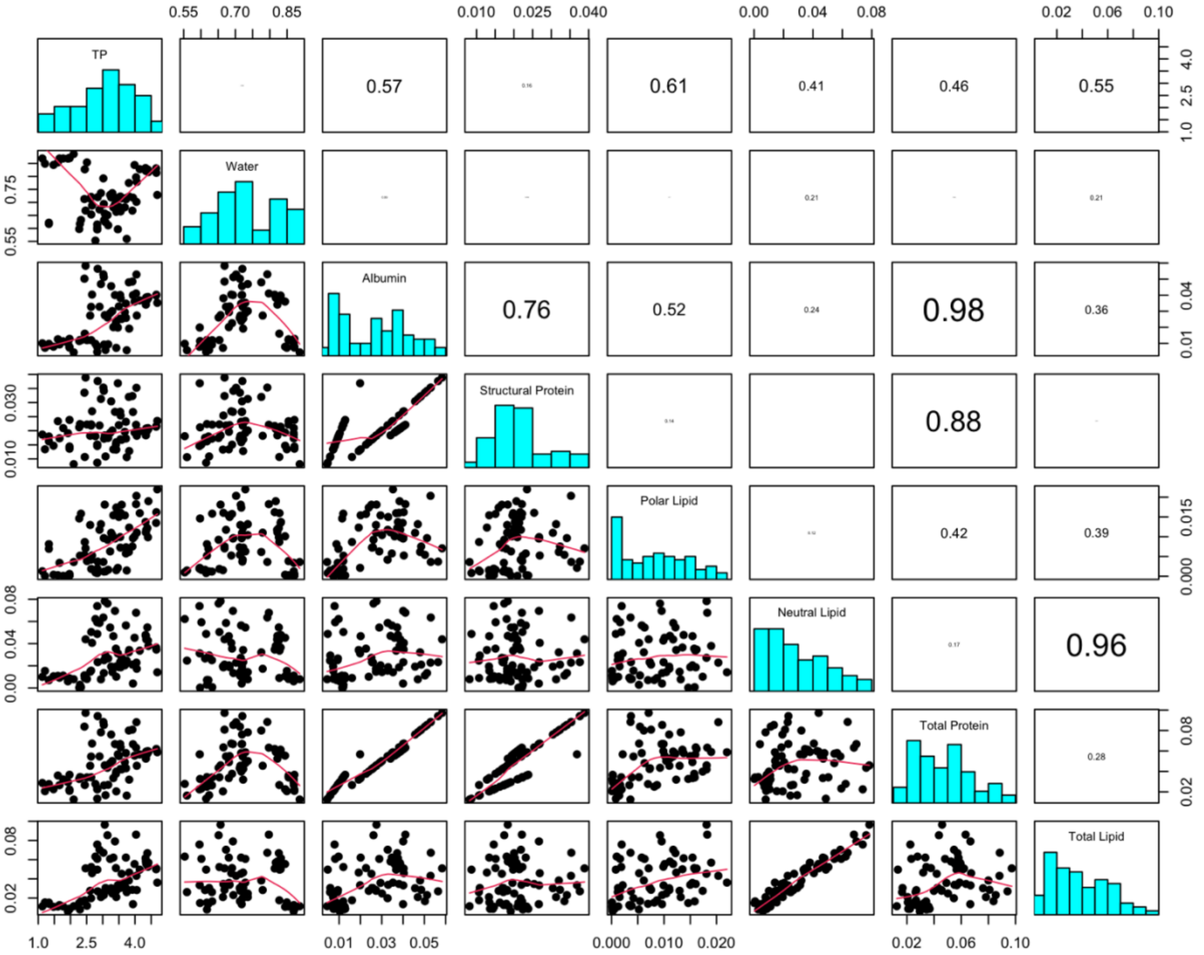

Figure S7. Pairplot of all variables - trophic position (TP) and the respective tissue components in each sample. Lower panels contain scatterplots with a LOESS smoother (red line) added to aid visual interpretation. Upper panels contain estimated pair-wise correlations with size proportional to the absolute correlation coefficient. Diagonal panels contain histograms.

Results & Discussion

Contaminant Concentrations in Air, Soil, and Biota

Table S10. Detection frequencies (%) of PFAS concentrations analysed in air, soil, and biota samples collected across Metro Vancouver, BC, in 2016. NA = Not analysed.

Species	Air	Soil	Earthworms	Oniscidae	Small Beetles	Thrushes	American Robin	Rock Pigeon	Large Beetles	Sparrows	European Starling	Northern Flicker	Cooper's Hawk
n	6	12	12	5	5	6	6	6	6	6	5	6	11
PFBA	100	42	0	0	0	0	17	0	0	0	0	17	82
PFPeA	100	25	0	20	0	0	0	0	17	0	0	0	0
PFHxA	100	33	0	0	20	0	0	0	0	17	0	17	73
PFHpA	100	33	17	40	80	0	0	17	33	0	0	0	27
PFOA	100	100	42	60	100	100	100	100	100	67	100	100	100
PFNA	100	83	25	80	100	67	83	33	100	50	40	100	100
PFDA	100	92	25	100	100	100	100	83	100	100	100	100	100
PFUdA	0	58	92	100	100	100	100	100	100	100	100	100	100
PFDoA	0	67	83	100	100	100	100	100	100	100	100	100	100
PFTrDA	0	25	100	100	100	100	100	83	100	100	100	100	100
PFTeDA	0	17	100	100	100	100	100	83	100	100	100	100	100
PFHxDA	0	NA	100	100	100	100	100	100	100	100	100	100	100
PFODA	0	NA	8	0	20	0	0	0	0	0	0	0	0
PFBS	100	17	42	20	60	83	100	83	17	83	80	67	82
PFHxS	100	17	50	60	80	100	100	100	100	100	100	100	100
PFOS	67	100	42	40	100	83	100	100	100	100	100	100	100
PFDS	0	25	25	0	80	67	100	0	67	83	80	100	100
PFEtCHxS	0	NA	25	0	20	0	0	0	83	0	0	0	36

PFAS in Air

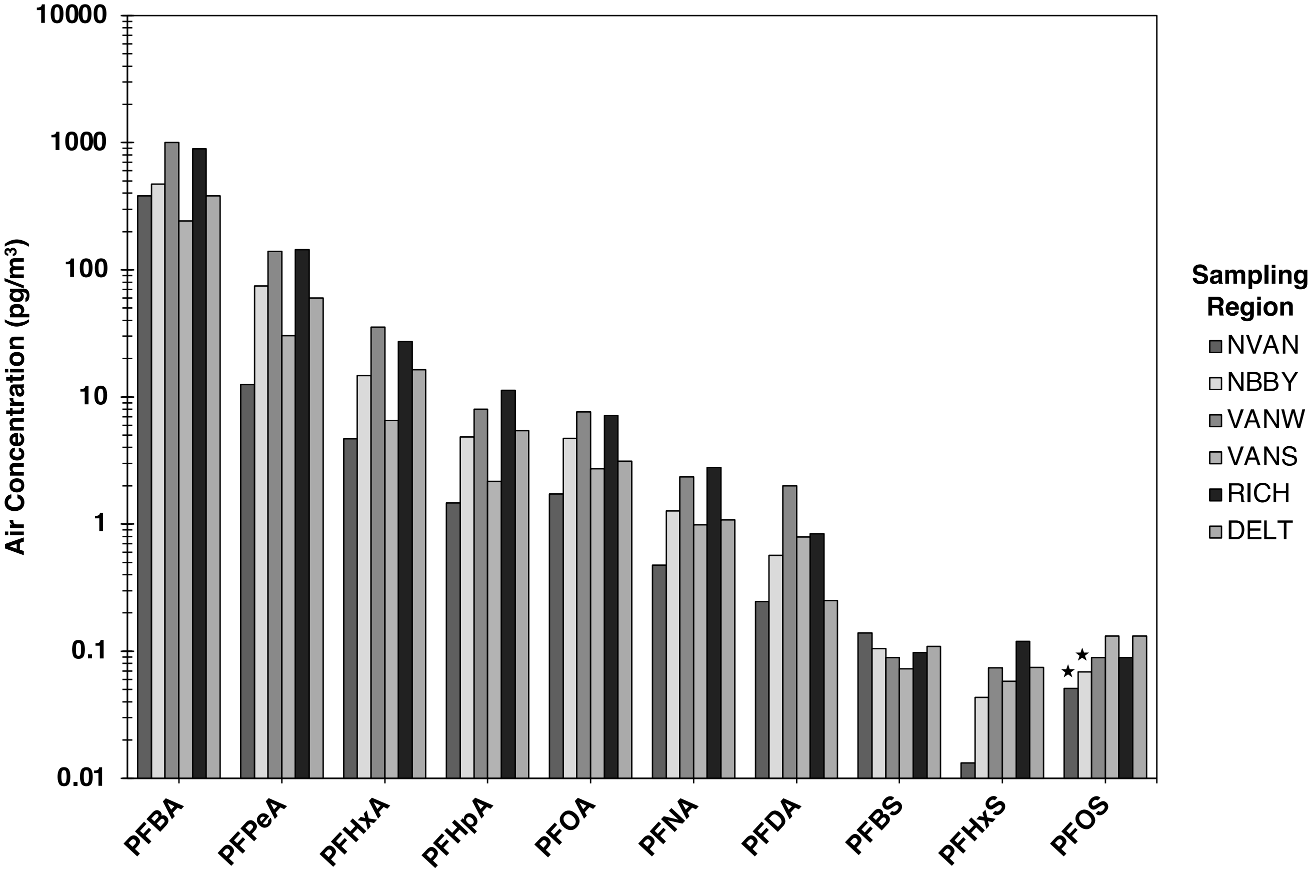

Figure S8. Concentrations (pg/m^3) of PFAS detected in air samples from six locations (*n* = 6) across Metro Vancouver, BC from September – December 2016. One sample was collected in each sampling region. NVAN = North Vancouver; NBBY = North Burnaby – East Vancouver; VANW = Vancouver – West; VANS = Vancouver – South; RICH = Richmond; DELT = Delta. ★ represent sample concentrations that were below the limit of detection.

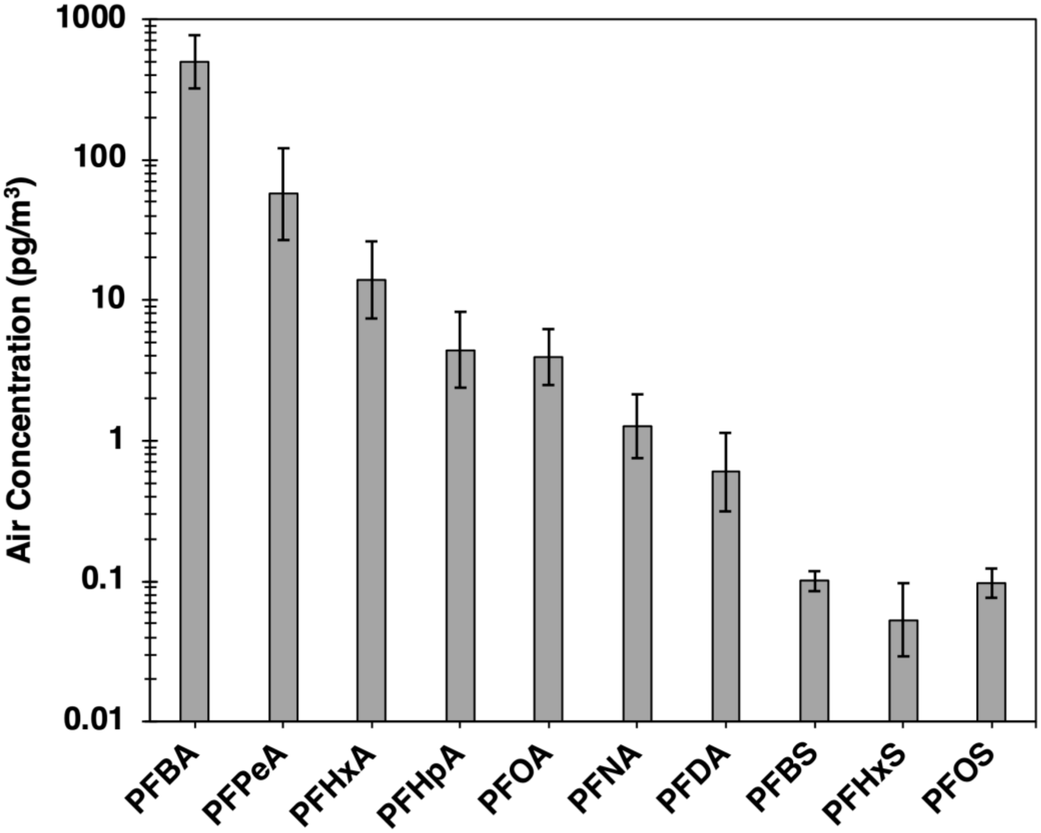

Figure S9. Geometric mean concentrations (pg/m^3) of PFAS in air samples from six locations (*n* = 6) across Metro Vancouver, 2016. Error bars represent the upper and lower 95% confidence intervals.

Geometric mean concentrations of PFCA, specifically PFHxA, PFHpA, PFOA, PFNA, and PFDA, in air samples from Metro Vancouver, BC in 2016 were generally two times higher than concentrations reported from Whistler, BC and Ucluelet, BC in 2015 and two times lower than concentrations reported from Toronto, ON in 2015 from the Global Atmospheric Passive Sampling (GAPS) Network.[41] Geometric mean concentrations of PFSA, specifically PFBS, PFHxS, and PFOS, in air samples from Metro Vancouver were 20 to 80 times lower than concentrations reported from these Canadian cities in 2015 from the GAPS Network.[41] Some of these concentration differences may be due to differences in urbanization and localized emission sources; however, these differences may also be due to differences in sampling methodology. The passive air samplers (PAS) deployed in the GAPS Network used sorbent-impregnated polyurethane foam (SIP) disks coated with XAD-4 (Supelco, Bellefonte, PA), which tend to have a greater sorptive capacity for PFCAs than the standard PUF disks that were used in our study. However, Ahrens, et al. [54] measured PFAS in air samples from Toronto using SIP-PAS and PUF-PAS and found that concentrations of PFSA from the two sampling techniques were generally within a factor of 2,

indicating good agreement between the techniques and that both methods are suitable for measuring PFAS in air.

Table S11. Geometric mean concentrations of PFAS in air (*n* = 6) and soil samples (*n* = 12) from six sampling regions of Metro Vancouver, 2016. SD = Standard Deviation; LCL = Lower 95% Confidence Limit; UCL = Upper 95% Confidence Limit.

	Air (pg/m^3)				Soil (ng/g dw)			
**PFAS**	**Mean**	**SD**	**LCL**	**UCL**	**Mean**	**SD**	**LCL**	**UCL**
PFBA	496	118	321	769	-	-	-	-
PFPeA	56.9	23.3	26.8	121	-	-	-	-
PFHxA	13.9	4.75	7.39	26.09	-	-	-	-
PFHpA	4.43	1.49	2.39	8.23	-	-	-	-
PFOA	3.95	1.00	2.48	6.29	0.0863	0.0476	0.0421	0.177
PFna	1.27	0.353	0.758	2.12	0.148	0.165	0.0788	0.278
PFDA	0.599	0.208	0.315	1.14	0.397	0.662	0.154	1.02
PFUdA	-	-	-	-	0.118	0.148	0.0580	0.240
PFDoA	-	-	-	-	0.100	0.136	0.0467	0.216
PFBS	0.100	0.00939	0.0842	0.119	-	-	-	-
PFHxS	0.0530	0.0174	0.0289	0.0971	-	-	-	-
PFOS	0.0966	0.0286	0.0684	0.131	1.30	0.510	0.782	2.17

PFAS in Soil

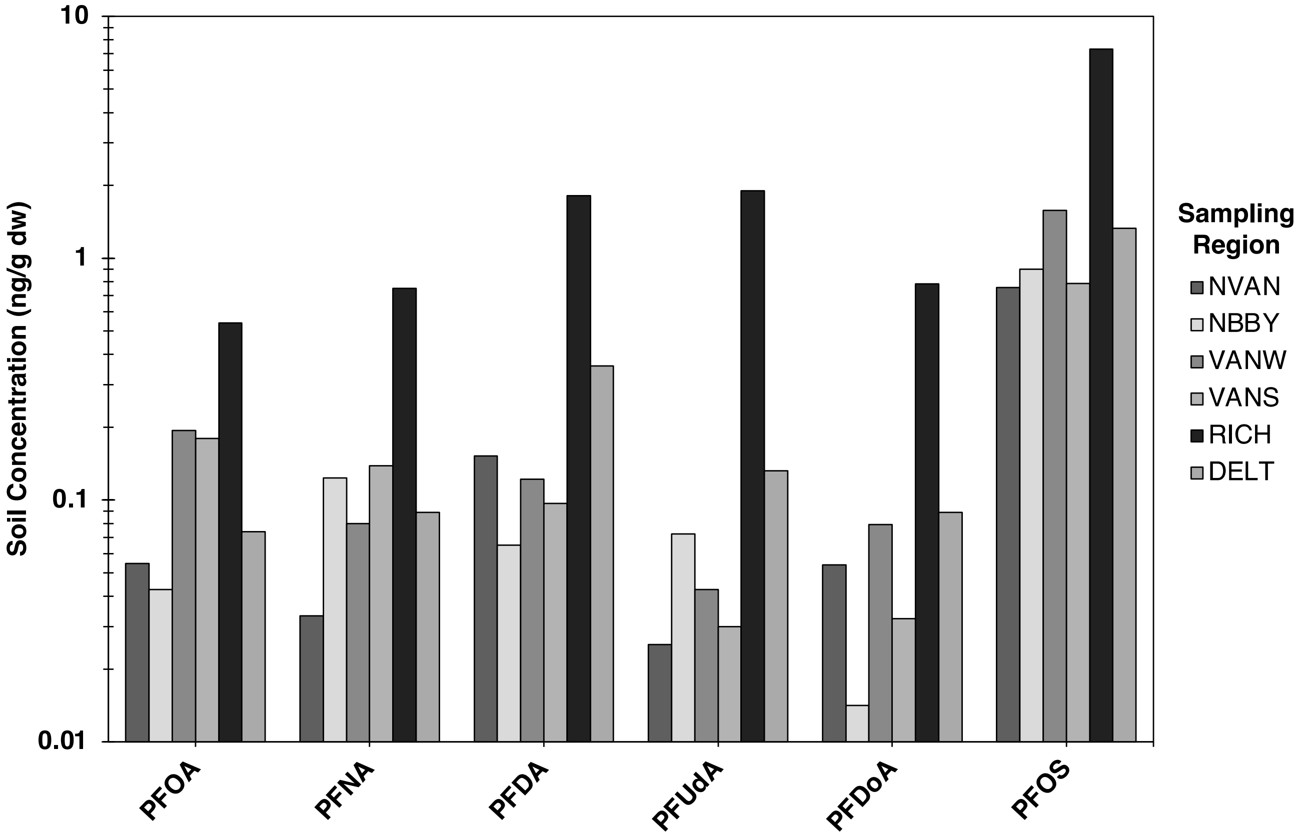

Figure S10. Mean concentrations (ng/g dry weight) of PFAS in soil samples (*n* = 2 per sampling region) collected from Metro Vancouver, BC, 2016. NVAN = North Vancouver; NBBY = North Burnaby – East Vancouver; VANW = Vancouver – West; VANS = Vancouver – South; RICH = Richmond; DELT = Delta. Peto-Peto test did not detect any differences in the mean concentrations of PFOA and PFOS in the soil samples between the sampling regions. PFOA (χ^2 = 6.89; *p* = 0.229) and PFOS (χ^2 = 15.2; *p* = 0.01; pairwise comparisons *p* > 0.05).

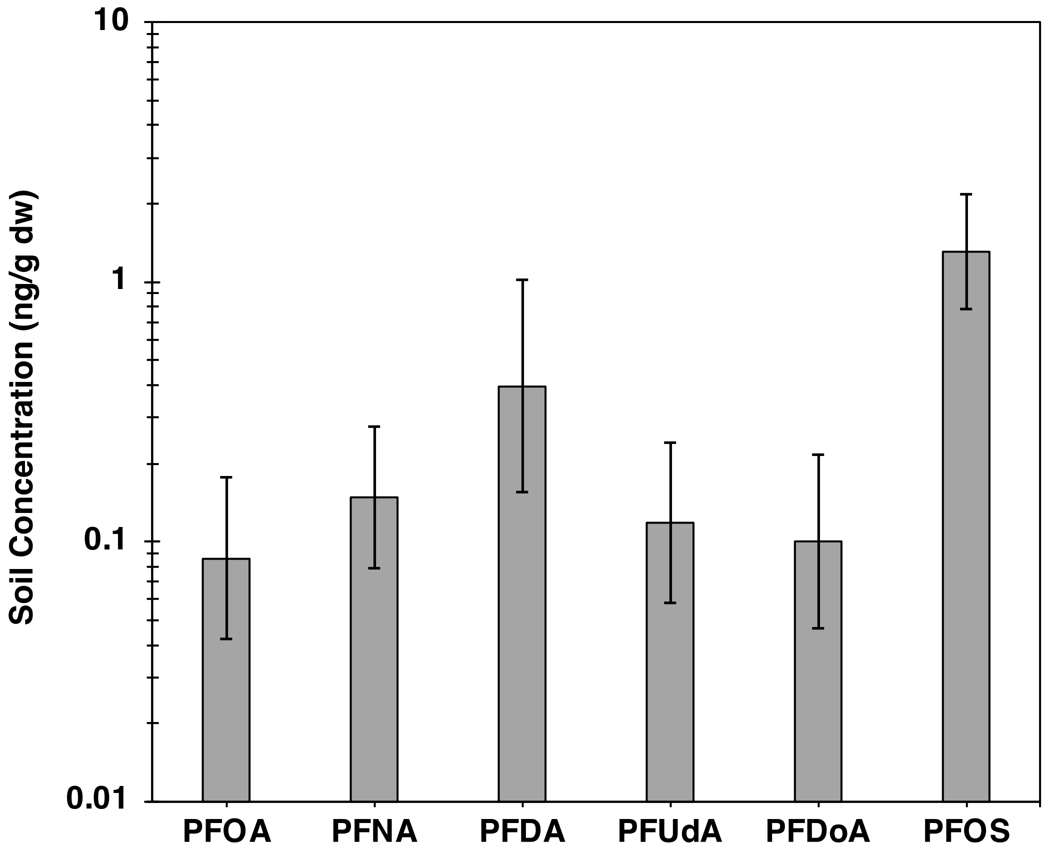

Figure S11. Geometric mean concentrations (ng/g dw) of PFAS in soil samples (*n* = 12) from Metro Vancouver, BC, 2016. Error bars represent the upper and lower 95% confidence limits. PFAS displayed had ≥ 60% detection frequency across all soil samples.

Geometric mean concentrations of PFAS in soil samples from Metro Vancouver were well within the maximum concentration ranges reported for PFOA and PFOS in surface soils from a review study comprised of over 1400 sampling locations across the world with background contamination (i.e., sites not directly impacted by PFAS sources).[55] The maximum concentrations reported at sites with background contamination for PFOA and PFOS ranged from 0.001 to 124 ng/g dw and 0.003 to 162 ng/g dw, respectively.[55] However, geometric mean concentrations of PFOA and PFOS in surface soils reported from the United States Air Force Aqueous Film-Forming Foam Impacted-Site database were 2.0 and 22 ng/g dw,[55] respectively, so approximately one to two orders of magnitude higher than the geometric mean concentrations of PFOA and PFOS in soil samples from Metro Vancouver, BC indicating that the concentrations of PFAS in the soil samples from Metro Vancouver likely represent background contamination rather than contamination from local point sources.

Table S12. Trophic magnification factors (TMFs; [95% LCL, UCL]) based on wet weight concentrations of PFAS determined for each sampling region. Overlap between the 95% CIs indicates that there were no statistical differences in the TMFs for PFAS between regions.

	Sampling Regions					
PFAS	North Vancouver	North Burnaby Vancouver East	Vancouver – West	Vancouver – South	Richmond	Ladner
**PFNA**	5.31	3.46	3.60	5.21	2.97	2.60
	[1.67, 16.85]	[1.44, 8.28]	[1.81, 7.13]	[2.67, 10.16]	[1.55, 5.71]	[1.43, 4.72]
**PFOS**	20.7	7.85	6.23	3.67	6.36	6.49
	[8.14, 52.61]	[3.50, 17.59]	[3.06, 12.70]	[1.27, 10.62]	[3.71, 10.90]	[3.04, 13.85]

PFAS in Biota

Table S13. Mean concentrations (ng/g ww) of PFAS within organisms from a terrestrial food web in Metro Vancouver, BC, 2016. Mean concentrations (SE) were estimated with the Kaplan-Meier method in NADA. WB = whole body; TP = mean trophic position (± SD); ND = non-detection.

Species	Earthworms	Oniscidae	Small Beetles	Thrushes	American Robin	Rock Pigeon	Large Beetles	Sparrows	European Starling	Northern Flicker	Cooper's Hawk
n	12	5	5	6	6	6	6	6	5	6	11
Tissue	WB	WB	WB	WB	WB	WB	WB	WB	WB	WB	Egg
TP	1.8 ± 0.11	1.9 ± 0.24	2.8 ± 0.036	2.8 ± 0.14	2.9 ± 0.14	3.2 ± 0.085	3.3 ± 0.11	3.4 ± 0.21	3.8 ± 0.11	3.9 ± 0.17	4.3 ± 0.065
PFOA	0.233 (0.0136)	0.256 (0.0403)	0.882 (0.311)	0.350 (0.103)	0.395 (0.0356)	2.84 (1.16)	0.450 (0.0657)	0.262 (0.0562)	0.246 (0.0289)	0.530 (0.177)	1.28 (0.254)
PFNA	0.0825 (0.00210)	0.124 (0.0331)	0.787 (0.457)	0.308 (0.0523)	0.295 (0.187)	1.07 (0.385)	0.380 (0.0227)	0.708 (0.412)	0.216 (0.00760)	2.30 (0.590)	2.85 (0.368)
PFDA	0.0758 (0.00590)	0.114 (0.00980)	2.20 (1.14)	0.780 (0.147)	0.145 (0.0294)	1.02 (0.401)	0.430 (0.0710)	0.845 (0.390)	0.300 (0.0622)	4.23 (1.49)	7.16 (1.26)
PFUdA	0.0933 (0.0118)	0.174 (0.0125)	1.74 (0.663)	0.875 (0.174)	0.152 (0.0255)	1.39 (0.663)	0.305 (0.0628)	0.817 (0.345)	0.316 (0.0250)	1.88 (0.525)	5.45 (0.884)
PFDoA	0.183 (0.0209)	0.356 (0.0788)	5.07 (2.26)	3.17 (0.797)	0.135 (0.0278)	1.01 (0.311)	0.545 (0.126)	1.75 (0.701)	0.910 (0.202)	6.82 (2.49)	19.8 (3.87)
PFTrDA	0.253 (0.0395)	0.360 (0.108)	4.00 (1.81)	3.00 (0.660)	0.0850 (0.0217)	0.848 (0.326)	0.342 (0.0605)	1.63 (0.690)	0.790 (0.119)	3.99 (1.38)	14.9 (2.63)
PFTeDA	0.638 (0.137)	0.384 (0.115)	7.00 (3.40)	5.33 (1.36)	0.113 (0.0325)	1.05 (0.400)	0.365 (0.0725)	2.05 (0.741)	1.57 (0.471)	8.04 (2.41)	25.2 (5.24)
PFHxDA	0.253 (0.0429)	0.256 (0.0196)	0.490 (0.233)	0.442 (0.0793)	0.258 (0.0533)	1.10 (0.625)	0.202 (0.0328)	0.188 (0.0355)	0.260 (0.0381)	0.840 (0.196)	1.04 (0.223)
PFBS	0.302 (0.0373)	0.220 (NA)	0.107 (0.0202)	0.0617 (0.0145)	0.123 (0.0551)	0.860 (0.502)	0.270 (NA)	0.135 (0.0146)	0.0940 (0.0221)	0.0500 (0.0237)	0.0727 (0.00870)
PFHxS	0.621 (0.241)	0.264 (0.0346)	0.815 (0.435)	0.523 (0.106)	0.253 (0.0208)	1.44 (0.464)	2.99 (0.627)	0.502 (0.109)	0.978 (0.776)	0.443 (0.124)	0.971 (0.112)
PFOS	0.838 (0.124)	0.752 (0.0405)	75.9 (19.2)	34.8 (10.8)	52.6 (11.2)	16.2 (3.49)	46.2 (8.48)	54.4 (22.2)	54.8 (25.5)	68.5 (17.4)	138 (14.0)
PFDS	0.438 (0.0837)	ND	0.780 (0.356)	0.880 (0.251)	ND	0.536 (0.106)	1.05 (0.460)	1.04 (0.671)	0.464 (0.153)	2.52 (1.01)	8.44 (1.48)

Biochemical Composition of Biota

Relationship with Trophic Position

Table S14. Linear relationships between trophic position and the mass fraction (%) of each respective tissue in the biota samples. Values in bold indicate statistical significance of *p*-value with α < 0.05.

Tissue	N	Slope	SE	Int	*p*-value	Adj R^2	*Pearson's *r*	AIC	BIC	F Statistic	df
Albumin	74	0.960	0.165	-0.350	**< 0.001**	0.310	0.57	251	258	33.9	1, 72
Neutral Lipid	74	0.925	0.244	0.0277	**< 0.001**	0.155	0.41	309	316	14.4	1, 72
Polar Lipid	74	0.410	0.0630	-0.438	**< 0.001**	0.362	0.61	109	115	42.3	1, 72
Structural Protein	74	0.119	0.0892	1.73	0.186	0.0106	0.16	160	167	1.78	1, 72
Total Lipid	74	1.33	0.241	-0.410	**< 0.001**	0.290	0.55	307	314	30.8	1, 72
Total Protein	74	1.08	0.244	1.38	**< 0.001**	0.202	0.46	309	316	19.5	1, 72
Water	74	-0.523	1.18	74.8	0.660	-0.0111	-0.05	543	550	0.195	1, 72

*Pearson's product moment correlation coefficient

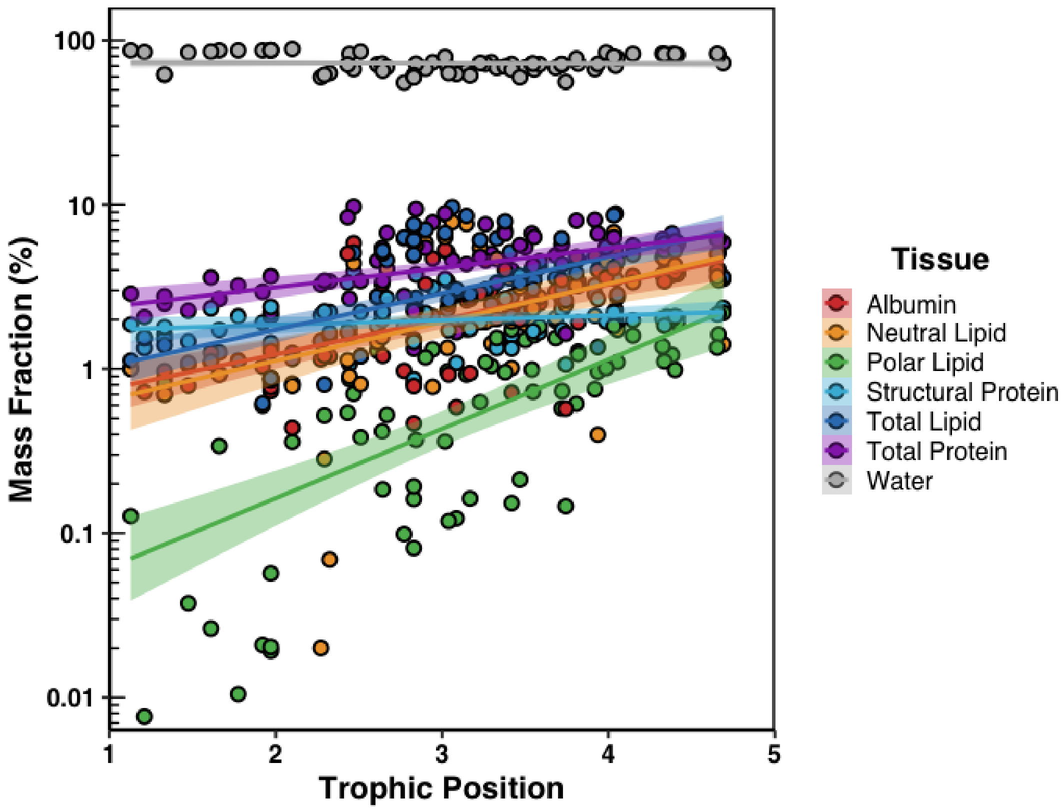

Figure S12. Mass fraction (%) of each tissue in biota samples relative to their trophic positions (TP). Coloured lines represent linear regressions between TP and the mass fraction (%) of each respective tissue.

Mass Distribution of PFAS

Since log D_{ALBW} values from Allendorf, et al. [16] differ by almost an order of magnitude from log D_{ALBW} values from Bischel, et al. [17] for some PFAS, notably PFDA and PFUdA (Table S7), we performed a sensitivity analysis using the measured log D_{ALBW} values from both studies to see if the mass distribution patterns for PFDA, PFUdA, and PFOS would change. We examined the change in the estimated mass of PFAS in each tissue type within the biota using a small range of values between the measured log D_{ALBW} values from both studies. When using log D_{ALBW} values from Bischel, et al. [17], the estimated mass of PFDA, PFUdA, and PFOS in the biota generally distributed to polar lipids by approximately 70-80% and to albumin by about 20-30%. Whereas when using values from Allendorf, et al. [16], the mass of PFDA, PFUdA, and PFOS generally distributed to albumin by approximately 70-80% and to polar lipids by about 20-40%. As there appears to be some differences in the estimated mass distributions of PFAS, we also assessed the percent change in the TMF_A (i.e., [TMF_A using Allendorf log D_{ALBW} - TMF_A using Bischel log D_{ALBW}]/TMF_A using Bischel log D_{ALBW} x100) if using the measured log D_{ALBW} values from both studies (Table S14).

Table S15. Trophic magnification factors (TMFs) for PFAS based on apparent chemical activities (TMF_A) determined using a range of log D_{ALBW} values between the measured value from Bischel, et al. [17] and the measured value from Allendorf, et al. [16].

PFAS	Log D_{ALBW}	TMF_A	LCL	UCL	*p* value	Change in TMF_A (%)
PFDA	3.86	2.94	1.97	4.40	< 0.001	11
PFDA	4.46	3.16	2.16	4.63	< 0.001	3
PFDA	4.71	3.22	2.22	4.68	< 0.001	1
PFDA	4.86	3.25	2.24	4.72	< 0.001	0
PFUdA	3.71	2.11	1.56	2.85	< 0.001	7
PFUdA	4.34	2.21	1.65	2.95	< 0.001	2
PFUdA	4.59	2.24	1.68	2.98	< 0.001	0
PFUdA	4.75	2.25	1.69	2.98	< 0.001	0
PFOS	4.1	4.76	3.18	7.13	< 0.001	8
PFOS	4.48	5.00	3.38	7.40	< 0.001	3
PFOS	4.67	5.10	3.47	7.51	< 0.001	1
PFOS	4.81	5.16	3.51	7.57	< 0.001	0

Trophic Magnification Factors

Table S16. Trophic magnification factors (TMFs) for PFAS based on apparent chemical activities (TMF_A); concentrations normalised to total protein (TMF_P); concentrations normalised to albumin (TMF_{AL}); concentrations normalised to total lipid (TMF_L); concentrations normalised to polar lipids (TMF_{PL}); wet weight concentrations (TMF_W); and dry weight concentrations (TMF_D). SE = standard error, LCL = Lower 95% confidence limit, UCL = Upper 95% confidence limit. Values in bold indicate statistical significance of *p*-value with α < 0.05.

PFAS	Slope	SE	LCL	UCL	Z Statistic	*p*-value	TMF	SE	LCL	UCL
					TMF_A					
PFOA	0.255	0.147	-0.033	0.542	1.738	0.082	1.29	0.19	0.97	1.72
PFNA	1.011	0.226	0.569	1.454	4.483	**< 0.001**	2.75	0.62	1.77	4.28
PFDA	1.178	0.190	0.806	1.549	6.212	**< 0.001**	3.25	0.62	2.24	4.71
PFUdA	0.809	0.145	0.524	1.093	5.569	**< 0.001**	2.24	0.33	1.69	2.98
PFDoA	0.712	0.201	0.319	1.105	3.549	**< 0.001**	2.04	0.41	1.38	3.02
PFTrDA	0.473	0.207	0.066	0.880	2.280	**0.023**	1.60	0.33	1.07	2.41
PFTeDA	0.453	0.228	0.007	0.899	1.990	**0.047**	1.57	0.36	1.01	2.46
PFHxDA	-0.219	0.131	-0.475	0.037	-1.677	0.094	0.80	0.10	0.62	1.04
PFBS	-0.586	0.186	-0.950	-0.222	-3.154	**0.002**	0.56	0.10	0.39	0.80
PFHxS	0.080	0.166	-0.245	0.406	0.484	0.628	1.08	0.18	0.78	1.50
PFOS	1.643	0.196	1.259	2.026	8.400	**< 0.001**	5.17	1.01	3.52	7.58
PFDS	1.072	0.289	0.506	1.638	3.712	**< 0.001**	2.92	0.84	1.66	5.14
					TMF_P					
PFOA	0.277	0.148	-0.012	0.566	1.875	0.061	1.32	0.19	0.99	1.76
PFNA	1.042	0.226	0.599	1.484	4.612	**< 0.001**	2.83	0.64	1.82	4.41
PFDA	1.199	0.187	0.832	1.566	6.400	**< 0.001**	3.32	0.62	2.30	4.79
PFUdA	0.808	0.146	0.523	1.093	5.549	**< 0.001**	2.24	0.33	1.69	2.98
PFDoA	0.955	0.180	0.602	1.308	5.295	**< 0.001**	2.60	0.47	1.82	3.70
PFTrDA	0.735	0.181	0.381	1.089	4.071	**< 0.001**	2.09	0.38	1.46	2.97
PFTeDA	0.702	0.198	0.315	1.089	3.555	**< 0.001**	2.02	0.40	1.37	2.97
PFHxDA	0.025	0.114	-0.197	0.248	0.222	0.824	1.03	0.12	0.82	1.28
PFBS	-0.555	0.187	-0.922	-0.188	-2.961	**0.003**	0.57	0.11	0.40	0.83
PFHxS	0.110	0.165	-0.213	0.434	0.669	0.503	1.12	0.18	0.81	1.54
PFOS	1.694	0.190	1.321	2.067	8.905	**< 0.001**	5.44	1.04	3.75	7.90
PFDS	1.203	0.274	0.666	1.741	4.387	**< 0.001**	3.33	0.91	1.95	5.70
					TMF_{AL}					
PFOA	0.087	0.163	-0.234	0.407	0.530	0.596	1.09	0.18	0.79	1.50
PFNA	0.849	0.238	0.381	1.316	3.559	**< 0.001**	2.34	0.56	1.46	3.73
PFDA	0.993	0.192	0.618	1.369	5.186	**< 0.001**	2.70	0.52	1.86	3.93
PFUdA	0.595	0.149	0.303	0.886	3.998	**< 0.001**	1.81	0.27	1.35	2.43

PFAS	Slope	SE	LCL	UCL	Z Statistic	*p*-value	TMF	SE	LCL	UCL
PFDoA	0.743	0.180	0.389	1.096	4.115	**< 0.001**	2.10	0.38	1.48	2.99
PFTrDA	0.522	0.180	0.169	0.875	2.895	**0.004**	1.69	0.30	1.18	2.40
PFTeDA	0.489	0.196	0.105	0.873	2.495	**0.013**	1.63	0.32	1.11	2.39
PFHxDA	-0.188	0.122	-0.427	0.051	-1.544	0.123	0.83	0.10	0.65	1.05
PFBS	-0.739	0.207	-1.144	-0.333	-3.570	**< 0.001**	0.48	0.10	0.32	0.72
PFHxS	-0.078	0.183	-0.437	0.280	-0.429	0.668	0.92	0.17	0.65	1.32
PFOS	1.484	0.191	1.110	1.859	7.769	**< 0.001**	4.41	0.84	3.03	6.41
PFDS	1.028	0.286	0.467	1.590	3.589	**< 0.001**	2.80	0.80	1.59	4.90
					**TMF_L**					
PFOA	0.025	0.118	-0.206	0.257	0.214	0.830	1.03	0.12	0.81	1.29
PFNA	0.808	0.210	0.396	1.220	3.846	**< 0.001**	2.24	0.47	1.49	3.39
PFDA	0.987	0.189	0.617	1.357	5.223	**< 0.001**	2.68	0.51	1.85	3.89
PFUdA	0.572	0.145	0.287	0.856	3.939	**< 0.001**	1.77	0.26	1.33	2.35
PFDoA	0.726	0.193	0.348	1.104	3.765	**< 0.001**	2.07	0.40	1.42	3.02
PFTrDA	0.498	0.201	0.105	0.891	2.483	**0.013**	1.65	0.33	1.11	2.44
PFTeDA	0.465	0.221	0.031	0.899	2.100	**0.036**	1.59	0.35	1.03	2.46
PFHxDA	-0.212	0.112	-0.431	0.007	-1.895	**0.058**	0.81	0.09	0.65	1.01
PFBS	-0.794	0.168	-1.123	-0.464	-4.723	**< 0.001**	0.45	0.08	0.33	0.63
PFHxS	-0.130	0.145	-0.414	0.154	-0.895	0.371	0.88	0.13	0.66	1.17
PFOS	1.446	0.170	1.113	1.780	8.498	**< 0.001**	4.25	0.72	3.04	5.93
PFDS	1.030	0.289	0.464	1.597	3.564	**< 0.001**	2.80	0.81	1.59	4.94
					**TMF_{PL}**					
PFOA	-0.240	0.214	-0.660	0.180	-1.121	0.262	0.79	0.17	0.52	1.20
PFNA	0.579	0.275	0.040	1.118	2.107	**0.035**	1.78	0.49	1.04	3.06
PFDA	0.705	0.234	0.246	1.164	3.011	**0.003**	2.02	0.47	1.28	3.20
PFUdA	0.114	0.191	-0.260	0.488	0.599	0.549	1.12	0.21	0.77	1.63
PFDoA	0.290	0.217	-0.136	0.715	1.335	0.182	1.34	0.29	0.87	2.05
PFTrDA	0.036	0.225	-0.405	0.478	0.160	0.873	1.04	0.23	0.67	1.61
PFTeDA	0.003	0.246	-0.478	0.485	0.013	0.990	1.00	0.25	0.62	1.62
PFHxDA	-0.674	0.166	-0.999	-0.349	-4.061	**< 0.001**	0.51	0.08	0.37	0.71
PFBS	-1.057	0.254	-1.556	-0.558	-4.153	**< 0.001**	0.35	0.09	0.21	0.57
PFHxS	-0.427	0.233	-0.883	0.029	-1.835	**0.067**	0.65	0.15	0.41	1.03
PFOS	1.180	0.229	0.731	1.629	5.152	**< 0.001**	3.25	0.75	2.08	5.10
PFDS	0.712	0.344	0.039	1.385	2.072	**0.038**	2.04	0.70	1.04	4.00
PFDS	1.002	0.293	0.428	1.576	3.419	**0.001**	2.72	0.80	1.53	4.84
					**TMF_W**					
PFOA	0.512	0.122	0.273	0.751	4.199	**< 0.001**	1.67	0.20	1.31	2.12
PFNA	1.278	0.204	0.877	1.678	6.255	**< 0.001**	3.59	0.73	2.40	5.36
PFDA	1.464	0.184	1.103	1.826	7.947	**< 0.001**	4.33	0.80	3.01	6.21

PFAS	Slope	SE	LCL	UCL	Z Statistic	*p*-value	TMF	SE	LCL	UCL
PFUdA	1.082	0.145	0.798	1.366	7.473	**< 0.001**	2.95	0.43	2.22	3.92
PFDoA	1.229	0.186	0.865	1.593	6.616	**< 0.001**	3.42	0.63	2.37	4.92
PFTrDA	1.009	0.189	0.639	1.379	5.342	**< 0.001**	2.74	0.52	1.89	3.97
PFTeDA	0.976	0.207	0.570	1.381	4.718	**< 0.001**	2.65	0.55	1.77	3.98
PFHxDA	0.299	0.099	0.104	0.493	3.013	**0.003**	1.35	0.13	1.11	1.64
PFBS	-0.331	0.153	-0.631	-0.030	-2.157	**0.031**	0.72	0.11	0.53	0.97
PFHxS	0.350	0.135	0.085	0.615	2.589	**0.010**	1.42	0.19	1.09	1.85
PFOS	1.967	0.190	1.594	2.340	10.340	**< 0.001**	7.15	1.36	4.92	10.4
PFDS	1.413	0.260	0.905	1.922	5.446	**< 0.001**	4.11	1.07	2.47	6.83
					**TMF_D**					
PFOA	0.512	0.133	0.252	0.773	3.851	**< 0.001**	1.67	0.22	1.29	2.17
PFNA	1.289	0.217	0.864	1.714	5.949	**< 0.001**	3.63	0.79	2.37	5.55
PFDA	1.480	0.198	1.092	1.868	7.474	**< 0.001**	4.39	0.87	2.98	6.47
PFUdA	1.030	0.155	0.726	1.335	6.630	**< 0.001**	2.80	0.44	2.07	3.80
PFDoA	1.179	0.200	0.787	1.572	5.889	**< 0.001**	3.25	0.65	2.20	4.82
PFTrDA	0.951	0.207	0.545	1.357	4.589	**< 0.001**	2.59	0.54	1.72	3.88
PFTeDA	0.918	0.229	0.468	1.367	4.002	**< 0.001**	2.50	0.57	1.60	3.92
PFHxDA	0.241	0.117	0.012	0.469	2.065	**0.039**	1.27	0.15	1.01	1.60
PFBS	-0.338	0.170	-0.671	-0.005	-1.989	**0.047**	0.71	0.12	0.51	1.00
PFHxS	0.315	0.143	0.034	0.595	2.200	**0.028**	1.37	0.20	1.03	1.81
PFOS	1.924	0.185	1.562	2.286	10.423	**< 0.001**	6.85	1.26	4.77	9.83
PFDS	1.485	0.295	0.907	2.062	5.037	**< 0.001**	4.41	1.30	2.48	7.86

Literature Comparisons

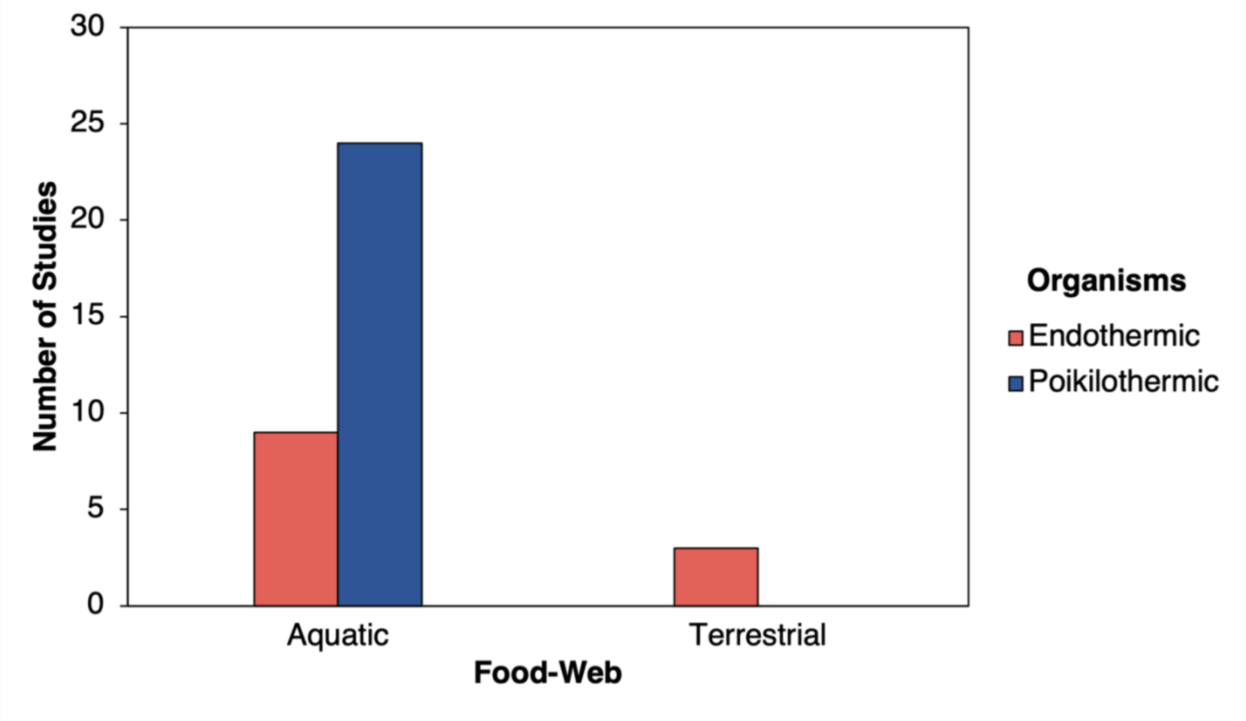

Figure S13. Number of studies that have determined TMFs based on wet and/or dry weight concentrations of PFAS in aquatic and terrestrial food-webs with either exclusively poikilothermic organisms or a combination of endothermic and poikilothermic organisms. Aquatic studies which included poikilothermic species: Bergman [56], Chen, et al. [57], Chu, et al. [58], Fang, et al. [59], Li, et al. [60], Loi, et al. [61], Martin, et al. [62], Mazzoni, et al. [63], Miranda, et al. [64], Munoz, et al. [65], Munoz, et al. [66], Pan, et al. [67], Penland, et al. [68], Ren, et al. [69], Ren, et al. [70], Simonet-Laprade, et al. [71], Simonnet-Laprade, et al. [72], Teunen, et al. [73], Zhang, et al. [74], Houde, et al. [75], Langberg, et al. [76], Du, et al. [77], and Wang, et al. [78]; and aquatic studies which included endothermic and poikilothermic species: Chen, et al. [79], Kelly, et al. [80], Tomy, et al. [81], Xu, et al. [82], Zhang, et al. [74], Houde, et al. [83], Tomy, et al. [84], Gao, et al. [85], and Xiong [86]. Terrestrial studies included Huang, et al. [87], Müller, et al. [88], and this study.

Table S17. Trophic magnification factors of PFAS from terrestrial and aquatic food-webs determined with wet weight concentrations (TMF_W), dry weight concentrations (TMF_D), and concentrations normalised to total protein content (TMF_P).

Reference	PFBA	PFHxA	PFHpA	PFOA	PFNA	PFDA	PFUdA	PFDoA	PFTrDA	PFTeDA	PFHxDA	PFBS	PFHxS	PFHpS	PFOS	PFDS
**TMF_Ws and TMF_Ds from Terrestrial Food Webs with Avian Species**																
This Study (TMF_W)	—	—	—	1.67	3.59	4.33	2.95	3.42	2.74	2.65	1.3	0.72	1.42	—	7.15	4.11
This Study (TMF_D)	—	—	—	1.67	3.63	4.39	2.8	3.25	2.59	2.5	1.27	0.71	1.37	—	6.85	4.41
Huang, et al. [87](TMF_D)	5.11	—	—	—	—	4.7	4.4	1.8	—	—	—	6	2.4	—	5.8	—
**n (TMF_D)**	1	—	—	1	1	2	2	2	1	1	1	2	2	—	2	1
**Geometric Mean**	5.11	—	—	1.67	3.61	4.47	3.31	2.71	2.66	2.57	1.28	1.45	1.67	—	6.57	4.26
**Median**	5.11	—	—	1.67	3.61	4.39	2.95	3.25	2.67	2.58	1.29	0.72	1.42	—	6.85	4.26
**TMF_Ws from Terrestrial Food Web with only Mammalian Species†**																
Müller, et al. [88]	—	—	—	1.32	2.68	2.6	2.54	1.41	1.43	—	—	—	—	—	2.65	—
Müller, et al. [88]	—	—	—	1.31	2.15	2.3	2.8	2.19	1.97	—	—	—	—	—	2.42	—
Müller, et al. [88]	—	—	—	1.1	1.95	2.27	2.25	1.35	1.37	—	—	—	—	—	2.24	—
Müller, et al. [88]	—	—	—	1.28	1.88	2.29	2.93	2.02	1.8	—	—	—	—	—	2.31	—
**n**	—	—	—	4	4	4	4	4	4	—	—	—	—	—	4	—
**Geometric Mean**	—	—	—	1.25	2.14	2.36	2.62	1.70	1.62	—	—	—	—	—	2.40	—
**Median**	—	—	—	1.30	2.05	2.30	2.67	1.72	1.62	—	—	—	—	—	2.37	—
**TMF_Ws from Aquatic Food Webs‡**																
Kelly, et al. [89]	—	—	1.43	3.28	7.03	8.29	7.98	4.79	—	2.37	—	—	—	—	17.4	—
Munoz, et al. [66] P	—	—	—	0.40	1.43	1.63	1.50	1.48	1.20	1.57	—	—	1.05	3.68	1.60	1.03
**n**	—	—	1	2	2	2	2	2	1	2	—	—	1	1	2	1
**Geometric Mean**	—	—	1.43	1.15	3.17	3.68	3.46	2.66	1.20	1.93	—	—	1.05	3.68	5.28	1.03
**Median**	—	—	1.43	1.84	4.23	4.96	4.74	3.135	1.20	1.97	—	—	1.05	3.68	9.50	1.03
**TMF_Ps from Aquatic Food Webs§**																
Kelly, et al. [89]	—	—	0.76	1.93	4.23	4.81	4.79	2.96	—	1.97	—	—	—	—	11	—
Kelly, et al. [89]	—	—	0.75	0.40	0.63	0.60	1.09	1.01	—	0.34	—	—	—	—	0.47	—
Munoz, et al. [66]	—	—	—	0.40	1.09	1.78	1.59	1.25	1.24	1.20	—	—	0.60	2.72	1.86	1.28

Reference	PFBA	PFHxA	PFHpA	PFOA	PFNA	PFDA	PFUdA	PFDoA	PFTrDA	PFTeDA	PFHxDA	PFBS	PFHxS	PFHpS	PFOS	PFDS
**n**	—	—	2	3	3	3	3	3	1	3	—	—	1	1	3	1
**Geometric Mean**	—	—	0.75	0.68	1.43	1.73	2.02	1.55	1.24	0.93	—	—	0.60	2.72	2.13	1.28
**Median**	—	—	0.76	0.4	1.09	1.78	1.59	1.25	1.24	1.2	—	—	0.60	2.72	1.86	1.28
**TMF_{P}s from Terrestrial Food Web**																
This Study	—	—	—	1.32	2.83	3.32	2.24	2.6	2.09	2.02	1.03	0.57	1.12	—	5.44	3.33

†TMF_{W}s based on whole body burdens from Porcupine caribou-wolf-lichen, Bathurst caribou-wolf-lichen, Porcupine caribou-wolf-vegetation, and Bathurst caribou-wolf-vegetation food-webs, respectively.

‡ TMF_{W}s reported by Kelly, et al. [89] based on concentrations in endotherms and poikilotherms; and TMF_{W}s reported by Munoz, et al. [66] based on concentrations in only poikilotherms but include concentrations in vegetation samples.

§TMF_{P}s reported by Kelly, et al. [89] based on concentrations in endotherms and poikilotherms and afterwards only in poikilotherms; TMF_{P}s reported by Munoz, et al. [66] based on concentrations in only poikilotherms but exclude all concentrations in vegetation samples with low protein content.

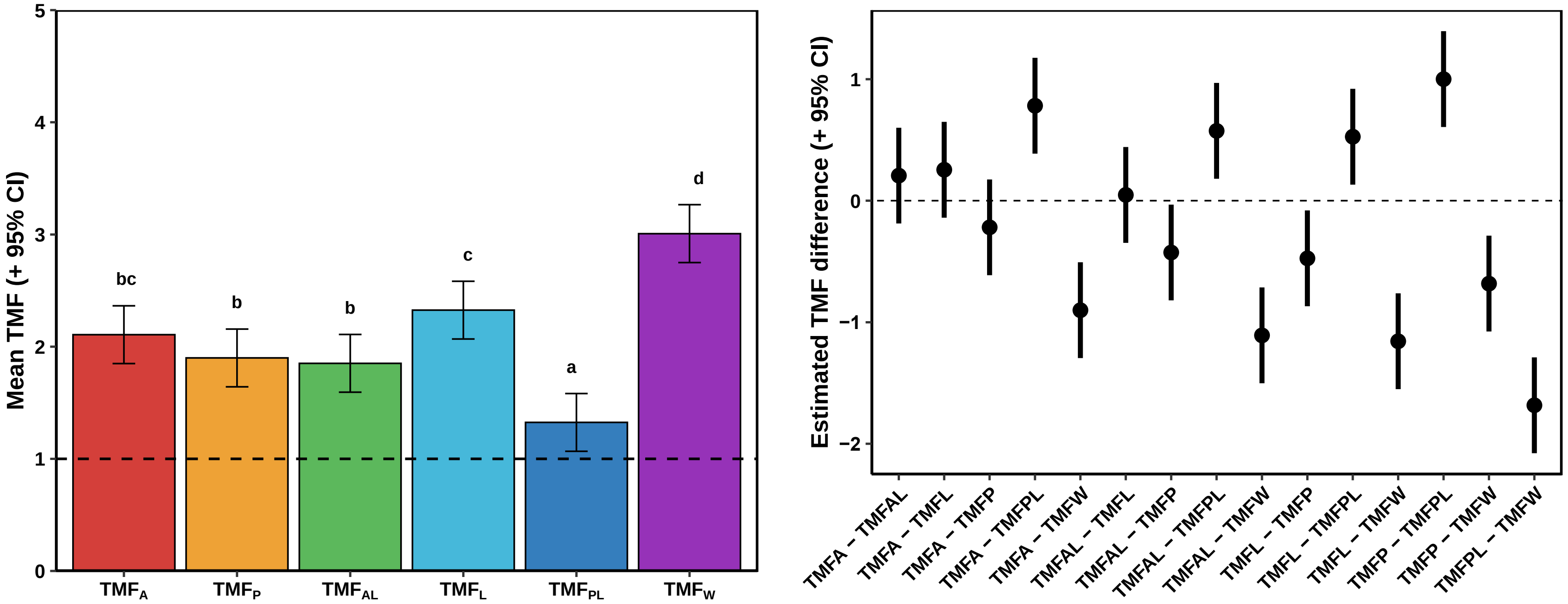

Figure S14. (Left) Comparison of mean TMFs averaged over all PFAS with compact letter display. Bars represent least square (LS) mean value for each TMF method following a one-way analysis of variance (ANOVA). Error bars indicate 95% confidence intervals of the LS means. Means sharing a letter are not statistically different (α = 0.05, Tukey-adjusted). (Right) Estimated pairwise differences in mean TMFs averaged over all PFAS.

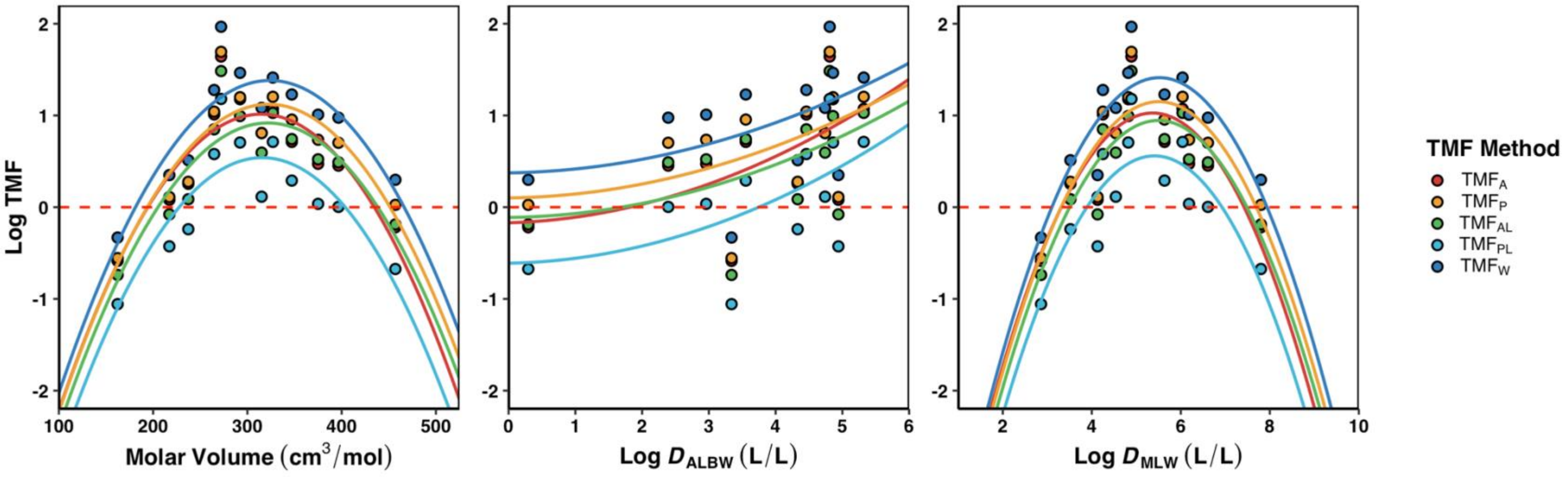

Figure S15. Relationships between observed TMFs and physicochemical properties of PFAS. Coloured solid lines represent separate regressions for all PFAS analysed with each TMF method (TMF_A = TMF based on apparent chemical activities, TMF_P =TMF based on concentrations normalised to total protein, TMF_{AL} =TMF based on concentrations normalised to albumin, TMF_{PL} =TMF based on concentrations normalised to polar lipids, TMF_W =TMF based on wet weight concentrations). Red dashed line represents TMF = 1. D_{ALBW} = distribution coefficient of albumin-water and D_{MLW} = distribution coefficient of membrane or polar lipid-water.

References

(1) Mackay, D. *Multimedia Environmental Models: The Fugacity Approach, Second Edition*; CRC Press 2001. DOI: 10.1201/9781420032543

(2) MacKay, D. Finding fugacity feasible, fruitful, and fun. *Environ Toxicol Chem* **2004**, *23* (10), 2282-2289. DOI: 10.1897/03-465.

(3) Gobas, F. A. P. C.; P. Mayer; T. F. Parkerton; R. M. Burgess; D. van de Meent; T. Gouin. A chemical activity approach to exposure and risk assessment of chemicals. *Environmental Toxicology and Chemistry* **2018**, *37* (5), 1235-1251, https://doi.org/10.1002/etc.4091. DOI: https://doi.org/10.1002/etc.4091 (acccessed 2022/08/10).

(4) Gobas, F. A. P. C.; S. Xu; G. Kozerski; D. E. Powell; K. B. Woodburn; D. Mackay; A. Fairbrother. Fugacity and activity analysis of the bioaccumulation and environmental risks of decamethylcyclopentasiloxane (D5). *Environmental Toxicology and Chemistry* **2015**, *34* (12), 2723-2731, https://doi.org/10.1002/etc.2942. DOI: https://doi.org/10.1002/etc.2942 (acccessed 2022/03/15).

(5) Gobas, F. A. P. C.; J. R. McCorquodale; G. D. Haffner. Intestinal absorption and biomagnification of organochlorines. *Environ Toxicol Chem* **1993**, *12*, 567-576.

(6) Gobas, F. A. P. C.; X. Zhang; R. Wells. Gastrointestinal magnification: the mechanism of biomagnification and food chain accumulation of organic chemicals. *Environ. Sci. Technol.* **1993**, *27* (13), 2855-2863. DOI: 10.1021/es00049a028.

(7) Kelly, B. C.; F. A. Gobas. Bioaccumulation of persistent organic pollutants in lichen-caribou-wolf food chains of Canada's Central and Western Arctic. *Environmental Science & Technology* **2001**, *35* (2), 325. DOI: 10.1021/es0011966.

(8) deBruyn, A. M. H.; F. A. P. C. Gobas. The sorptive capacity of animal protein. *Environmental Toxicology and Chemistry* **2007**, *26* (9), 1803-1808. DOI: 10.1897/07-016R.1.

(9) Kelly, B. C.; F. A. P. C. Gobas. An arctic terrestrial food-chain bioaccumulation model for persistent organic pollutants. *Environmental Science & Technology* **2003**, *37* (13), 2966. DOI: 10.1021/es021035x.

(10) Kelly, B. C.; M. G. Ikonomou; J. D. Blair; A. E. Morin; F. A. Gobas. Food web-specific biomagnification of persistent organic pollutants. *Science* **2007**, *317* (5835), 236-239. DOI: 10.1126/science.1138275.

(11) Fremlin, K. M.; J. E. Elliott; D. J. Green; K. G. Drouillard; T. Harner; A. Eng; F. A. P. C. Gobas. Trophic magnification of legacy persistent organic pollutants in an urban terrestrial food web. *Science of The Total Environment* **2020**, *714*, 136746. DOI: 10.1016/j.scitotenv.2020.136746.

(12) OECD. *Test No. 305: Bioaccumulation in Fish: Aqueous and Dietary Exposure*; 2012. DOI: doi:https://doi.org/10.1787/9789264185296-en.

(13) Borgå, K.; K. A. Kidd; D. C. Muir; O. Berglund; J. M. Conder; F. A. Gobas; J. Kucklick; O. Malm; D. E. Powell. Trophic magnification factors: considerations of ecology, ecosystems, and study design. *Integr Environ Assess Manag* **2012**, *8* (1), 64-84. DOI: 10.1002/ieam.244.

(14) Kidd, K. A.; L. P. Burkhard; M. Babut; K. Borgå; D. C. G. Muir; O. Perceval; H. Ruedel; K. Woodburn; M. R. Embry. Practical advice for selecting or determining trophic magnification factors for application under the European Union Water Framework Directive. *Integrated Environmental Assessment and Management* **2019**, *15* (2), 266-277, https://doi.org/10.1002/ieam.4102. DOI: https://doi.org/10.1002/ieam.4102 (acccessed 2022/10/20).

(15) Burkhard, L. P.; J. A. Arnot; M. R. Embry; K. J. Farley; R. A. Hoke; M. Kitano; H. A. Leslie; G. R. Lotufo; T. F. Parkerton; K. G. Sappington; G. T. Tomy; K. B. Woodburn. Comparing

laboratory and field measured bioaccumulation endpoints. *Integrated Environmental Assessment and Management* **2012**, *8* (1), 17-31. DOI: https://doi.org/10.1002/ieam.260.
(16) Allendorf, F.; K. U. Goss; N. Ulrich. Estimating the Equilibrium Distribution of Perfluoroalkyl Acids and 4 of Their Alternatives in Mammals. *Environ Toxicol Chem* **2021**, *40* (3), 910-920. DOI: 10.1002/etc.4954 From NLM.
(17) Bischel, H. N.; L. A. MacManus-Spencer; C. Zhang; R. G. Luthy. Strong associations of short-chain perfluoroalkyl acids with serum albumin and investigation of binding mechanisms. *Environmental Toxicology and Chemistry* **2011**, *30* (11), 2423-2430, https://doi.org/10.1002/etc.647. DOI: https://doi.org/10.1002/etc.647 (acccessed 2022/06/22).
(18) Droge, S. T. J. Membrane–Water Partition Coefficients to Aid Risk Assessment of Perfluoroalkyl Anions and Alkyl Sulfates. *Environmental Science & Technology* **2019**, *53* (2), 760-770. DOI: 10.1021/acs.est.8b05052.
(19) Armitage, J. M.; J. A. Arnot; F. Wania. Potential role of phospholipids in determining the internal tissue distribution of perfluoroalkyl acids in biota. *Environmental Science & Technology* **2012**, *46* (22), 12285. DOI: 10.1021/es304430r.
(20) Conder, J. M.; R. A. Hoke; W. d. Wolf; M. H. Russell; R. C. Buck. Are PFCAs Bioaccumulative? A Critical Review and Comparison with Regulatory Criteria and Persistent Lipophilic Compounds. *Environmental Science & Technology* **2008**, *42* (4), 995-1003. DOI: 10.1021/es070895g.
(21) Miller, A.; J. E. Elliott; K. H. Elliott; M. F. Guigueno; L. K. Wilson; S. Lee; A. Idrissi. Spatial and temporal trends in brominated flame retardants in seabirds from the Pacific coast of Canada. *Environmental Pollution* **2014**, *195*, 48-55. DOI: http://dx.doi.org/10.1016/j.envpol.2014.08.009.
(22) Henny, C. J.; J. E. Elliott. Chapter 18, Toxicology. In *Raptor Research and Management Techniques. Second Edition*, Bird , D. M., Bildstein, K. L. Eds.; Raptor Research Foundation. Hancock House Publishing, 2007; pp 351 - 364.
(23) Elliott, J. E.; P. A. Martin. Chlorinated hydrocarbons and shell thinning in eggs of (*Accipiter*) hawks in Ontario, 1986–1989. *Environmental Pollution* **1994**, *86* (2), 189-200. DOI: http://dx.doi.org/10.1016/0269-7491(94)90190-2.
(24) Cava, J. A.; A. C. Stewart; R. N. Rosenfield. Introduced species dominate the diet of breeding urban Cooper's hawks in British Columbia. *The Wilson Journal of Ornithology* **2012**, *124* (4), 775-782. DOI: 10.1676/1559-4491-124.4.775.
(25) Bielefeldt, J.; R. N. Rosenfield; J. M. Papp. Unfounded Assumptions about Diet of the Cooper's Hawk. *The Condor* **1992**, *94* (2), 427-436. DOI: 10.2307/1369215.
(26) Curtis, O. E.; R. N. Rosenfield; J. Bielefeldt. Cooper's Hawk (*Accipiter cooperii*). In *The Birds of North America*; Rodewald, P. G., Ed.; Cornell Lab of Ornithology, 2006.
(27) Estes, W. A.; R. W. Mannan. Feeding Behavior of Cooper's Hawks at Urban and Rural Nests in Southeastern Arizona. *The Condor* **2003**, *105* (1), 107-116.
(28) Rosenfield, R. N.; K. K. Madden; J. Bielefeldt; O. E. Curtis. Cooper's Hawk (Accipiter cooperii), version 1.0. In *Birds of the World*; Rodewald, P. G., Ed.; Cornell Lab of Ornithology, 2020.
(29) Roth, T. C.; S. L. Lima. Hunting behavior and diet of Cooper's hawks: An urban view of the small-bird-in-winter paradigm. *The Condor* **2003**, *105* (3), 474-483. DOI: 10.1650/7219 (acccessed 2015/11/13).
(30) Cabe, P. R. European Starling (*Sturnus vulgaris*). In *The Birds of North America*; Rodewald, P. G., Ed.; Cornell Lab of Ornithology, 1993.
(31) Fischl, J.; D. F. Caccamise. Relationships of Diet and Roosting Behavior in the European Starling. *The American Midland Naturalist* **1987**, *117* (2), 395-404. DOI: 10.2307/2425982.
(32) Currier, H. A. Exposure to brominated flame retardants and their associated effects on the growth and development and breeding success in two avian models; the zebra finch and the European starling. Simon Fraser University, Burnaby, BC, 2014.

(33) Wheelwright, N. T. The Diet of American Robins: An Analysis of U.S. Biological Survey Records. *The Auk* **1986**, *103* (4), 710-725.
(34) Witmer, M. C. Annual Diet of Cedar Waxwings Based on U.S. Biological Survey Records (1885-1950) Compared to Diet of American Robins: Contrasts in Dietary Patterns and Natural History. *The Auk* **1996**, *113* (2), 414-430. DOI: 10.2307/4088908.
(35) Eastman, J. *The Eastman Guide to Birds: Natural History Accounts for 150 North American Species*; Stackpole Books, 2000.
(36) Schuster, J. K.; T. Harner; G. Fillmann; L. Ahrens; J. C. Altamirano; B. Aristizábal; W. Bastos; L. E. Castillo; J. Cortés; O. Fentanes; A. Gusev; M. Hernandez; M. n. V. Ibarra; N. B. Lana; S. C. Lee; A. P. Martínez; K. S. B. Miglioranza; A. P. Puerta; F. Segovia; M. Siu; M. Y. Tominaga. Assessing Polychlorinated Dibenzo-p-dioxins and Polychlorinated Dibenzofurans in Air across Latin American Countries Using Polyurethane Foam Disk Passive Air Samplers. *Environmental Science & Technology* **2015**, *49* (6), 3680-3686. DOI: 10.1021/es506071n.
(37) Williams, A. J.; C. M. Grulke; J. Edwards; A. D. McEachran; K. Mansouri; N. C. Baker; G. Patlewicz; I. Shah; J. F. Wambaugh; R. S. Judson; A. M. Richard. The CompTox Chemistry Dashboard: a community data resource for environmental chemistry. *Journal of Cheminformatics* **2017**, *9* (1), 61. DOI: 10.1186/s13321-017-0247-6.
(38) Comptox Chemicals Dashboard v2.2.1. United States Environmental Protection Agency. https://comptox.epa.gov/dashboard/ (accessed.
(39) Lampic, A.; J. M. Parnis. Property Estimation of Per- and Polyfluoroalkyl Substances: A Comparative Assessment of Estimation Methods. *Environmental Toxicology and Chemistry* **2020**, *39* (4), 775-786, https://doi.org/10.1002/etc.4681. DOI: https://doi.org/10.1002/etc.4681 (acccessed 2022/03/10).
(40) Shoeib, M.; J. Schuster; C. Rauert; K. Su; S.-A. Smyth; T. Harner. Emission of poly and perfluoroalkyl substances, UV-filters and siloxanes to air from wastewater treatment plants. *Environmental Pollution* **2016**, *218*, 595-604. DOI: https://doi.org/10.1016/j.envpol.2016.07.043.
(41) Rauert, C.; M. Shoieb; J. K. Schuster; A. Eng; T. Harner. Atmospheric concentrations and trends of poly- and perfluoroalkyl substances (PFAS) and volatile methyl siloxanes (VMS) over 7 years of sampling in the Global Atmospheric Passive Sampling (GAPS) network. *Environmental Pollution* **2018**, *238*, 94-102. DOI: https://doi.org/10.1016/j.envpol.2018.03.017.
(42) Harner, T. 2017_v1_5_Template for calculating Effective Air Sample Volumes for PUF and SIP Disk Samplers_Sept_15. 2017.
(43) Ahrens, L.; T. Harner; M. Shoeib; M. Koblizkova; E. J. Reiner. Characterization of two passive air samplers for per- and polyfluoroalkyl substances. *Environ Sci Technol* **2013**, *47* (24), 14024-14033. DOI: 10.1021/es4048945 From NLM.
(44) Rauert, C.; J. K. Schuster; A. Eng; T. Harner. Global Atmospheric Concentrations of Brominated and Chlorinated Flame Retardants and Organophosphate Esters. *Environmental Science & Technology* **2018**, *52* (5), 2777-2789. DOI: 10.1021/acs.est.7b06239.
(45) Braune, B. M.; R. J. Letcher. Perfluorinated Sulfonate and Carboxylate Compounds in Eggs of Seabirds Breeding in the Canadian Arctic: Temporal Trends (1975–2011) and Interspecies Comparison. *Environmental Science & Technology* **2013**, *47* (1), 616-624. DOI: 10.1021/es303733d.
(46) Bradford, M. M. A rapid and sensitive method for the quantitation of microgram quantities of protein utilizing the principle of protein-dye binding. *Analytical Biochemistry* **1976**, *72* (1), 248-254. DOI: https://doi.org/10.1016/0003-2697(76)90527-3.
(47) Gessaman, J. A.; S. W. Hoffman. Body Temperatures of Migrant Accipiter Hawks Just after Flight. *The Wilson Bulletin* **1990**, *102* (1), 133-137. (acccessed 2021/05/03).JSTOR.
(48) McNab, B. An Analysis of the Body Temperatures of Birds. *The Condor* **1966**, *68*. DOI: 10.2307/1365174.

(49) Prinzinger, R.; A. Preßmar; E. Schleucher. Body temperature in birds. *Comparative Biochemistry and Physiology Part A: Physiology* **1991**, *99* (4), 499-506. DOI: https://doi.org/10.1016/0300-9629(91)90122-S.
(50) Rybalov, L.; T. Rossolimo; W. Block. Temperature Adaptations of Terrestrial Arthropods of the Yenisey Region of Siberia (Asian Ecological Transect). In *Personal, societal, and ecological values of wilderness: Sixth World Wilderness Congress* Bangalore, India, 2000; Watson, A. E., Aplet, G. H., Hendee, J. C., Eds.; U.S.Department of Agriculture, Forest Service, Rocky Mountain Research Station: Vol. 2.
(51) Daniel, O.; L. Kohli; M. Bieri. Weight gain and weight loss of the earthworm Lumbricus terrestris L. at different temperatures and body weights. *Soil Biology and Biochemistry* **1996**, *28* (9), 1235-1240. DOI: https://doi.org/10.1016/0038-0717(96)00121-6.
(52) Reynolds, J. W. Earthworms of the world. *Global Biodiversity* **1994**, *4* (1), 11-16.
(53) Kim, M.; L. Y. Li; J. R. Grace; C. Yue. Selecting reliable physicochemical properties of perfluoroalkyl and polyfluoroalkyl substances (PFASs) based on molecular descriptors. *Environmental Pollution* **2015**, *196*, 462-472. DOI: https://doi.org/10.1016/j.envpol.2014.11.008.
(54) Ahrens, L.; T. Harner; M. Shoeib; M. Koblizkova; E. J. Reiner. Characterization of Two Passive Air Samplers for Per- and Polyfluoroalkyl Substances. *Environ Sci Technol* **2013**, *47* (24), 14024-14033. DOI: 10.1021/es4048945.
(55) Brusseau, M. L.; R. H. Anderson; B. Guo. PFAS concentrations in soils: Background levels versus contaminated sites. *Science of The Total Environment* **2020**, *740*, 140017. DOI: https://doi.org/10.1016/j.scitotenv.2020.140017.
(56) Bergman, M. Trophic transfer of per- and polyfluoroalkyl substances (PFASs) by glacial relicts in Lake Vättern, Sweden. Örebro University, Örebro, Sweden, 2017.
(57) Chen, M.; Q. Wang; G. Shan; L. Zhu; L. Yang; M. Liu. Occurrence, partitioning and bioaccumulation of emerging and legacy per- and polyfluoroalkyl substances in Taihu Lake, China. *Science of The Total Environment* **2018**, *634*, 251-259. DOI: https://doi.org/10.1016/j.scitotenv.2018.03.301.
(58) Chu, K.; Y. Lu; Z. Hua; Y. Liu; Y. Ma; L. Gu; C. Gao; L. Yu; Y. Wang. Perfluoroalkyl acids (PFAAs) in the aquatic food web of a temperate urban lake in East China: Bioaccumulation, biomagnification, and probabilistic human health risk. *Environmental Pollution* **2022**, *296*, 118748. DOI: https://doi.org/10.1016/j.envpol.2021.118748.
(59) Fang, S.; X. Chen; S. Zhao; Y. Zhang; W. Jiang; L. Yang; L. Zhu. Trophic Magnification and Isomer Fractionation of Perfluoroalkyl Substances in the Food Web of Taihu Lake, China. *Environmental Science & Technology* **2014**, *48* (4), 2173-2182. DOI: 10.1021/es405018b.
(60) Li, Y.; J. Yao; J. Zhang; Y. Pan; J. Dai; C. Ji; J. Tang. First Report on the Bioaccumulation and Trophic Transfer of Perfluoroalkyl Ether Carboxylic Acids in Estuarine Food Web. *Environmental Science & Technology* **2022**, *56* (10), 6046-6055. DOI: 10.1021/acs.est.1c00965.
(61) Loi, E. I. H.; L. W. Y. Yeung; S. Taniyasu; P. K. S. Lam; K. Kannan; N. Yamashita. Trophic Magnification of Poly- and Perfluorinated Compounds in a Subtropical Food Web. *Environmental Science & Technology* **2011**, *45* (13), 5506-5513. DOI: 10.1021/es200432n.
(62) Martin, J. W.; D. M. Whittle; D. C. G. Muir; S. A. Mabury. Perfluoroalkyl Contaminants in a Food Web from Lake Ontario. *Environmental Science & Technology* **2004**, *38* (20), 5379-5385. DOI: 10.1021/es049331s.
(63) Mazzoni, M.; C. Ferrario; R. Bettinetti; R. Piscia; D. Cicala; P. Volta; K. Borgå; S. Valsecchi; S. Polesello. Trophic Magnification of Legacy (PCB, DDT and Hg) and Emerging Pollutants (PFAS) in the Fish Community of a Small Protected Southern Alpine Lake (Lake Mergozzo, Northern Italy). *Water* **2020**, *12* (6). DOI: 10.3390/w12061591.
(64) Miranda, D. A.; J. P. Benskin; R. Awad; G. Lepoint; J. Leonel; V. Hatje. Bioaccumulation of Per- and polyfluoroalkyl substances (PFASs) in a tropical estuarine food web. *Science of The Total Environment* **2021**, *754*, 142146. DOI: https://doi.org/10.1016/j.scitotenv.2020.142146.

(65) Munoz, G.; H. Budzinski; M. Babut; H. Drouineau; M. Lauzent; K. L. Menach; J. Lobry; J. Selleslagh; C. Simonnet-Laprade; P. Labadie. Evidence for the Trophic Transfer of Perfluoroalkylated Substances in a Temperate Macrotidal Estuary. *Environmental Science & Technology* **2017**, *51* (15), 8450-8459. DOI: 10.1021/acs.est.7b02399.
(66) Munoz, G.; L. Mercier; S. V. Duy; J. Liu; S. Sauvé; M. Houde. Bioaccumulation and trophic magnification of emerging and legacy per- and polyfluoroalkyl substances (PFAS) in a St. Lawrence River food web. *Environmental Pollution* **2022**, *309*, 119739. DOI: https://doi.org/10.1016/j.envpol.2022.119739.
(67) Pan, C.-G.; S.-K. Xiao; K.-F. Yu; Q. Wu; Y.-H. Wang. Legacy and alternative per- and polyfluoroalkyl substances in a subtropical marine food web from the Beibu Gulf, South China: Fate, trophic transfer and health risk assessment. *Journal of Hazardous Materials* **2021**, *403*, 123618. DOI: https://doi.org/10.1016/j.jhazmat.2020.123618.
(68) Penland, T. N.; W. G. Cope; T. J. Kwak; M. J. Strynar; C. A. Grieshaber; R. J. Heise; F. W. Sessions. Trophodynamics of Per- and Polyfluoroalkyl Substances in the Food Web of a Large Atlantic Slope River. *ENVIRONMENTAL SCIENCE & TECHNOLOGY* **2020**, *54* (11), 6800-6811. DOI: 10.1021/acs.est.9b05007.
(69) Ren, J. D.; A. D. Point; S. F. Baygi; S. Fernando; P. K. Hopke; T. M. Holsen; B. S. Crimmins. Bioaccumulation of polyfluoroalkyl substances in the Lake Huron aquatic food web. *SCIENCE OF THE TOTAL ENVIRONMENT* **2022**, *819*. DOI: 10.1016/j.scitotenv.2022.152974.
(70) Ren, J.; A. Point; S. Fakouri Baygi; S. Fernando; P. K. Hopke; T. M. Holsen; B. Lantry; B. Weidel; B. S. Crimmins. Bioaccumulation of perfluoroalkyl substances in a Lake Ontario food web. *Journal of Great Lakes Research* **2022**, *48* (2), 315-325. DOI: https://doi.org/10.1016/j.jglr.2021.08.013.
(71) Simonet-Laprade, C.; H. Budzinski; M. Babut; K. Le Menach; G. Munoz; M. Lauzent; B. J. D. Ferrari; P. Labadie. Investigation of the spatial variability of poly- and perfluoroalkyl substance trophic magnification in selected riverine ecosystems. *SCIENCE OF THE TOTAL ENVIRONMENT* **2019**, *686*, 393-401. DOI: 10.1016/j.scitotenv.2019.05.461.
(72) Simonnet-Laprade, C.; H. Budzinski; K. Maciejewski; K. Le Menach; R. Santos; F. Alliot; A. Goutte; P. Labadie. Biomagnification of perfluoroalkyl acids (PFAAs) in the food web of an urban river: assessment of the trophic transfer of targeted and unknown precursors and implications. *Environmental Science: Processes & Impacts* **2019**, (11), 1864-1874, 10.1039/C9EM00322C. DOI: 10.1039/C9EM00322C.
(73) Teunen, L.; L. Bervoets; C. Belpaire; M. De Jonge; T. Groffen. PFAS accumulation in indigenous and translocated aquatic organisms from Belgium, with translation to human and ecological health risk. *Environmental Sciences Europe* **2021**, *33* (1), 39. DOI: 10.1186/s12302-021-00477-z.
(74) Zhang, Z.; H. Peng; Y. Wan; J. Hu. Isomer-Specific Trophic Transfer of Perfluorocarboxylic Acids in the Marine Food Web of Liaodong Bay, North China. *Environmental Science & Technology* **2015**, *49* (3), 1453-1461. DOI: 10.1021/es504445x.
(75) Houde, M.; G. Czub; J. M. Small; S. Backus; X. Wang; M. Alaee; D. C. G. Muir. Fractionation and Bioaccumulation of Perfluorooctane Sulfonate (PFOS) Isomers in a Lake Ontario Food Web. *Environmental Science & Technology* **2008**, *42* (24), 9397-9403. DOI: 10.1021/es800906r.
(76) Langberg, H. A.; G. D. Breedveld; G. A. Slinde; H. M. Grønning; Å. Høisæter; M. Jartun; T. Rundberget; B. M. Jenssen; S. E. Hale. Fluorinated Precursor Compounds in Sediments as a Source of Perfluorinated Alkyl Acids (PFAA) to Biota. *Environmental Science & Technology* **2020**, *54* (20), 13077-13089. DOI: 10.1021/acs.est.0c04587.
(77) Du, D.; Y. Lu; Y. Zhou; Q. Li; M. Zhang; G. Han; H. Cui; E. Jeppesen. Bioaccumulation, trophic transfer and biomagnification of perfluoroalkyl acids (PFAAs) in the marine food web of the South China Sea. *Journal of Hazardous Materials* **2021**, *405*, 124681. DOI: https://doi.org/10.1016/j.jhazmat.2020.124681.

(78) Wang, P.; Y. Lu; H. Su; C. Su; A. C. Johnson; L. Yu; A. Jenkins. Managing health risks of perfluoroalkyl acids in aquatic food from a river-estuary-sea environment affected by fluorochemical industry. *Environment International* **2020**, *138*, 105621. DOI: https://doi.org/10.1016/j.envint.2020.105621.
(79) Chen, H.; J. Han; J. Cheng; R. Sun; X. Wang; G. Han; W. Yang; X. He. Distribution, bioaccumulation and trophic transfer of chlorinated polyfluoroalkyl ether sulfonic acids in the marine food web of Bohai, China. *Environmental Pollution* **2018**, *241*, 504-510. DOI: https://doi.org/10.1016/j.envpol.2018.05.087.
(80) Kelly, B. C.; M. G. Ikonomou; J. D. Blair; B. Surridge; D. Hoover; R. Grace; F. Gobas. Perfluoroalkyl Contaminants in an Arctic Marine Food Web: Trophic Magnification and Wildlife Exposure. *ENVIRONMENTAL SCIENCE & TECHNOLOGY* **2009**, *43* (11), 4037-4043. DOI: 10.1021/es9003894.
(81) Tomy, G. T.; W. Budakowski; T. Halldorson; P. A. Helm; G. A. Stern; K. Friesen; K. Pepper; S. A. Tittlemier; A. T. Fisk. Fluorinated Organic Compounds in an Eastern Arctic Marine Food Web. *Environmental Science & Technology* **2004**, *38* (24), 6475-6481. DOI: 10.1021/es049620g.
(82) Xu, J.; C.-S. Guo; Y. Zhang; W. Meng. Bioaccumulation and trophic transfer of perfluorinated compounds in a eutrophic freshwater food web. *Environmental Pollution* **2014**, *184*, 254-261. DOI: https://doi.org/10.1016/j.envpol.2013.09.011.
(83) Houde, M.; T. A. D. Bujas; J. Small; R. S. Wells; P. A. Fair; G. D. Bossart; K. R. Solomon; D. C. G. Muir. Biomagnification of Perfluoroalkyl Compounds in the Bottlenose Dolphin (Tursiops truncatus) Food Web. *Environmental Science & Technology* **2006**, *40* (13), 4138-4144. DOI: 10.1021/es060233b.
(84) Tomy, G. T.; K. Pleskach; S. H. Ferguson; J. Hare; G. Stern; G. Macinnis; C. H. Marvin; L. Loseto. Trophodynamics of some PFCs and BFRs in a western Canadian Arctic marine food web. *Environmental Science & Technology* **2009**, *43* (11), 4076-4081. DOI: 10.1021/es900162n.
(85) Gao, K.; X. Miao; J. Fu; Y. Chen; H. Li; W. Pan; J. Fu; Q. Zhang; A. Zhang; G. Jiang. Occurrence and trophic transfer of per- and polyfluoroalkyl substances in an Antarctic ecosystem. *Environmental Pollution* **2020**, *257*, 113383. DOI: https://doi.org/10.1016/j.envpol.2019.113383.
(86) Xiong, D. Bioaccumulation and Biomagnification of Perfluoroalkyl Substances (PFAS) in a Subarctic Ringed Seal Food Web in Lake Melville, Northern Labrador, Canada. University of Toronto, Toronto, ON, 2021.
(87) Huang, K.; Y. Li; D. Bu; J. Fu; M. Wang; W. Zhou; L. Gu; Y. Fu; Z. Cong; B. Hu; J. Fu; A. Zhang; G. Jiang. Trophic Magnification of Short-Chain Per- and Polyfluoroalkyl Substances in a Terrestrial Food Chain from the Tibetan Plateau. *Environ Sci Technol Lett* **2022**, *9* (2), 147-152. DOI: 10.1021/acs.estlett.1c01009.
(88) Müller, C. E.; A. O. De Silva; J. Small; M. Williamson; X. Wang; A. Morris; S. Katz; M. Gamberg; D. C. G. Muir. Biomagnification of perfluorinated compounds in a remote terrestrial food chain: Lichen-Caribou-wolf. *Environmental Science & Technology* **2011**, *45* (20), 8665–8673. DOI: 10.1021/es201353v.
(89) Kelly, B. C.; M. G. Ikonomou; J. D. Blair; B. Surridge; D. Hoover; R. Grace; F. A. P. C. Gobas. Perfluoroalkyl Contaminants in an Arctic Marine Food Web: Trophic Magnification and Wildlife Exposure. *Environmental Science & Technology* **2009**, *43* (11), 4037-4043. DOI: 10.1021/es9003894.
